# Supplementary material for: Interventions in sexual and reproductive health services addressing violence against women in low-income and middle-income countries: a mixed-methods systematic review
Source: BMJ Open. 2022 Feb 22;12(2):e051924. doi: 10.1136/bmjopen-2021-051924 (PMC8867339; doi:10.1136/bmjopen-2021-051924)
Supplement: Supplementary data [file bmjopen-2021-051924supp002.pdf]

Supplementary file 2. Characteristics of included studies by intervention category and level of evidence

Study, publication	Country	Setting	Sample characteristics	Intervention (n) vs comparison (n)	Design/	Follow up	Primary outcomes of interest for this review	Secondary outcomes of interest for this review
**Response to VAW during routine SRH consultation (n=10)**								
Vakily 2017[58]	Iran	Antenatal clinic, 32 outpatient health centres	HCPs (midwives)	2-hour HCP training computer assisted (35) vs face-to-face (35)	RCT	2 months		Knowledge and attitudes about DV
Brown 2018[32]	South Africa	HIV testing and counselling, community, NGO	HIV positive women 18+	7-minute integrated HIV-IPV consultation over phone (166) vs standard care (83)	RCT	1 month	IPV upon partner notification of serostatus, harm	Perceived safety, safety behaviours, access to HIV treatment
*Haberland 2016[38]	Kenya	HIV testing in antenatal clinic, hospital with GBV centre	HCPs (HIV testing counsellors)<br>Pregnant women 15-49	HCP training and ongoing support, 29-minute integrated HIV-IPV consultation, referral to IPV counsellor in ANC clinic (337) vs standard care (351)	RCT<br>Nested mixed-method process evaluation	1 month	Any IPV, harm	IPV screening, referrals<br>Women's knowledge, attitudes, self-esteem, perceived intervention effect, HIV care<br>Intervention acceptability
Abeid 2016[28]	Tanzania	Post-rape care service, 5 health centres and referral hospitals	HCPs (doctors, nurses, assistant medical/clinical officers)	5-day training, guidelines, infrastructure improvement (100) vs minimal intervention (53)	Controlled before-after	12 months		Knowledge and attitudes about sexual violence and post-rape care<br>Provision of post-rape care
Jayatilleke 2015[39]	Sri Lanka	Antenatal clinic, community	HCPs (midwives)	4-day training, handbook, external referral (408)	Uncontrolled before-after	6 months		Knowledge, practices, responsibility, readiness for identifying and responding to IPV, provision of referrals
Matseke 2013[46]	South Africa	HIV testing and counselling in antenatal clinic, 16 primary health care clinics	Pregnant women 18+	HPC training, 30-minute integrated ANC-IPV consultation, external referral (160)	Uncontrolled before-after	3 months	Perceived risk of becoming a victim of femicide	
Smith 2013[54]	Kenya, Ethiopia, Jordan, Democratic Republic of Congo	Post-rape care service, 35 humanitarian settings, NGO	HCPs (doctors, nurses, midwives)	4-day training, infrastructure improvement (106)	Uncontrolled before-after<br>Qualitative study	3 months		Attitudes, knowledge, skills on sexual violence and post-rape care, provision of post-rape care
Laisser 2011[45]	Tanzania	Antenatal clinic, hospital	HCPs (clinical/medical	HCP training (39), infrastructure improvement, integrated ANC-IPV	Cross sectional	3 weeks		Intervention acceptability

Study, publication	Country	Setting	Sample characteristics	Intervention (n) vs comparison (n)	Design/	Follow up	Primary outcomes of interest for this review	Secondary outcomes of interest for this review
			officers, nursing officers) Women 18+	consultation (102), external referral	Qualitative study			
Undie 2016[57]	Kenya	HIV testing in antenatal clinic, hospital with GBV centre	Women	HCP training, integrated HIV-IPV consultation (1210), assisted referral to on-site GBV centre	Cross sectional Qualitative study	7 months		IPV screening, referrals Intervention acceptability
Cristofides 2010[33]	South Africa	HIV testing and counselling, primary health care clinic	HCPs (lay counsellors) Women	HCP training (16), integrated HIV-IPV consultation (35), external referral	Qualitative study	2 weeks		Intervention acceptability
**Response to VAW during routine SRH consultation plus community engagement (n=9)**								
Cockcroft 2019[34]	Nigeria	Universal home visits, 4 communities	Pregnant women 14-49	HCP training, infrastructure improvement, integrated DV-universal home visits that discussed domestic violence, heavy work in pregnancy, ignorance of danger signs, and lack of spousal communication with pregnant women (1837) and their partners vs delayed intervention (1853)	Cluster RCT	12 months	Physical DV, pregnancy delivery, postnatal complications	Use of SRH services
Settergren 2018[60]	Tanzania	HIV/AIDS services, hospital, and health centre	Women 15-49	Systems level activities, HCP training, infrastructure improvement, integrated-HIV-GBV consultation, onsite and external referral, community, and couple education (6 facilities, 656 women) vs standard care (6 facilities, 643 women)	Cluster RCT	28 months	Any IPV	Provision of services to IPV positive patients
Wagman 2015[59]	Uganda	HIV testing and counselling, community	Women 15-49	HCP training, integrated HIV-IPV consultation, onsite referral (6 facilities, 1812 women) vs standard care (5 facilities, 2127 women)	Cluster RCT	4 years and 7 months	Physical, emotional, sexual IPV, HIV incidence	Risk behaviours and HIV disclosure
*Bott 2014[30 37 48]	Dominican Republic, Peru, Venezuela	3 family planning clinics, NGO	HCP (doctors, nurses, midwives, counsellors, social workers, psychologists, receptionists) Women 12+	Systems level activities, HCP training and ongoing support, infrastructure improvement, integrated GBV-family planning consultation, referral to onsite GBV specialist (4 clinics)	Uncontrolled before-after Qualitative study	3 years		HCPs attitudes, knowledge, readiness for identifying and responding to GBV Intervention acceptability

**Study, publication**	**Country**	**Setting**	**Sample characteristics**	**Intervention (n) vs comparison (n)**	**Design/**	**Follow up**	**Primary outcomes of interest for this review**	**Secondary outcomes of interest for this review**
Kim 2007[42 43]	South Africa	Post-rape care service, hospital	Survivors of sexual violence	Systems level activities, 2-day HCP training (334), infrastructure improvement, community education on post-rape care	Uncontrolled before-after	No information		Use, quality, and cost of post-rape care service
Bress 2018[31]	Democratic Republic of Congo	Post-rape care service, 12 primary care clinics and referral hospital	Survivors of sexual violence 12+	HCP training and ongoing support, infrastructure improvement, community education on post-rape care (13 sites, 2081 survivors)	Cross-sectional	4 years		Provision of post-rape kit
Samandari 2016[36 49]	Guinea	Family planning clinic	HCPs (nurse, midwife, counsellor, support/admin staff)<br>Women	System level activities, 7-day HCP training and ongoing support (4), integrated family planning-IPV consultation (171), external referral, community education	Cross-sectional<br>Qualitative study	4 months		IPV identification, safety planning, referrals<br>Intervention acceptability
Sithole 2018[53]	Zimbabwe	Comprehensive post-rape care service, 8 policlinics, NGO	HCPs (doctors, nurses, managers)<br>Survivors of sexual violence	HCP training (80), infrastructure improvement, post-rape care (1669), community education on post-rape care	Cross sectional service evaluation	4 years		HCPs knowledge about post-rape care<br>Provision of post-rape care
Turan 2013[56]	Kenya	Antenatal clinic, primary health care clinic	HCPs (all clinic staff including admin, community volunteers, lay health workers)<br>Pregnant women	40-hour HCP training, integrated ANC-GBV consultation (134), assisted external referral, community education	Cross sectional<br>Qualitative study	5 months		GBV identification, referrals<br>Intervention acceptability
**Response to VAW in addition to routine SRH consultation (n=7)**								
Cripe 2010[35]	Peru	Antenatal clinic, hospital	Pregnant women 18-45 with IPV experience	1* 30-minute psychosocial counselling session by social worker, resource card, external referral (110) vs minimal intervention (110)	RCT	Prenatal appointment to 1 week after delivery	Quality of life	Safety behaviours, use of community resources
Khalili 2019[40 41]	Iran	Antenatal clinic, University health centers	Pregnant women 20+ with IPV experience	4*90-minute psychoeducational sessions by counsellor (50) vs standard care (50)	RCT	2 months	Verbal and physical IPV, psychological distress	
Mutisya 2018[47]	Kenya	Antenatal clinic, 12 primary health care clinics	Pregnant women 18-45 with IPV experience	1-3*30-35-minute psychosocial counselling sessions by researcher, risk assessment, safety planning, resource card, external referral	RCT	6 months	Physical, emotional, severe combined IPV and harassment, depression	

**Study, publication**	**Country**	**Setting**	**Sample characteristics**	**Intervention (n) vs comparison (n)**	**Design/**	**Follow up**	**Primary outcomes of interest for this review**	**Secondary outcomes of interest for this review**
				(141) vs minimal intervention (142)				
Sapkota 2020[50 51]	Nepal	Antenatal clinic, hospital	Pregnant women 18+ with DV experience	1*35-45-minute psychosocial session by counsellor, resource card, contact details of the counsellor (70) vs minimal intervention (70)	RCT Nested qualitative study	Prenatal appointment to 6 weeks after delivery	Depression, anxiety, quality of life	Self-efficacy, safety behaviours, social support Intervention acceptability
Sikkema 2018[44 52]	South Africa	HIV testing and treatment, primary health care clinic	HIV positive women 18+ with experience of sexual violence	4 individual and 3 group*90-minute psychosocial training sessions by trained lay provider (32) vs standard care (32)	RCT Nested qualitative study	6 months	PTSD	Coping strategies, engagement with HIV treatment Intervention acceptability
Tanghizaden 2018[55]	Iran	Antenatal clinic, 16 health centres	Pregnant women with IPV experience	4*90-minute psychosocial training sessions on problem-solving skills by researcher (125) vs standard care (132)	RCT	3 months	Physical, psychological, sexual IPV	
Arora 2019[29]	India	Antenatal clinic, 2 hospitals	Pregnant women with DV experience	≥2*30-45-minute psychosocial sessions by counsellor (155)	Uncontrolled before-after	First prenatal appointment to 6 weeks after delivery	Physical, emotional, financial DV, physical health, emotional health	Knowledge and attitudes about DV, coping behaviours

Note. * grey literature. NGO non-governmental (third sector) organisation. RCT randomised controlled trial. HCP health care providers. IPV intimate partner violence. DV domestic violence. GBV gender-based violence. HIV human immunodeficiency viruses. PTSD posttraumatic stress disorder. SRH sexual and reproductive
