# Supplementary material for: Interventions in sexual and reproductive health services addressing violence against women in low-income and middle-income countries: a mixed-methods systematic review
Source: BMJ Open. 2022 Feb 22;12(2):e051924. doi: 10.1136/bmjopen-2021-051924 (PMC8867339; doi:10.1136/bmjopen-2021-051924)
Supplement: Supplementary data [file bmjopen-2021-051924supp003.pdf]

Online supplementary file 3. Quality appraisal

Risk of bias in randomised controlled trials

Study ID	Bias arising from the randomization process	Bias due to deviations from the intended interventions (assignment)	Bias due to deviations from the intended interventions (adherence)	Bias due to missing outcome data	Bias in measurement of the outcome	Bias in selection of the reported result	Overall risk of bias
Brown 2018[32]	Low	Some concerns	High	Low	Low	Low	High
Cockcroft 2019[34]	Low	Low	Low	Low	Low	Low	Low
Cripe 2013[35]	Low	Low	Low	Low	Some concerns	Some concerns	Some concerns
Haberland 2016[38]	Some concerns	Low	High	Low	Some concerns	Some concerns	High
Khalili 2020[40]	Low	High	High	Low	High	Low	High
Mutisya 2018[47]	Low	High	High	Low	High	Some concerns	High
Sapkota 2020[50]	Low	Low	Low	Low	Low	Low	Low
Settergren 2018[60]	Low	Low	High	Low	High	Low	High
Sikkema 2018[52]	Low	Low	Low	High	Some concerns	Some concerns	High
Tanghizadeh 2018[55]	Low	Low	Low	Low	Low	Some concerns	Some concerns
Vakily 2017[58]	Some concerns	Some concerns	High	High	Low	Some concerns	High
Wagman 2015[59]	Some concerns	High	Some concerns	Low	High	Some concerns	High

Risk of bias (EPOC criteria) in controlled before-after studies

Study ID	Was the allocation sequence adequately generated?	Was the allocation adequately concealed?	Were baseline outcome measurements similar?	Were baseline characteristics similar?	Were incomplete outcome data adequately addressed?	Was knowledge of the allocated interventions adequately prevented during the study?	Was the study adequately protected against contamination ?	Was the study free from selective outcome reporting?	Was the study free from other sources of bias?	Overall risk of bias
Abeid 2016[28]	No	No	Yes	Yes	Yes	Unclear	Unclear	Yes	Unclear	High

Risk of bias (EPOC criteria) in studies without a control group

Study ID	Was the intervention independent of other changes?	Was the shape of the intervention effect pre-specified?	Was the intervention unlikely to affect data collection?	Was knowledge of the allocated interventions adequately prevented during the study?	Were incomplete outcome data adequately addressed?	Was the study free from selective outcome reporting?	Was the study free from other risk of bias?	Overall risk of bias
Arora 2019[29]	No	Yes	Yes	No	Yes	No	Unclear	High
Bott 2004[30]	Unclear	Unclear	Yes	No	No	Unclear	Unclear	High
Bress 2019[31]	No	No	No	No	Unclear	Yes	Unclear	High

Study ID	Was the intervention independent of other changes?	Was the shape of the intervention effect pre-specified?	Was the intervention unlikely to affect data collection?	Was knowledge of the allocated interventions adequately prevented during the study?	Were incomplete outcome data adequately addressed?	Was the study free from selective outcome reporting?	Was the study free from other risk of bias?	Overall risk of bias
Jayatilleke 2015[39]	Unclear	Yes	Yes	No	Unclear	Yes	Unclear	High
Kim 2007[42]	No	Yes	Yes	No	Unclear	No	No	High
Laisser 2011[45]	No	No	No	No	Unclear	Yes	Unclear	High
Matseke 2013[46]	Unclear	Yes	Yes	No	No	Yes	No	High
Samandari 2016[49]	No	Yes	Yes	No	Yes	Yes	Unclear	High
Sithole 2018[53]	Unclear	No	No	No	Unclear	No	Unclear	High
Smith 2013[54]	No	Yes	Yes	Unclear	Yes	Yes	No	High
Turan 2013[56]	Unclear	No	No	No	Unclear	Yes	Unclear	High
Undie 2016[57]	No	No	No	No	Unclear	Unclear	Unclear	High

Quality appraisal of qualitative studies

CASP signalling questions	Bott 2004[30]	Christofides 2010[33]	Haberland 2016[38]	Laisser 2011[45]	Samandari 2016[49]	Sapkota 2020[51]	Sikkema 2018[44]	Smith 2013[54]	Turan 2013[56]	Undie 2016[57]
1. Interprets subjective experiences?	Yes	Yes	Yes	Yes	Yes	Yes	Yes	Yes	Yes	Yes
2. Right methodology?	Yes	Yes	Yes	Yes	Yes	Yes	Yes	Yes	Yes	Yes
3. Appropriate design?	Yes	Yes	Yes	Yes	Yes	Yes	Yes	Yes	Yes	Yes
4. Design justified?	Yes	No	No	Yes	No	Yes	Yes	No	No	No
5. Ethical issues considered?	No	No	Yes	Yes	Yes	Yes	Yes	Yes	Yes	Yes
6. Credibility established?	Yes	No	No	Yes	Yes	Yes	Yes	Yes	Yes	Yes
7. Transferability established?	No	Yes	No	Yes	Yes	Yes	Yes	Yes	No	No
8. Purpose established?	Yes	Yes	Yes	Yes	Yes	Yes	Yes	Yes	No	Yes
9. Recruitment appropriate?	Yes	Yes	Yes	Yes	Yes	Yes	Yes	Yes	Yes	Yes
10. Selection of participants explained?	No	Yes	Yes	Yes	Yes	Yes	Yes	No	Yes	No
11. Participants appropriate?	No	No	No	Yes	Yes	No	Yes	Yes	Yes	No
12. Discussed recruitment?	No	Yes	No	No	No	No	Yes	No	No	No
13. Justified setting?	Yes	Yes	Yes	Yes	Yes	Yes	Yes	Yes	Yes	No
14. How data were collected?	Yes	Yes	Yes	Yes	Yes	Yes	Yes	Yes	Yes	Yes
15. Justified data collection method?	No	No	Unsure	Yes	No	Yes	Yes	No	No	No

CASP signalling questions	Bott 2004[30]	Christofides 2010[33]	Haberland 2016[38]	Laisser 2011[45]	Samandari 2016[49]	Sapkota 2020[51]	Sikkema 2018[44]	Smith 2013[54]	Turan 2013[56]	Undie 2016[57]
16. Described data collection method?	No	Yes	Yes	Yes	Yes	Yes	Yes	Yes	Yes	Yes
17. Form of data clear?	No	Yes	Yes	Yes	Yes	Yes	Yes	Yes	Yes	Yes
18. Described how data were reduced/transformed for analysis?	No	No	No	Yes	Yes	Yes	Yes	Yes	Yes	Yes
19. Discussed interpretation of findings?	Yes	No	No	Yes	Yes	Yes	Yes	No	Yes	Yes
20. Ensured neutrality?	No	No	No	No	Yes	Yes	Yes	No	Yes	No
**Total (Yes/No/Unsure)**	**10/10/0**	**12/8/0**	**11/8/1**	**18/2/0**	**17/3/0**	**18/2/0**	**20/0/0**	**14/6/0**	**15/5/0**	**12/8/0**
