# Supplementary material for: Interventions in sexual and reproductive health services addressing violence against women in low-income and middle-income countries: a mixed-methods systematic review
Source: BMJ Open. 2022 Feb 22;12(2):e051924. doi: 10.1136/bmjopen-2021-051924 (PMC8867339; doi:10.1136/bmjopen-2021-051924)
Supplement: Supplementary data [file bmjopen-2021-051924supp006.pdf]

Supplementary file 6. Effects and outcomes of interventions on response to VAW in addition to routine SRH consultation

Outcomes	Results	Effect (95% CI)	N of participants (design/follow up)	Study	Risk of bias	Direction of effect/interpretation
**Direct effects on health-related cognition and emotions**						
**Women knowledge about VAW (n=1)**						
Proportion who recognised violence as an issue of power, post-intervention	60.6%		155 women (UBA/ First prenatal appointment to 6 weeks after delivery)	Arora 2019[29]	High	After 2 or more 30-45-minute psychosocial counselling sessions, around 60% of pregnant women were aware about domestic violence and its impact on health
Proportion who recognised the impact of violence on health, post-intervention	65.5%					
**Women readiness for addressing VAW (n=1)**						
Proportion who recognised the need to take steps to address violence, post intervention	59.9%		155 women (UBA/ First prenatal appointment to 6 weeks after delivery)	Arora 2019[29]	High	After 2 or more 30-45-minute psychosocial counselling sessions, 60% of pregnant women were ready to address VAW
**Intermediate effects on health-related behaviour and practices**						
**VAW referral uptake (n=1)**						
Proportion (n) who used specialist IPV services, post-intervention	Intervention = 0.96% (1/104)<br>Control = 1.00% (1/100)	-	110 women (RCT/Prenatal appointment to 1 week after delivery)	Cripe 2010[35]	Some concerns	1 30-minute psychosocial counselling session, resource card, external referral had no effect on uptake of external referrals
**Use of SRH services (n=1)**						
Proportion (n) who missed antiretroviral medication, post-intervention	Intervention = 42.3% (19)<br>Control = 36.4% (25)	-	32 women (RCT/6 months)	Sikkema 2018[52]	High	7 90-minute psychosocial sessions on coping had no effect on engagement with HIV treatment among women with a history of sexual violence
Proportion (n) with high levels of non-retention in care, post-intervention	Intervention = 42.3% (26)<br>Control = 33.3% (27)	-				
**Use of non-health services (n=1)**						
Proportion (n) who used legal services, post-intervention	Intervention = 1.92% (2/104)<br>Control = 3.00% (3/100)	-	110 women (RCT/Prenatal appointment to 1 week after delivery)	Cripe 2010[35]	Some concerns	1 30-minute psychosocial counselling session, resource card, external referral had no effect on use of community resources among pregnant women
Proportion (n) who used police, post-intervention	Intervention = 0.96% (1/104)<br>Control = 4.00% (4/100)	-				
Proportion (n) who used social services, post-intervention	Intervention = 1.92% (2/104)<br>Control = 2.00% (2/100)	-				
**Safety behaviour (n=4)**						
Mean (SD) score of using safety behaviours, post-intervention	Intervention = 9.50 (2.63)<br>Control = 7.74 (2.42)	MD = 2.41 (1.43; 3.40)	70 women (RCT/Prenatal appointment to 6 weeks after delivery)	Sapkota 2020[50]	Low	1 35-45-minute psychosocial counselling session, resource card, contact details of the counsellor increased use of safety behaviours among pregnant women

Outcomes	Results	Effect (95% CI)	N of participants (design/follow up)	Study	Risk of bias	Direction of effect/interpretation
Proportion who adopted safety behaviours, post-intervention	Intervention = 30.3%<br>Control = 11.2%		110 women (RCT/Prenatal appointment to 1 week after delivery)	Cripe 2010[35]	Some concerns	1 30-minute psychosocial counselling session, resource card, external referral had no effect on safety behaviours among pregnant women
Mean (SD) score of avoidance, coping post-intervention	Intervention = 2.17 (0.13)<br>Control = 1.99 (0.09)	-	32 women (RCT/6 months)	Sikkema 2018[52]	High	7 90-minute psychosocial training sessions reduced avoidance coping, but had no effect on social coping among women with a history of sexual violence
Mean (SD) score of social coping, post-intervention	Intervention = 2.90 (0.10)<br>Control = 2.58 (0.10)	-				
Proportion (n) who used adaptive coping strategies at individual level pre- and post-intervention	Pre-intervention = 51.4% (73)<br>Post-intervention = 59.1% (84)<br>p=0.193		155 women (UBA/ First prenatal appointment to 6 weeks after delivery)	Arora 2019[29]	High	2 or more 30-45-minute psychosocial counselling sessions had no effect on coping behaviours among pregnant women
Proportion who used adaptive coping strategies at informal and formal levels pre- and post-intervention	Pre-intervention = 85.2% (121)<br>Post-intervention = 86.6% (123)<br>p=0.832					
**Health outcomes**						
**Re-exposure to VAW (n=4)**						
Mean (SD) score of verbal and physical IPV, post-intervention	Intervention = 11.62 (2.05)<br>Control = 13.28 (1.94)<br>p<0.001	-	50 pregnant women (RCT/2 months)	Khalili 2019[40]	High	4 90-minute psychoeducational counselling sessions reduced verbal and physical IPV among pregnant women
Mean (SD) score of total IPV, post-intervention	Intervention = 17.70 (11.12)<br>Control = 31.22 (21.17)	MD = 13.51 (9.99; 17.02)	141 pregnant women (RCT/6 months)	Mutisya 2018[47]	High	1-3 30-35-minute psychosocial counselling sessions, risk assessment, safety planning, resource card, external referral reduced IPV among pregnant women
Proportion who experienced physical IPV, post intervention	Intervention = 51.2%<br>Control = 65.9%	RR = 0.78 (0.63; 0.93)	125 women (RCT/3 months)	Tanghizaden 2018[55]	Some concerns	4 90-minute psychosocial training sessions on problem-solving skills reduced physical and psychological IPV but had no effect on sexual IPV among pregnant women
Proportion who experienced psychological IPV, post intervention	Intervention = 67.4%<br>Control = 92.4%	RR = 0.73 (0.64; 0.83)				
Proportion who experienced sexual IPV, post intervention	Intervention = 50.4%<br>Control = 57.6%	RR = 0.87 (0.69; 1.09)				
Change in proportion who experienced physical domestic violence, before-after	74.6% to 3.5%	-	155 women (UBA/ First prenatal appointment to 6 weeks after delivery)	Arora 2019[29]	High	2 or more 30-45-minute psychosocial counselling sessions reduced physical, emotional, and financial domestic violence among pregnant women

Outcomes	Results	Effect (95% CI)	N of participants (design/follow up)	Study	Risk of bias	Direction of effect/interpretation
Change in proportion who experienced emotional domestic violence, before-after	98.6% to 34.5%	-				
Change in proportion who experienced financial domestic violence, before-after	72.5% to 11.3%	-				
**Mental health (n=6)**						
Mean (SD) score of anxiety, post-intervention	Intervention = 4.33 (3.84)<br>Control = 6.93 (4.87)	MD = -3.73 (-5.42; -2.04)	70 women (RCT/prenatal appointment to 6 weeks after delivery)	Sapkota 2020[50]	Low	1 35-45-minute psychosocial counselling session, resource card, contact details of the counsellor reduced anxiety and depression among pregnant women
Mean (SD) score of depression, post-intervention	Intervention = 3.51 (3.46)<br>Control = 6.13 (3.68)	MD = -3.41 (-4.84; -1.99)				
Mean (SD) score of postnatal depression, post-intervention	Intervention = 5.34 (4.23)<br>Control = 12.46 (4.22)	MD = 7.12 (6.21; 8.03)	141 women (RCT/6 months)	Mutisya 2018[47]	High	1-3 30-35-minute psychosocial counselling sessions with risk assessment, safety planning, external referral, and resource card reduced depression among pregnant women
Mean (SD) score of PTSD, post-intervention	Intervention = 28.61 (5.04)<br>Control = 22.50 (3.47)	-	32 women (RCT/6 months)	Sikkema 2018[52]	High	7 90-minute psychosocial training sessions had no effect on PTSD symptoms among women with a history of sexual violence
Mean (SD) score of psychological distress, post-intervention	Intervention = 22.28 (3.81)<br>Control = 24.06 (4.16)<br>p<0.001	-	50 women (RCT/2 months)	Khalili 2019[40]	High	4 90-minute psychoeducational counselling sessions reduced psychological distress among pregnant women
Difference between baseline and post-intervention mean (SD) score for mental health	Intervention = 2.50 (20.95)<br>Control = 2.04 (19.61)	MD = 4.54 (-1.07; 10.15)	110 women (RCT/Prenatal appointment to 1 week after delivery)	Cripe 2010[35]	Some concerns	1 30-minute psychosocial counselling session, resource card, external referral had no effect on mental health of pregnant women
Change in proportion who experienced any emotional health problems	96.5% to 33.1%	-	155 women (UBA/ First prenatal appointment to 6 weeks after delivery)	Arora 2019[29]	High	2 or more 30-45-minute psychosocial counselling sessions reduced % of pregnant women with emotional health problems.
**Sexual and reproductive health (n=1)**						
Proportion (n) of those with unsuppressed HIV viral load, post-intervention	Intervention = 15.8% (19)<br>Control = 20.0% (25)<br>($\chi2$ (1) = 0.13, p = 0.72)	-	32 women (RCT/6 months)	Sikkema 2018[52]	High	7 90-minute psychosocial training sessions had no effect on adherence to therapy measured by HIV viral load among women with a history of sexual abuse
**Physical health (n=2)**						
Difference between baseline and post-intervention mean (SD) score for general health	Intervention = 5.30 (15.62)<br>Control = 4.74 (14.67)	MD = 0.05 (-6.80; 7.79)	110 women (RCT/Prenatal appointment to 1 week after delivery)	Cripe 2010[35]	Some concerns	1 30-minute psychosocial counselling session, resource card, external referral had no effect on general health

Outcomes	Results	Effect (95% CI)	N of participants (design/follow up)	Study	Risk of bias	Direction of effect/interpretation
Change in proportion who experienced any physical health problems	54.6% to 10.5%	-	155 women (UBA/ First prenatal appointment to 6 weeks after delivery)	Arora 2019[29]	High	2 or more individual psychosocial counselling sessions reduced % of pregnant women with physical health problems.
**Quality of life (n=2)**						
Mean (SD) score of overall quality of life, post-intervention	Intervention = 17.22 (3.00)<br>Control = 15.19 (2.77)	MD = 2.45 (1.51; 3.39)	70 women (RCT/prenatal appointment to 6 weeks after delivery)	Sapkota 2020[50]	Low	1 35-45-minute psychosocial counselling session, resource card, contact details of the counsellor improved quality of life among pregnant women
Difference between baseline and post-intervention mean (SD) score for physical functioning	Intervention = -15.67 (28.35)<br>Control = -15.70 (25.06)	MD = 0.03 (-7.37; 7.42)	110 women (RCT/Prenatal appointment to 1 week after delivery)	Cripe et 2010[35]	Some concerns	1 30-minute psychosocial counselling session, resource card, external referral had no effect on general health, bodily pain, vitality, social functioning among pregnant women.
Difference between baseline and post-intervention mean (SD) score for bodily pain	Intervention = -7.40 (28.33)<br>Control = -7.90 (24.28)	MD = 0.50 (-6.80; 7.79)				
Difference between baseline and post-intervention mean (SD) score for vitality	Intervention = -0.19 (22.34)<br>Control = -3.65 (22.06)	MD = 3.46 (-2.67; 9.59)				
Difference between baseline and post-intervention mean (SD) score for social functioning	Intervention = -0.36 (34.94)<br>Control = -3.50 (37.06)	MD = 3.14 (-6.80; 13.08)				

Note. VAW violence against women. GBV gender-based violence. DV domestic violence. IPV intimate partner violence. SRH sexual and reproductive health. STI Sexually transmitted infections. HIV human immunodeficiency virus. ANC antenatal care. HCP health care provider. RCT randomised controlled trial. CBA controlled before-after study. UBA uncontrolled before-after study. CI confidence interval. SD standard deviation. IQR interquartile range. MD mean difference. RR relative risk.
