# Supplementary material for: Interventions in sexual and reproductive health services addressing violence against women in low-income and middle-income countries: a mixed-methods systematic review
Source: BMJ Open. 2022 Feb 22;12(2):e051924. doi: 10.1136/bmjopen-2021-051924 (PMC8867339; doi:10.1136/bmjopen-2021-051924)
Supplement: Author's manuscript [file bmjopen-2021-051924.draft_revisions.pdf]

BMJ Open is committed to open peer review. As part of this commitment we make the peer review history of every article we publish publicly available.

When an article is published we post the peer reviewers' comments and the authors' responses online. We also post the versions of the paper that were used during peer review. These are the versions that the peer review comments apply to.

The versions of the paper that follow are the versions that were submitted during the peer review process. They are not the versions of record or the final published versions. They should not be cited or distributed as the published version of this manuscript.

BMJ Open is an open access journal and the full, final, typeset and author-corrected version of record of the manuscript is available on our site with no access controls, subscription charges or pay-per-view fees (http://bmjopen.bmj.com).

If you have any questions on BMJ Open's open peer review process please email info.bmjopen@bmj.com

BMJ Open

INTERVENTIONS IN SEXUAL AND REPRODUCTIVE HEALTH SERVICES ADDRESSING VIOLENCE AGAINST WOMEN IN LOW- AND MIDDLE- INCOME COUNTRIES: A MIXED-METHODS SYSTEMATIC REVIEW

Journal:	*BMJ Open*
Manuscript ID	bmjopen-2021-051924
Article Type:	Original research
Date Submitted by the Author:	01-Apr-2021
Complete List of Authors:	Lewis, Natalia; University of Bristol, Bristol Medical School (PHS)<br>Munas, Muzrif; University of Peradeniya; University of Bristol Faculty of Health Sciences, Bristol Medical School (PHS)<br>Colombini, Manuela; London School of Hygiene and Tropical Medicine, Department of Global Health and Development<br>D'Oliveira, AF; University of São Paulo Institute of Biomedical Sciences, Faculty of Medicine<br>Pereira, Stephanie ; University of São Paulo Institute of Biomedical Sciences, Faculty of Medicine<br>Shrestha, Satya; Kathmandu University School of Medical Sciences; University of Bristol<br>Rajapakse, Thilini; University of Peradeniya<br>Shaheen, Amira ; An-najah National University Faculty of Medicine and Health Sciences<br>Rishal , Poonam; Kathmandu University School of Medical Sciences<br>Alkaiyat, Abdulsalam ; An-najah National University Faculty of Medicine and Health Sciences<br>Richards, Alison; University of Bristol, Bristol Medical School (PHS); NIHR ARC West, University Hospitals Bristol NHS Foundation Trust<br>Garcia-Moreno, Claudia; Organisation mondiale de la Sante, Department of Reproductive Health and Research<br>Feder, Gene; University of Bristol Faculty of Health Sciences, Centre for Academic Primary Care<br>Bacchus, Loraine; London School of Hygiene and Tropical Medicine Faculty of Public Health and Policy
Keywords:	PUBLIC HEALTH, REPRODUCTIVE MEDICINE, SEXUAL MEDICINE, Organisation of health services < HEALTH SERVICES ADMINISTRATION & MANAGEMENT, OBSTETRICS

SCHOLARONE™
Manuscripts

I, the Submitting Author has the right to grant and does grant on behalf of all authors of the Work (as defined in the below author licence), an exclusive licence and/or a non-exclusive licence for contributions from authors who are: i) UK Crown employees; ii) where BMJ has agreed a CC-BY licence shall apply, and/or iii) in accordance with the terms applicable for US Federal Government officers or employees acting as part of their official duties; on a worldwide, perpetual, irrevocable, royalty-free basis to BMJ Publishing Group Ltd ("BMJ") its licensees and where the relevant Journal is co-owned by BMJ to the co-owners of the Journal, to publish the Work in this journal and any other BMJ products and to exploit all rights, as set out in our licence.

The Submitting Author accepts and understands that any supply made under these terms is made by BMJ to the Submitting Author unless you are acting as an employee on behalf of your employer or a postgraduate student of an affiliated institution which is paying any applicable article publishing charge ("APC") for Open Access articles. Where the Submitting Author wishes to make the Work available on an Open Access basis (and intends to pay the relevant APC), the terms of reuse of such Open Access shall be governed by a Creative Commons licence – details of these licences and which Creative Commons licence will apply to this Work are set out in our licence referred to above.

Other than as permitted in any relevant BMJ Author's Self Archiving Policies, I confirm this Work has not been accepted for publication elsewhere, is not being considered for publication elsewhere and does not duplicate material already published. I confirm all authors consent to publication of this Work and authorise the granting of this licence.

For peer review only

INTERVENTIONS IN SEXUAL AND REPRODUCTIVE HEALTH SERVICES ADDRESSING VIOLENCE AGAINST WOMEN IN LOW- AND MIDDLE- INCOME COUNTRIES: A MIXED-METHODS SYSTEMATIC REVIEW

Natalia V Lewis,[1] Muzrif Munas,[1,2] Manuela Colombini,[3] Ana Flavia d'Oliveira,[4] Stephanie Pereira,[4] Satya Shrestha,[1,5] Thilini Rajapakse,[2] Amira Shaheen,[6] Poonam Rishal,[5] Abdulsalam Alkaiyat,[6] Alison Richards,[1,7] Claudia M García-Moreno,[8] Gene S Feder,[1] Loraine J Bacchus[3]

[1]Centre for Academic Primary Care, Population Health Sciences, Bristol Medical School, University of Bristol, UK

[2]University of Peradeniya, Faculty of Medicine, University of Peradeniya, Sri Lanka

[3]Department of Global Health and Development, Faculty of Public Health and Policy, London School of Hygiene & Tropical Medicine, UK

[4]Faculty of Medicine, University of São Paulo, Brazil

[5]School of Medical Sciences, Kathmandu University, Nepal

[6]Public Health Department, Faculty of Medicine and Health Sciences, An-Najah National University, Nablus, Occupied Palestinian Territories

[7]The National Institute for Health Research Applied Research Collaboration West (NIHR ARC West) at University Hospitals Bristol and Weston NHS Foundation Trust, UK

[8]Department of Sexual and Reproductive Health and Research, World Health Organisation

Correspondence to: Dr Natalia V Lewis, nat.lewis@bristol.ac.uk

Word count: 4,990

For peer review only

Key words: domestic violence, gender-based violence, intimate partner violence, violence against women, developing countries, low- and middle-income countries, systematic review, reproductive health services.

ABSTRACT

Objectives. Violence against women (VAW) is highly prevalent in low- and middle-income countries (LMICs). Sexual and reproductive health (SRH) services play a crucial role in the multi-sector response to VAW through identification of survivors, provision of first-line support, and referral/signposting to specialist services. We assessed effectiveness of VAW interventions in SRH services in LMICs.

Methods. Mixed-methods systematic review with narrative quantitative and thematic qualitative syntheses, generating line of argument, mapping onto logic model.

Results. 26 studies (16 quantitative, 9 mixed-methods, 1 qualitative) of varied interventions using heterogeneous measures. Of ten interventions that strengthened health care provider (HCP) capacity to respond to VAW during routine SRH consultation, three reported no harm and reduction in some types of violence. Of nine interventions that strengthen HCP and community capacity to respond to VAW during routine SRH consultation, three reported conflicting effects on some VAW and mixed effect on SRH. The interventions increased identification of VAW, but had null effects on the provision and uptake of referrals to VAW services. The rates of provided referrals and first-line support were 75-100%; the uptake by women was 0.6-53%. Of seven psychosocial interventions in addition to SRH consultation that strengthened women's capacity to address VAW, four reduced re-exposure to some types of VAW and improved health. Accepting attitudes towards VAW, fear of consequences, and limited readiness of the society, care systems, and individuals disrupted the pathway to outcomes. No study evaluated cost-effectiveness.

Conclusions. Some VAW interventions in SRH services reduced re-exposure to some types of VAW and improved some health outcomes in single studies. Future interventions should strengthen capacity to address VAW across community, SRH service, and individual HCPs and women. First-line support should be better tailored to women's needs and expectations. Future evaluations should use robust design, longer follow-up, and standardised outcomes measures.

PROSPERO registration number CRD42019137167.

STRENGTHS AND LIMITATIONS OF THIS STUDY

- This review was carried out by a team of researchers from the UK and low- and middle-income countries with expertise and experience in healthcare system responses to violence against women and global health.

For peer review only

- Inclusion of peer-reviewed and grey reports of studies of any design resulted in selection of the most relevant studies.
- The logic model approach to the synthesis of findings produced evidence in a format understandable to the end users of this review.
- Most included studies had methodological limitations and high risk of bias.
- We could not perform meta-analysis of quantitative findings because primary studies evaluated varied interventions and used different instruments to measure varied outcomes.

INTRODUCTION

Violence against women (VAW) is a violation of global health and human rights.[1] The most common forms are intimate partner violence (IPV) and non-partner sexual violence (NPSV). One in three women worldwide have experienced physical and/or sexual violence, mostly by an intimate partner. VAW is more prevalent in low- and middle-income countries (LMICs). Exposure to VAW is associated with mental and physical health problems, including increased sexually transmitted infections (STI) and HIV, unplanned pregnancy and abortion, gynaecological conditions.[2 3]

The healthcare system has a key role in a multi-sectoral response to VAW because most women attend sexual and reproductive health (SRH) services at some point.[4 5] Healthcare providers (HCPs) are uniquely placed to identify victims/survivors, provide first-line support and clinical care, and connect them with other services.[6] The readiness of healthcare systems to respond to VAW depends on the availability and strength of: (i) leadership and governance, (ii) multi-sectoral coordination, (iii) workforce development, (iv) healthcare delivery, (v) infrastructure, (vi) financing, (vii) monitoring and evaluation.[7 8]

Systematic reviews found scant evidence from LMICs on effectiveness of VAW interventions in healthcare.[9 10] This study answers two questions: (i) What is the evidence for effectiveness and cost-effectiveness of interventions in SRH services that address VAW? (ii) What are the barriers to the effectiveness?

METHODS

We conducted a systematic review following the Cochrane[11] and PRISMA guidelines.[12] The mixed-methods design allowed integration of diverse types of evidence to inform VAW research and intervention development in LMICs. We configured qualitative and quantitative findings into a line of argument[13] and mapped two syntheses onto the logic model.[14] The study protocol was registered with PROSPERO (CRD42019137167).

Search strategy and selection criteria

We included primary studies in any language with English abstract since 2005, the year of the first published evaluation of VAW interventions in SRH services (expert opinion from the study advisory

For peer review only

group). We identified earlier studies through reference checking. We used terminology and definitions from WHO guidance on healthcare system responses to VAW (Table 1).[15]

Table1. Study inclusion and exclusion criteria with justification

	Inclusion criteria	Exclusion criteria
Participants	Recipients of healthcare services - women of reproductive age (15-49 years old)<br>AND/OR<br>Providers of healthcare services – organisations (e.g., hospital, clinic, primary care centre, other service delivery points) or individuals (e.g., health care professional, community health worker, or any other person who is trained to deliver healthcare in their community).<br>Studies which recruited only a subset of recipients or providers of healthcare services.	Female children and girls under 15 years old. While recognizing that pregnancies occur among young adolescents 10-14, most studies consider women aged 15-49 years as the main group using SRH services in LMICs.
Interventions	Any intervention addressing VAW.<br>These are complex interventions[16] aimed to identify women affected by violence, provide first-line support, clinical care, and signpost or refer to available community support services including specialist VAW services.<br>Any definition of VAW, including any type of IPV, domestic violence and abuse, family violence or NPSV against a woman, including transgender women.	No intervention<br>Hypothetical intervention addressing VAW. We are synthesising evidence of interventions that have been tested.<br>Female genital mutilation/cutting, trafficking. These type of VAW were addressed in recent systematic reviews.[17 18]<br>'Honour'-based violence, forced marriage. There is an overlap between IPV, domestic violence and abuse and 'honour'-based violence and forced marriage. Therefore, we will capture relevant studies through including papers on IPV and domestic violence and abuse.
Comparators	Controlled studies: usual care, no VAW intervention, delayed VAW intervention, minimal intervention (e.g., information provision).<br>Uncontrolled studies: group before the intervention.<br>No control group.	
Outcomes	Primary outcomes: any health outcomes for survivors of VAW, any type of VAW, any	

For peer review only - http://bmjopen.bmj.com/site/about/guidelines.xhtml

	Inclusion criteria	Exclusion criteria
	harms, cost effectiveness of VAW interventions.<br>AND/OR<br>Secondary outcomes: patient and professional knowledge, attitudes, skills/practices regarding VAW; provision and uptake of VAW and SRH services.<br>Phenomenon of interest: provider and recipient experiences of and views on the acceptability of VAW interventions.	
Study type	Primary studies of any designs. Primary studies that used quantitative designs such as randomised controlled trials, controlled and uncontrolled before-after studies, interrupted time series studies, cross-sectional studies. Primary studies that used qualitative designs such as ethnographic research, interview or focus-group based studies, case studies, process evaluations and mixed methods designs. We include these studies if they had used qualitative methods for data collection and analysis and reported quotes from participants.<br>Mixed-methods studies.	Systematic reviews. We used systematic reviews to identify potentially eligible primary studies.
Context	Studies conducted in SRH services in a country defined as LMIC by the World Bank,[19] including humanitarian settings. Depending on country context, SRH services can be delivered at any level of healthcare provision and usually include contraceptive services, maternal and perinatal health, treatment for STI, HIV and reproductive tract infections, abortion, fertility treatment and gynaecological treatment.	
Report type	Full-text peer reviewed studies, conference abstracts, grey literature, unpublished studies.	Animal studies, opinion pieces, editorials, and publication which did not report primary data.

For peer review only

VAW violence against women, LMICs low-income and middle-income countries, SRH sexual and reproductive health, HCPs health care providers, IPV intimate partner violence, NPSV non-partner sexual violence.

An information specialist (AR) applied the search strategy to Medline, Embase, Psycinfo, Cochrane, Cinahl, IMEMR, Web of Science, Popline, Lilacs, WHO RHL, ClinicalTrials.gov (20 August 2018 and 3-4 December 2019) (online supplementary file 1). AR searched for grey literature via Google, Google Scholar, and websites of key organisations in the field of VAW and SRH in LMICs (UNFPA, SVRI, JPHIEGO, USAID, WHO (IRIS) SEARO, WHO (IRIS) EMRO, World Bank). AR uploaded all references into EndNote and deduplicated. Two pairs of reviewers (NVL and MM, AFO and MC) independently assessed eligibility. Disagreements were resolved through consensus or third opinion (LJB). NVL checked references and citations.

Data analysis

NVL adapted the Cochrane Effective Practice and Organisation of Care (EPOC) data extraction form.[20] We reviewed multiple publications from the same study and extracted data from the most detailed reports. The included studies were divided among reviewers who worked in pairs; one to extract data and another to check. The pairs reconciled data extraction through discussion. We extracted study details on setting, study design and aim, sample size, participants characteristics, intervention characteristics and theories, and outcomes relevant to our review questions. For each quantitative outcome, we extracted type of measure and effect estimates as reported in the primary study. If authors did not report intervention effects, we extracted the post-intervention point estimate. If a follow-up measure was reported repeatedly, we extracted the latest. We used authors' interpretation of their findings based on statistical significance or 95% confidence intervals (CI) and categorised reported effect estimates as *improvement, mixed effect,* or *null effect*. We ascribed *'mixed effect'* when one or more, but not all measures of the same outcome changed under the same intervention (e.g., reduction in physical and sexual but not psychological IPV, improvement in some coping behaviours but not in others). NVL asked corresponding authors to check data extraction forms for their studies and provide missing information; nine responded.

Reviewers assessed quality of the primary studies as part of data extraction. For randomised controlled trials (RCTs), we used the Revised Cochrane risk-of-bias tool for randomized trials (RoB 2).[21] For quasi-experimental studies we adapted the criteria listed by the EPOC Group.[22] For qualitative studies we adapted the Critical Appraisal Skills Programme (CASP) Qualitative Checklist.[23] We did not exclude studies based on their methodological quality.

We summarised the interventions by mapping onto the Health Systems Wheel[6 24] and models of health system responses to VAW.[25] It was not possible to conduct a meta-analysis of quantitative outcomes due to the heterogeneity of the interventions, the outcomes, and their measurement. We undertook a

For peer review only

narrative quantitative synthesis[26] and thematic qualitative synthesis,[27] summarised quantitative and qualitative findings in tables, and mapped the two syntheses onto a process-oriented logic model.[14] Reviewers (NVL, MC, LJB) drafted the logic model by mapping primary and secondary outcomes in the hypothesised logical order of occurrence and refined it through three iterative cycles of revisions:

1. Direct effects result from intervention activities producing structural changes at service level, cognitive and affective changes among HCPs and women increasing their readiness to address VAW.
2. Intermediate effects result from direct effects producing changes in individual's behaviour. HCPs identify women affected by VAW and provide first-line support; women disclose VAW, use offered support, develop adaptive coping strategies.
3. Health outcomes result from intermediate effects producing changes in patient health and safety. Women use adaptive coping strategies and safety behaviours; these lead to reduction in re-exposure to VAW and better health.

PATIENT AND PUBLIC INVOLVEMENT

No patients or members of the public were involved in this study.

RESULTS

Searches identified 6082 citations, we assessed 313 full text papers and included 32 reports of 26 studies (Figure 1).

Characteristics of included studies

Of the 26 studies, 18 were from sub-Saharan Africa,[28-45] three from the Middle East,[46-48] three from South Asia,[49-51] and two from South America.[52 53] Twelve quantitative evaluations were randomised controlled trials (RCTs),[30 32 33 37 39 40 45-48 51 53] six were uncontrolled before-after (UBA) studies,[36 42 49 50 52 54] six cross sectional studies,[29 35 38 41 43 44] and one was a controlled before-after evaluation (CBA).[28] Nine qualitative studies were components of mixed-methods evaluations: three embedded in RCTs,[33 55 56] two carried out alongside uncontrolled before-after studies,[42 52] four alongside cross-sectional studies,[35 38 43 44] and one standalone qualitative study.[31] No two studies of similar design evaluated the same intervention and outcomes. The duration of follow up period ranged from two weeks[31] to 4 years and 7 months.[45]

Most interventions took place in antenatal care (ANC) services (n=11),[32 35 37 43 46-51 53] followed by HIV testing and treatment (n=8),[30 31 33 36 39 40 44 45] services for survivors of sexual violence (n=5),[28 29 41 42 54] and family planning (n=2).[38 52] The SRH services were provided in primary care (n=14),[30-32 36-38 40 41 43 45 47 48 50 52] hospital (n=8),[28 33 35 44 46 49 53 54] and across both (n=3).[29 39 42] Included studies used different definitions and measures of VAW. A majority (n=11) targeted IPV.[30 31 33 35 36 38 44 45 47 50 53] Six interventions targeted sexual violence by intimate partners and non-partners.[28 29 40-42 54] Five

interventions focused on domestic violence (DV) from any family member [32 46 48 49 51] and four targeted VAW from intimate partners and non-partners.[37 39 43 52] In studies that reported sample size, 901 HCPs received VAW interventions with the average sample size of 100 ranging from 4[38] to 408.[50] A total of 12,078 women of reproductive age received VAW interventions, with the average sample size of 549 ranging from 32[40] to 2,081.[29]

Quality appraisal

Most quantitative studies were at high risk of bias (online supplementary file 2). Of 12 RCTs, seven had high risk of bias from deviations in intervention adherence,[30 33 37 39 45 46 48] seven had high risk from measurement of outcomes[33 37 39 40 45 46 53] and seven had concerns from selective reporting of outcomes.[33 37 40 45 47 48 53] Of 13 non-randomised studies (all at high risk of bias), only four adequately addressed missing outcome data.[28 38 42 49] Of ten qualitative studies, five scored 15 and above on the 20-point CASP checklist,[35 38 43 55 56] indicating relatively high quality of research design and conduct. The main weaknesses were insufficient justification of methods, reporting of recruitment and strategies for neutrality.

Types of interventions

All interventions were complex healthcare interventions,[16] however only two[39 52] included components across all domains on the Health Systems Wheel[6] (online supplementary file 3) and only four were theoretically informed.[30 32 45 51] Most VAW work was delivered by a single HCP (n=10) or by several HCPs within the same facility (n=9). Only six interventions were integrated at a systems-level[25] with HCPs identifying VAW cases, providing clinical care and first-line support, and referring to external VAW services.[31 35 36 38 43] We clustered 26 interventions into three categories based on the target group(s) and location of the common activities:

1. *Response to VAW during routine SRH consultation* (n=10). These interventions aimed to strengthen service capacity through integrating VAW identification and first-line support into routine SRH consultations.[28 30 31 33 35 36 42 44 48 50] This comprised training for HCPs in VAW screening, basic psychosocial counselling, and linkage to VAW resources. Training aimed to improve HCP knowledge, attitudes, and practices on VAW. Identification and response by trained HCPs aimed to increase women's capacity to address VAW. Duration of the integrated SRH-VAW consultation ranged between 7[30] and 30 minutes.[33 36]
2. *Response to VAW during routine SRH consultation plus community engagement* (n=9). These interventions aimed to strengthen the capacity across SRH service and surrounding communities.[29 32 38 39 41 43 45 52 54] Service-based activities were similar to the first category. The community-based activities aimed to shift gender norms and improve access to SRH-VAW services through raising awareness about post-rape care,[29 41 54] education on gender and VAW,[38 39]

For peer review only

[41 43 45 52] and couples' education about VAW.[32 39] Integrated SRH-VAW consultations supported by community engagement aimed to strengthened women's capacity to address VAW.

3. *Response to VAW in addition to routine SRH consultation* (n=7). These interventions aimed to strengthen women's capacity to cope with VAW.[37 40 46 47 49 51 53] Study personnel screened women attending routine SRH services and delivered the interventions to self-selected women with experience of VAW. This comprised more intensive support through specialist psychosocial counselling,[37 49 51 53] coping skills training[40 47] and psychoeducation.[46] The average number of sessions was three (range 1 to 7) with each session lasting from 30[37 49 53] to 90 minutes.[40 46 47] Interventions were delivered face-to-face individually,[37 46 49 51 53] in a group,[47] and mixed format[40] (Table 2).

For peer review only

Tab 2. Characteristics of included studies by intervention category and level of evidence

Study, publication	Country	Setting	Sample characteristics	Intervention (n) vs comparison (n)	Design/	Follow up	Primary outcomes of interest for this review	Secondary outcomes of interest for this review
**Response to VAW during routine SRH consultation (n=10)**								
Vakily 2017[48]	Iran	Antenatal clinic, 32 outpatient health centres	HCPs (midwives)	2-hour HCP training computer assisted (35) vs face-to-face (35)	RCT	2 months		Knowledge and attitudes about DV
Brown 2018[30]	South Africa	HIV testing and counselling, community, NGO	HIV positive women 18+	7-minute integrated HIV-IPV consultation over phone (166) vs standard care (83)	RCT	1 month	IPV upon partner notification of serostatus, harm	Perceived safety, safety behaviours, access to HIV treatment
*Haberland 2016[33]	Kenya	HIV testing in antenatal clinic, hospital with GBV centre	HCPs (HIV testing counsellors) Pregnant women 15-49	HCP training and ongoing support, 29-minute integrated HIV-IPV consultation, referral to IPV counsellor in ANC clinic (337) vs standard care (351)	RCT Nested mixed-method process evaluation	1 month	Any IPV, harm	IPV screening, referrals Women's knowledge, attitudes, self-esteem, perceived intervention effect, HIV care Intervention acceptability
Abeid 2016[28]	Tanzania	Post-rape care service, 5 health centres and referral hospitals	HCPs (doctors, nurses, assistant medical/clinical officers)	5-day training, guidelines, infrastructure improvement (100) vs minimal intervention (53)	Controlled before-after	12 months		Knowledge and attitudes about sexual violence and post-rape care Provision of post-rape care
Jayatilleke 2015[50]	Sri Lanka	Antenatal clinic, community	HCPs (midwives)	4-day training, handbook, external referral (408)	Uncontrolled before-after	6 months		Knowledge, practices, responsibility, readiness for identifying and responding to IPV, provision of referrals
Matseke 2013[36]	South Africa	HIV testing and counselling in antenatal clinic, 16 primary health care clinics	Pregnant women 18+	HPC training, 30-minute integrated ANC-IPV consultation, external referral (160)	Uncontrolled before-after	3 months	Perceived risk of becoming a victim of femicide	
Smith 2013[42]	Kenya, Ethiopia, Jordan, Democratic Republic of Congo	Post-rape care service, 35 humanitarian settings, NGO	HCPs (doctors, nurses, midwives)	4-day training, infrastructure improvement (106)	Uncontrolled before-after Qualitative study	3 months		Attitudes, knowledge, skills on sexual violence and post-rape care, provision of post-rape care
Laisser 2011[35]	Tanzania	Antenatal clinic, hospital	HCPs (clinical/medical	HCP training (39), infrastructure improvement, integrated ANC-IPV	Cross sectional	3 weeks		Intervention acceptability

For peer review only

Study, publication	Country	Setting	Sample characteristics	Intervention (n) vs comparison (n)	Design/	Follow up	Primary outcomes of interest for this review	Secondary outcomes of interest for this review
			officers, nursing officers) Women 18+	consultation (102), external referral	Qualitative study			
Undie 2016[44]	Kenya	HIV testing in antenatal clinic, hospital with GBV centre	Women	HCP training, integrated HIV-IPV consultation (1210), assisted referral to on-site GBV centre	Cross sectional Qualitative study	7 months		IPV screening, referrals Intervention acceptability
Cristofides 2010[31]	South Africa	HIV testing and counselling, primary health care clinic	HCPs (lay counsellors) Women	HCP training (16), integrated HIV-IPV consultation (35), external referral	Qualitative study	2 weeks		Intervention acceptability
**Response to VAW during routine SRH consultation plus community engagement (n=9)**								
Cockcroft 2019[32]	Nigeria	Universal home visits, 4 communities	Pregnant women 14-49	HCP training, infrastructure improvement, integrated DV-universal home visits that discussed domestic violence, heavy work in pregnancy, ignorance of danger signs, and lack of spousal communication with pregnant women (1837) and their partners vs delayed intervention (1853)	Cluster RCT	12 months	Physical DV, pregnancy delivery, postnatal complications	Use of SRH services
Settergren 2018[39]	Tanzania	HIV/AIDS services, hospital and health centre	Women 15-49	Systems level activities, HCP training, infrastructure improvement, integrated-HIV-GBV consultation, onsite and external referral, community and couple education (6 facilities, 656 women) vs standard care (6 facilities, 643 women)	Cluster RCT	28 months	Any IPV	Provision of services to IPV positive patients
Wagman 2015[45]	Uganda	HIV testing and counselling, community	Women 15-49	HCP training, integrated HIV-IPV consultation, onsite referral (6 facilities, 1812 women) vs standard care (5 facilities, 2127 women)	Cluster RCT	4 years and 7 months	Physical, emotional, sexual IPV, HIV incidence	Risk behaviours and HIV disclosure
*Bott 2014[52 57 58]	Dominican Republic, Peru, Venezuela	3 family planning clinics, NGO	HCP (doctors, nurses, midwives, counsellors, social workers, psychologists, receptionists) Women 12+	Systems level activities, HCP training and ongoing support, infrastructure improvement, integrated GBV-family planning consultation, referral to onsite GBV specialist (4 clinics)	Uncontrolled before-after Qualitative study	3 years		HCPs attitudes, knowledge, readiness for identifying and responding to GBV Intervention acceptability

For peer review only

Study, publication	Country	Setting	Sample characteristics	Intervention (n) vs comparison (n)	Design/	Follow up	Primary outcomes of interest for this review	Secondary outcomes of interest for this review
Kim 2007[34 54]	South Africa	Post-rape care service, hospital	Survivors of sexual violence	Systems level activities, 2-day HCP training (334), infrastructure improvement, community education on post-rape care	Uncontrolled before-after	No information		Use, quality, and cost of post-rape care service
Bress 2018[29]	Democratic Republic of Congo	Post-rape care service, 12 primary care clinics and referral hospital	Survivors of sexual violence 12+	HCP training and ongoing support, infrastructure improvement, community education on post-rape care (13 sites, 2081 survivors)	Cross-sectional	4 years		Provision of post-rape kit
Samandari 2016[38 59]	Guinea	Family planning clinic	HCPs (nurse, midwife, counsellor, support/admin staff) Women	System level activities, 7-day HCP training and ongoing support (4), integrated family planning-IPV consultation (171), external referral, community education	Cross-sectional Qualitative study	4 months		IPV identification, safety planning, referrals Intervention acceptability
Sithole 2018[41]	Zimbabwe	Comprehensive post-rape care service, 8 policlinics, NGO	HCPs (doctors, nurses, managers) Survivors of sexual violence	HCP training (80), infrastructure improvement, post-rape care (1669), community education on post-rape care	Cross sectional service evaluation	4 years		HCPs knowledge about post-rape care Provision of post-rape care
Turan 2013[43]	Kenya	Antenatal clinic, primary health care clinic	HCPs (all clinic staff including admin, community volunteers, lay health workers) Pregnant women	40-hour HCP training, integrated ANC-GBV consultation (134), assisted external referral, community education	Cross sectional Qualitative study	5 months		GBV identification, referrals Intervention acceptability
**Response to VAW in addition to routine SRH consultation (n=7)**								
Cripe 2010[53]	Peru	Antenatal clinic, hospital	Pregnant women 18-45 with IPV experience	1* 30-minute psychosocial counselling session by social worker, resource card, external referral (110) vs minimal intervention (110)	RCT	Prenatal appointment to 1 week after delivery	Quality of life	Safety behaviours, use of community resources
Khalili 2019[46]	Iran	Antenatal clinic, University health centers	Pregnant women 20+ with IPV experience	4*90-minute psychoeducational sessions by counsellor (50) vs standard care (50)	RCT	2 months	Verbal and physical IPV, psychological distress	
Mutisya 2018[37]	Kenya	Antenatal clinic, 12 primary health care clinics	Pregnant women 18-45 with IPV experience	1-3*30-35-minute psychosocial counselling sessions by researcher, risk assessment, safety planning, resource card, external referral	RCT	6 months	Physical, emotional, severe combined IPV and harassment, depression	

For peer review only

Study, publication	Country	Setting	Sample characteristics	Intervention (n) vs comparison (n)	Design/	Follow up	Primary outcomes of interest for this review	Secondary outcomes of interest for this review
				(141) vs minimal intervention (142)				
Sapkota 2020[51 56]	Nepal	Antenatal clinic, hospital	Pregnant women 18+ with DV experience	1*35-45-minute psychosocial session by counsellor, resource card, contact details of the counsellor (70) vs minimal intervention (70)	RCT Nested qualitative study	Prenatal appointment to 6 weeks after delivery	Depression, anxiety, quality of life	Self-efficacy, safety behaviours, social support Intervention acceptability
Sikkema 2018[40 55]	South Africa	HIV testing and treatment, primary health care clinic	HIV positive women 18+ with experience of sexual violence	4 individual and 3 group*90-minute psychosocial training sessions by trained lay provider (32) vs standard care (32)	RCT Nested qualitative study	6 months	PTSD	Coping strategies, engagement with HIV treatment Intervention acceptability
Tanghizaden 2018[47]	Iran	Antenatal clinic, 16 health centres	Pregnant women with IPV experience	4*90-minute psychosocial training sessions on problem-solving skills by researcher (125) vs standard care (132)	RCT	3 months	Physical, psychological, sexual IPV	
Arora 2019[49]	India	Antenatal clinic, 2 hospitals	Pregnant women with DV experience	≥2*30-45-minute psychosocial sessions by counsellor (155)	Uncontrolled before-after	First prenatal appointment to 6 weeks after delivery	Physical, emotional, financial DV, physical health, emotional health	Knowledge and attitudes about DV, coping behaviours

Note. * grey literature. NGO non-governmental (third sector) organisation. RCT randomised controlled trial. HCP health care providers. IPV intimate partner violence. DV domestic violence. GBV gender-based violence. HIV human immunodeficiency viruses. PTSD posttraumatic stress disorder. SRH sexual and reproductive

For peer review only

Interventions effects and outcomes

The logic model displays all outcomes of interest in the three intervention categories. The arrows illustrate the hypothesised flow of change from intervention activities through direct and intermediate effects to health outcomes (Figure 2). None of the included studies reported outcomes at service level. Most interventions that strengthen capacity of SRH service and community measured direct and intermediate effects on HCP and women's knowledge, attitudes, and behaviour. In contrast, all interventions that strengthen capacity of women reported their health-related outcomes, but only a few looked at preceding direct and intermediate effects (Figure 2, Table 3).

Table 3. Effects and outcomes in randomised and non-randomised evaluations of interventions addressing VAW in SRH services

	Intervention category	Improvement		Mixed effect		Null effect		Studies, n
		RCT	Non-randomised	RCT	Non-randomised	RCT	Non-randomised	
	**Response to VAW during routine SRH consultation (n=10)**							
Direct effects	HCP knowledge	**Vakily 2017**[48]	Jayatilleke 2015,[50] Smith 2013,[42] Abeid 2016[28]					4
Direct effects	HCP attitudes		Smith 2013[42]				**Vakily 2017**,[48] Abeid 2016[28]	3
Direct effects	HCP readiness		Jayatilleke 2015,[50] Smith 2013[42]					2
Direct effects	Women's knowledge	Haberland 2016[33]						1
Direct effects	Women's attitude					Haberland 2013[33]		1
Direct effects	Women's readiness					Haberland 2013[33]		1
Intermediate effects	HCP behaviour		Jayatilleke 2015[50]	Haberland 2013[33]	Smith 2013,[42] Abeid 2016[28]			4
Intermediate effects	Women's behaviour			Haberland 2016[33]		Brown 2018[30]		2
Outcomes	Re-exposure to VAW	Brown 2018[30]	Matseke[36]			Haberland 2016[33]		3
Outcomes	Harm					Brown 2018,[30] Haberland[33]		2
	**Response to VAW during SRH consultation plus community engagement**							
Direct effects	HCP attitudes		Bott 2004[52]					1
Direct effects	HCP readiness				Bott 2004[52]			1
Direct effects	Women attitude			Settergren 2018[39]				1
Intermediate effects	HCP behaviour					Settergren 2018[39]		1
Intermediate effects	Women behaviour		Kim 2007[54]	Settergren 2018[39]		**Cockcroft 2019**,[32] Wagman 2015[44]		4
Outcomes	Re-exposure to VAW	**Cockcroft 2019**[32]		Wagman 2015[45]		Settergren 2018[39]		3
Outcomes	Sexual and reproductive health			**Cockcroft 2019**[32]		Wagman 2015[45]		2
	**Response to VAW in addition to routine SRH consultation**							

	Intervention category	Improvement		Mixed effect		Null effect		Studies, n
		RCT	Non-randomised	RCT	Non-randomised	RCT	Non-randomised	
Intermediate effects	Women behaviour	**Sapkota 2020[51]**		Sikkema 2018[40]		Cripe 2010[53]	Arora 2019[49]	4
Outcomes	Re-exposure to VAW	**Khalili 2019,[46] Mutisya 2018[37]**	Arora 2019[49]	Tanghizaden 2018[47]				4
	Sexual and reproductive health					Sikkema 2018[40]		1
	Physical health		Arora 2019[49]					1
	Mental health	**Khalili 2019,[53] Mutisya 2018,[37] Sapkota 2020[51]**	Arora 2019[49]			Cripe 2010,[53] Sikkema 2018[40]		6
	Quality of life	**Sapkota 2020[51]**				Cripe 2010[53]		1
	Studies, n	7	7	6	2	7	3	

Note. HCP health-care providers, VAW violence against women, SRH sexual and reproductive health., RCT randomised controlled trial. Bold indicates studies that reported sample size calculation.

Direct effects. The routine SRH-VAW intervention category had overall positive direct effects on HCP and women's knowledge, attitudes, and readiness. Interventions with community engagement reported mixed and improved direct effects.

Intermediate effects. Changes in professional behaviour were measured through the rates of VAW screening, provision of referrals to support services, and post-rape care. Changes in women's behaviour were measured through VAW disclosure, uptake of referrals and other services. The overall evidence was uncertain across all three intervention categories with RCTs and non-randomised evaluations reporting improved, mixed, and null effects.

Health related outcomes. Only half studies reported measures of health and re-exposure to VAW, i.e. 13 interventions did not escalate VAW, were safe to women, and led to some health improvement. The overall direction of effect was towards improvement in the *routine SRH-VAW consultation* category and in the *additional response* category. In contrast, most interventions in the *plus community engagement* category reported mixed or null effect on women's health and re-exposure to VAW.

Of 26 studies, only two reported changes across all three domains of the logic model, one from the *routine SRH-VAW consultation* category[33] and one from the *plus community engagement* category.[39] Four evaluations of the *additional response* category reported changes across two domains - intermediate effects and women's outcomes.[40 49 51 53] These six studies were consistent with our hypotheses. If intervention improved women's safety behaviour and use of support services, their health improved.[51] Mixed or null effect on HCP and women's cognitive and behavioural outcomes

For peer review only

suggested some explanation for no change in re-exposure to VAW.[33 39] Contradicting direct and indirect effects and outcomes [40 49]indicated barriers on the pathway from intervention to outcomes.

Response to VAW during routine SRH consultation

Of ten evaluations, two RCTs[30 33] and one UBA[36] studies reported conflicting findings on re-exposure to VAW; none measured women's health (Figure 2, Supplementary file 4). These three interventions did not lead to escalation of violence. There was some evidence for the reduction in HIV-disclosure-related violence at up to 2-month follow-up[30] and risk of becoming a victim of femicide at 3-month follow-up[36] possibly through some improvement in HCP's and women's cognition and practice. Two RCTs reported that integrated HIV-IPV consultation caused no harm to women.[30 33] However, all studies were at high risk of bias.

An RCT and UBA in the Kenyan hospital with on-site GBV centre reported converging findings. The UBA study of an integrated HIV-IPV consultation with assisted referral to GBV centre reported 8% IPV disclosure rate, 75% referrals provision and 40% uptake.[44] The RCT of an integrated HIV-IPV consultation with referral to in-service GBV specialist found increased rates of IPV screening but no effect on provision of referrals. The 29-minute integrated HIV-IPV consultation increased women's knowledge about VAW and IPV disclosure, but had no effect on their attitudes, readiness to address VAW, uptake of referrals and re-exposure to IPV.[33]

Another RCT of a 7-minute integrated HIV-IPV consultation over the phone found that 62% of women used a safety plan and 80% employed at least one safety strategy, however their use of SRH services and perceived risk and safety did not change. Despite no effect on women's behaviour, the trial reported a four-fold reduction in HIV-disclosure-related violence (OR 4.37; 95% CI 1.46-13.44).[30] One UBA found that a 30-minute integrated HIV-IPV consultation contributed towards a reduction in the risk of femicide (mean difference 3.2, SD 3.56; 95% CI 2.43-3.98).[36]

Other randomised and non-randomised evaluations of varied one-off training for HCPs reported conflicting findings on their knowledge, attitudes, and readiness to address VAW. Non-randomised studies reported increased IPV screening rates,[50] mixed effect on provision of post-rape care,[28 42] and low VAW disclosure rates.[35]

Qualitative evaluations confirmed that training increased HCP capacity to respond to VAW during routine SRH consultations.[33 35 42 44] One evaluation of HCP training on post-rape care described a potential mechanism of impact on HCP negative attitudes by separating personal beliefs about victims from the provision of clinical care.[42] Women found that HIV-IPV consultation improved their knowledge about IPV. They benefited from emotional support and felt empowered.[31 33 44]

Response to VAW during routine SRH consultation plus community engagement

For peer review only - http://bmjopen.bmj.com/site/about/guidelines.xhtml

Three cluster RCTs reported conflicting findings on women's SRH and re-exposure to VAW. The overall effect was uncertain (Figure 2, Supplementary file 5).[32 39 45] A Nigerian RCT at low risk of bias evaluated universal home visits that discussed DV and other risk factors with pregnant women and their spouses. The trial reported no effect on women's use of SRH services, reduction in the proportion who experienced physical DV (RD 0.064 (95% CI 0.045-0.084), and mixed effect on pregnancy and birth indicators.[32] A Tanzanian RCT at high risk of bias evaluated integrated HIV-VAW consultation, onsite and external referrals, community and couple education. Intervention had a mixed effect on women's attitudes about VAW and gender roles, no effect on rates of enquiry and referrals, mixed effect on women's use of SRH services, and null effect on re-exposure to IPV (OR = 0.85, 95% CI 0.62-1.16).[39] A Ugandan RCT at high risk of bias evaluated integrated HIV-IPV consultation, onsite referral and community education. The intervention had no effect on women's safety behaviour and null effect on SRH. Re-exposure to physical and sexual IPV reduced (relative prevalence risk ratios (PRR) of 0.74 (95% CI 0.63-0.86), 0.75 (95% CI 0.62-0.90), respectively), but psychological IPV and HIV did not change.[45]

Non-randomised studies reported more positive effects on HCP knowledge, attitudes, readiness,[52] and provision and use of post-rape care.[29 41 54] They also reported high rates of IPV screening and provision of referrals and clinical care by HCPs vs low uptake of referrals and other services by women.[38 43]

Qualitative evaluations confirmed that VAW training transformed HCP attitudes towards patients and their own work and improved their diagnostic and counselling skills. HCPs appreciated the intervention and expressed a willingness to continue VAW work.[38 43 44 52] Women felt empowered and supported by HCPs.[31 35 38 52] Community engagement raised awareness about SRH-VAW services.[43]

Response to VAW in addition to routine SRH consultation

This intervention category had the most robust evidence from six RCTs[37 40 46 47 51 53] and one UBA evaluation[49] (Figure 2, Supplementary file 6). The studies reported conflicting results with more evidence for a reduction in re-exposure to VAW at up to 6-month post-intervention and improvement in health possibly through improvement in women's safety behaviour. There was some evidence that longer interventions produced better outcomes.[37 46 49]

Psychosocial counselling. Three RCTs of counselling sessions for pregnant women with experience of VAW reported conflicting results with no evidence for a dose-effect. The overall effect was towards reduction in re-exposure to violence and improvement in health outcomes.[37 51 53] An adequately powered Nepalese RCT with low risk of bias evaluated a 35-45-minute psychosocial counselling session with a resource card and counsellor's contact details. The trial reported positive effects on women's self-efficacy (MD 0.5; 95% CI 0.30-0.72), perceived social support (MD 0.73; 95% CI 0.39-1.06), safety behaviours (MD 2.41; 95% CI 1.43-3.40), anxiety (MD -3.73; 95% CI -

For peer review only

5.42 to -2.04), depression (MD -3.41; 95% CI -4.84 to -1.99), and quality of life (MD 2.45; 95% CI 1.51-3.39).[51] The embedded qualitative study confirmed that women felt empowered, supported, and valued by the counsellor.[56] In contrast, a Peruvian RCT with some bias concerns of a 30-minute counselling session with a resource card and external referral had no effect on women's safety behaviours, health, use of community resources.[53] A Kenyan RCT of up to three 30-35-minute counselling sessions with resource card, safety planning and external referral reduced depression (MD = 7.12; 95% CI 6.21-8.03) and re-exposure to IPV (MD = 13.51; 95% CI 9.99-17.02).[37] Similarly, an Indian UBA evaluation of two or more 30-45-minute psychosocial counselling sessions found that most women increased awareness of and readiness to address VAW. Physical violence and health problems decreased.[49]

Coping skills training. Two RCTs with high risk of bias evaluated more intensive training interventions and found mixed effects on behaviour and mixed and null effects on VAW and health.[40 47] An Iranian RCT of four 90-minute group sessions found a reduction in re-exposure to physical IPV (RR 0.78; 95% CI 0.63-0.83) and psychological IPV (RR 0.73; 95% CI 0.64-0.83), but null effect on sexual IPV (RR 0.87; 95% CI 0.69-1.09).[47] A South African RCT of seven 90-minute sessions reported null effect on coping behaviour, use of SRH services, PTSD symptoms and HIV viral load among HIV positive women with a history of sexual violence.[40] However, the embedded qualitative study found that training increased women's knowledge about VAW impact and improved their self-esteem, coping and communication skills.[55]

Psychoeducation. An Iranian RCT with high risk of bias of four 90-minute sessions with pregnant women found reduction in scores of IPV and psychological distress.[46]

Cost effectiveness outcomes

No studies evaluated cost effectiveness of VAW interventions in SRH services. One study of an integrated HIV-IPV consultation paid HCPs $6 per day for identifying VAW patients and referring to the on-site GBV clinic.[44] One evaluation of post-rape service improvement with community engagement reported resource costs.[54] Seven studies across all three intervention categories mentioned intervention costs but did not report actual data.[33 37 41 43 44 51 52]

Barriers

Supplementary file 7 summarises factors which acted as barriers on the pathway from intervention to outcomes. We developed three analytical themes cross-cutting through individual, community, and system levels.

Acceptability of VAW. Four evaluations of interventions on *response during routine SRH consultation* and *response with community engagement* described attitudes accepting violence and patriarchal gender norms as major barrier to behaviour change.[31 35 42 43]

For peer review only

Fear of negative consequences. Eight studies across all three interventions categories identified fear of negative consequences as a barrier to identification, disclosure, and engagement in VAW interventions.[31 33 35 38 41 52 55 56]

Limited readiness. Evaluations reported limited readiness for engaging in VAW at system and individual levels. In evaluations of *response to VAW during routine SRH consultation*[28 31 33 35 44] and *response with community engagement*,[38 52] HCPs consistently mentioned chronic problems with staffing, inadequate funding, no private space, lack of support from leadership, and high demand for basic SRH services without the additional VAW work. Readiness gaps at system level included the lack of services to refer to, poor referral systems and untrained staff in other agencies. Screening identified many IPV-positive women and specialist services could not address the increased demand.[31 35 43 52] Across all intervention categories, HCPs and women described barriers at societal level that prevented women from accessing SRH services, using referrals, and participating in psychosocial interventions. Work-related conflicts, no money for transport, and financial dependence on husband were mentioned most frequently.[33 35 41 55] Finally, two evaluations of *response to VAW during routine SRH consultation* explored reasons for low uptake of referrals to specialist services. Some women had expectations that could not be met by the current services. Instead of referral, they wanted HCPs to talk to their partners about stopping the abuse.[31] Some women wanted to receive all SRH and VAW services on the same day.[33 44]

DISCUSSION

We conducted a mixed-methods systematic review of literature from LMICs on the effectiveness and barriers to SRH service interventions to improve the response to VAW. We grouped 26 heterogeneous complex interventions into three categories: (i) response to VAW during routine SRH consultation, (ii) response to VAW during routine SRH consultation plus community engagement, and (iii) response to VAW in addition to routine SRH consultation. We mapped outcomes on a process-oriented logic model illustrating the hypothesised changes from intervention through direct and intermediate effects to outcomes. We cannot conclude which intervention was the most effective due to heterogeneity of the interventions and outcomes measures at varying time points. Overall, these interventions did not escalate violence and were safe for women. We found mixed effects on women's health and re-occurrence of VAW across all three categories, with studies reporting conflicting findings. Evaluations of the varied *responses to VAW during routine SRH consultation* found reduction in HIV-disclosure-related IPV and potential risk of becoming a victim of femicide, but no effect on IPV in the past month. Some of these effects could be attributed to improvement in HCPs' readiness, screening, and provision of first-line support for VAW. For women, these effects could be attributed to increased knowledge about VAW and disclosure of violence. *Response to VAW during routine SRH consultation plus community engagement* had uncertain evidence with single studies reporting improvement, mixed effect, and no effect on re-exposure to violence and SRH possibly

For peer review only - http://bmjopen.bmj.com/site/about/guidelines.xhtml

through some improvement in provision and use of SRH services. More intensive psychosocial interventions delivered to women with experience of VAW *in addition to routine SRH consultation* had the most robust evidence for reduction in re-occurrence of violence and improvement in health outcomes possibly through an improvement in safety behaviours. We identified individual, community, and system-level barriers that could disrupt the pathway from intervention to outcomes: (i) attitudes and social norms that accept and normalise violence, (ii) fear of negative consequences and (iii) limited readiness of individuals, healthcare and other systems and society to address VAW. No studies reported cost-effectiveness analysis.

An important finding is that response to VAW in SRH services did not escalate violence. Our synthesis suggests that interventions that strengthened capacity of HIV and ANC services to respond to VAW can increase identification and provision of first-line support to women experiencing violence which can lead to reduction in HIV-disclosure-related IPV, physical and sexual IPV, and the risk of becoming a victim of femicide. More intensive psychosocial interventions that strengthened women's capacity to address VAW can increase use of SRH services and safety behaviours, reduce re-exposure to IPV and DV, and improve health and quality of life. This effect can be explained by better theoretical development of the additional psychosocial interventions, higher dose of provider-patient contact, delivery by study personnel specialised in counselling and VAW, and samples of self-selected women who could be more motivated and ready for change. The first two intervention categories might appear less effective because few studies examined women's outcomes beyond the point of identification and first-line response. Our findings suggest that future VAW interventions should strengthen the capacity across individual HCPs and women, SRH services, and communities.

The uncertain evidence for the two SRH consultation-based intervention categories is consistent with other evidence for a healthcare response to VAW in LMICs[9 60] and to VAW among pregnant women.[10 61 62] The evidence for the effectiveness of longer psychosocial interventions as an addition to routine SRH consultations is in line with a recent meta-analysis which found that psychosocial interventions in healthcare settings and communities in LMICs led to a 25-27% reduction in IPV.[63]

An important finding on direct effects of interventions is that increasing awareness about VAW and relevant procedures often did not lead to a shift in judgemental attitudes towards victims, the major barrier to changes in professional and patient behaviour. An exception were studies in the *plus community engagement category*, one reporting improvement in HCP attitudes and one partial shift in women's attitudes and gender norms. These findings can be explained by the community and societal roots of gender norms and attitudes of HCPs and women which are best addressed at community and societal levels. Our findings suggest that a shift in individual's attitudes potentially leading to behavioural change can be achieved through service-based plus community-based education. This

For peer review only

finding is consistent with a review identifying evidence for community mobilisation reducing VAW in LMICs.[60]

An important finding is that routine integrated SRH-VAW consultations with referral or signposting to VAW/GBV specialist or other services did not increase women's use of these services. This gap between availability and acceptability of referrals to other services has several explanations. HCP response may not have matched women's needs and expectations, or the VAW services were not accessible, or contextual factors prevented women from accessing them. This finding suggests that integrated SRH-VAW consultation and VAW services require better tailoring to women's needs and expectations. This should be based on understanding what women want and need and what is feasible. A recent qualitative meta-synthesis found that after disclosing IPV to HCPs, women wanted assistance with documentation of injuries, insurance issues and help with connecting to community services more than referrals to IPV services.[64] The feasibility and acceptability of HCPs engaging with men who use violence needs further exploration in LMICs.

Finally, most interventions in the first two categories targeted the behaviour of individual HCPs rather than the SRH service or healthcare system. The expectation was that trained HCPs would integrate VAW work into their clinical practice without structural changes to the environment, support from leadership, supervision, monitoring and incentivisation. Most qualitative evaluations described passionate HCPs who were enthusiastic about helping patients experiencing violence. However, some studies reported HCPs concerns about unrealistic expectations and limited health system readiness for embedding VAW work in routine practice. This finding is in line with other studies on health systems readiness for responding to VAW.[7 8] The obstacles to integrating a VAW response in SRH services overlap with those reported in the systematic review of barriers and facilitators to integrating health services responses to IPV in LMICs.[65]

We need more methodologically robust evaluations of interventions for strengthening the capacity of communities, healthcare systems, SRH services, and individual HCPs and women with measures throughout the pathway from intervention to women's outcomes. Evidence on cost-effectiveness of VAW interventions in SRH services is another gap. Finally, very few interventions have been evaluated in LMICs outside Africa.

Strengths and limitations

This review is methodologically strong. It involved a multidisciplinary team of researchers from LMICs and the UK with content and methodological expertise in healthcare system responses to VAW and global health. We followed the Cochrane method and included studies of any design reported in peer-reviewed and grey literature in any language with English abstract. This comprehensive approach ensured inclusion of the most relevant studies from the field and reduced the potential for bias/errors.

For peer review only

This review's results are relevant for practitioners and policy makers in LMICs. The logic model approach allowed to: (i) illustrate the hypothesised cause-result pathway, (ii) map evidence from primary studies for the direct and intermediate effects and outcomes, (iii) identify barriers that can disrupt the trajectory of changes. It allowed us to present evidence in a format understandable to the end users – people who develop, deliver, evaluate and fund VAW interventions in LMICs.

The evidence for VAW interventions in SRH settings is weak because of the methodological limitations of the primary studies and uncertain effectiveness of the interventions. Each study used differing operational definitions and outcomes measures, relied on self-report, and evaluated a different complex intervention. No studies measured and adjusted analysis for organisation or system level factors which could impact intervention effect on outcomes of individual HCPs and women, although those were explored in some qualitative evaluations. Because of the diverse complex interventions and outcomes measures we could not perform a meta-analysis. Our findings should be interpreted with caveats because 2/3 of trials and all 13 quasi-experimental studies had high risk of bias.

CONCLUSION

We found that interventions to improve response to VAW in SRH services did not cause harm to women. Some interventions that strengthened capacity of HIV and ANC services increased identification and reduced some types of IPV. Some interventions that strengthened capacity of HIV and ANC services and communities improved use of SRH services and reduced re-exposure to some types of VAW. Several studies identified a gap between provision and uptake of referrals to VAW services suggesting that the first-line support should be better tailored to women's needs and preferences. Most additional psychosocial interventions that strengthened women's capacity reduced re-exposure to violence and improved health. Our findings are relevant to people who develop, implement, evaluate, and fund VAW interventions in healthcare. Future interventions should have better theoretical development and use a systemic approach to strengthen the capacity to respond to VAW across the healthcare system, SRH services, communities, and HCPs and women. Future evaluations of VAW interventions in SRH services in LMICs should have longer follow-up and use standardised measures of individual-, organisation-, and system- level outcomes on the pathway from intervention to women's health.

CONTRIBUTORS

GF, LJB, NVL planned the review. All co-authors contributed to the protocol development. AR constructed and ran searchers. NVL and MM screened titles and abstracts. NVL, MM, LJB, MC, AFO screened full texts. NVL, MM, MC, AFO, SP, SS, TR, AS, PR, AA, AR, LJB worked in pairs on data extraction, risk assessment. NVL summarised the findings. NVL, MC, LJB developed logic model.

For peer review only

NVL wrote first draft of the manuscript. All co-authors contributed to a further two revisions and approved final manuscript.

FUNDING

This research was funded by the National Institute for Health Research (NIHR) (17/63/125) using UK aid from the UK Government to support global health research. The views expressed in this publication are those of the authors and not necessarily those of the NIHR or the UK government.

AR was funded by the NIHR Applied Research Collaboration West (NIHR ARC West). The views expressed in this article are those of the authors and not necessarily those of the NIHR or the Department of Health and Social Care.

For peer review only

DISCLAIMER

The funder of the study had no role in study design, data collection, data analysis, data interpretation, or writing of the report. The corresponding author had full access to all the data in the study and had final responsibility for the decision to submit for publication.

COMPETING INTERESTS

None declared.

ETHICS APPROVAL

Ethics approval was not required. All data used in this review were already in the public domain.

PATIENT CONSENT FOR PUBLICATION

Not required.

DATA SHARING STATEMENT

No additional data available. All data relevant to the study are included in the article and uploaded as supplementary files.

REFERENCES

1. UN General Assembly. Declaration on the Elimination of Violence against Women. https://www.refworld.org/docid/3b00f25d2c.html UN General Assembly, 20 December 1993.
2. Campbell JC. Health consequences of intimate partner violence. *Lancet* 2002;359(9314):1331-6. doi: 10.1016/S0140-6736(02)08336-8 [published Online First: 2002/04/20]
3. WHO. Violence against women prevalence estimates, 2018. Global, regional and national prevalence estimates for intimate partner violence against women and global and regional prevalence estimates for non-partner sexual violence against women. Geneva: World Health Organization, on behalf of the United Nations Inter-Agency Working Group on Violence

Against Women Estimation and Data (UNICEF, UNFPA, UNODC, UNSD, UNWomen), 2021:112.

4. Ansara DL, Hindin MJ. Formal and informal help-seeking associated with women's and men's experiences of intimate partner violence in Canada. *Soc Sci Med* 2010;70(7):1011-8. doi: 10.1016/j.socscimed.2009.12.009 [published Online First: 2010/02/04]

5. Bonomi AE, Anderson ML, Rivara FP, et al. Health care utilization and costs associated with physical and nonphysical-only intimate partner violence. *Health Serv Res* 2009;44(3):1052-67. doi: 10.1111/j.1475-6773.2009.00955.x [published Online First: 2009/08/14]

6. Garcia-Moreno C, Hegarty K, d'Oliveira AF, et al. The health-systems response to violence against women. *Lancet* 2015;385(9977):1567-79. doi: https://dx.doi.org/10.1016/S0140-6736(14)61837-7

7. Colombini M, Alkaiyat A, Shaheen A, et al. Exploring health system readiness for adopting interventions to address intimate partner violence: a case study from the occupied Palestinian Territory. *Health Policy Plan* 2020;35(3):245-56. doi: 10.1093/heapol/czz151 [published Online First: 2019/12/13]

8. d'Oliveira A, Pereira S, Bacchus LJ, et al. Are We Asking Too Much of the Health Sector? Exploring the Readiness of Brazilian Primary Healthcare to Respond to Domestic Violence Against Women. *Int J Health Policy Manag* 2020 doi: 10.34172/ijhpm.2020.237 [published Online First: 2020/12/18]

9. Kirk L, Terry S, Lokuge K, et al. Effectiveness of secondary and tertiary prevention for violence against women in low and low-middle income countries: a systematic review. *BMC Public Health* 2017;17(1):622. doi: https://dx.doi.org/10.1186/s12889-017-4502-6

10. Sapkota D, Baird K, Saito A, et al. Interventions for reducing and/or controlling domestic violence among pregnant women in low- and middle-income countries: a systematic review. *Syst Rev* 2019;8(1):79. doi: 10.1186/s13643-019-0998-4 [published Online First: 2019/04/04]

11. Higgins J, Thomas J, Chandler J, et al. Cochrane handbook for systematic reviews of interventions version 6.1 (updated September 2020). Second edition. ed. www.training.cochrane.org/handbook: Cochrane, 2020:pages cm.

12. Liberati A, Altman DG, Tetzlaff J, et al. The PRISMA statement for reporting systematic reviews and meta-analyses of studies that evaluate health care interventions: explanation and elaboration. *PLoS Med* 2009;6(7):e1000100. doi: 10.1371/journal.pmed.1000100 [published Online First: 2009/07/22]

13. Sandelowski M, Voils CI, Barroso J. Defining and Designing Mixed Research Synthesis Studies. *Res Sch* 2006;13(1):29. [published Online First: 2006/04/01]

14. Rohwer A, Pfadenhauer L, Burns J, et al. Series: Clinical Epidemiology in South Africa. Paper 3: Logic models help make sense of complexity in systematic reviews and health technology

assessments. *J Clin Epidemiol* 2017;83:37-47. doi: 10.1016/j.jclinepi.2016.06.012 [published Online First: 2016/10/30]

15. WHO. Strengthening health systems to respond to women subjected to intimate partner violence or sexual violence: a manual for health managers. Geneva, Switzerland.: World Health Organisation, 2017.
16. Campbell M, Fitzpatrick R, Haines A, et al. Framework for design and evaluation of complex interventions to improve health. *BMJ* 2000;321(7262):694-6.
17. Balfour J, Abdulcadir J, Say L, et al. Interventions for healthcare providers to improve treatment and prevention of female genital mutilation: a systematic review. *BMC Health Serv Res* 2016;16(1):409. doi: 10.1186/s12913-016-1674-1 [published Online First: 2016/08/21]
18. Fraley HE, Aronowitz T, Stoklosa HM. Systematic Review of Human Trafficking Educational Interventions for Health Care Providers. *West J Nurs Res* 2020;42(2):131-42. doi: 10.1177/0193945919837366 [published Online First: 2019/03/30]
19. Fantom N, Serajuddin U. The World Bank's classification of countries by income. Policy research working paper. http://hdl.handle.net/10986/23628: World Bank, 2016.
20. Cochrane Effective Practice and Organisation of Care (EPOC). Data collection form. EPOC Resources for review authors. epoc.cochrane.org/resources/epoc-specific-resources-review-authors 2017.
21. Higgins J, Savović J, Page M, et al. Chapter 8: Assessing risk of bias in a randomized trial. In: Higgins J, Thomas J, Chandler J, et al., eds. Cochrane Handbook for Systematic Reviews of Interventions version 61 (updated September 2020). https://training.cochrane.org/cochrane-handbook-systematic-reviews-interventions: Cochrane 2020.
22. EPOC. Suggested risk of bias criteria for EPOC reviews. Cochrane Effective Practice and Organisation of Care (EPOC) EPOC Resources for review authors. epoc.cochrane.org/resources/epoc-resources-review-authors, 2017.
23. Critical Appraisal Skills Programme. CASP Qualitative Checklist [online]. https://casp-uk.net/casp-tools-checklists/, 2018.
24. Colombini M, Mayhew SH, Ali SH, et al. An integrated health sector response to violence against women in Malaysia: lessons for supporting scale up. *BMC Public Health* 2012;12:548. doi: 10.1186/1471-2458-12-548 [published Online First: 2012/07/26]
25. Colombini M, Mayhew S, Watts C. Health-sector responses to intimate partner violence in low- and middle-income settings: a review of current models, challenges and opportunities. *Bulletin of the World Health Organization* 2008;86(8):635-42.
26. EPOC. Synthesising results when it does not make sense to do a meta-analysis. EPOC Resources for review authors. https://epoc.cochrane.org/sites/epoc.cochrane.org/files/public/uploads/Resources-for-authors2017/synthesising_results_when_meta-analysis_does_not_make_sense.pdf, 2017.

For peer review only

27. Thomas J, Harden A. Methods for the thematic synthesis of qualitative research in systematic reviews. *BMC Med Res Methodol* 2008;8:45. doi: 10.1186/1471-2288-8-45 [published Online First: 2008/07/12]

28. Abeid M, Muganyizi P, Mpembeni R, et al. Evaluation of a training program for health care workers to improve the quality of care for rape survivors: a quasi-experimental design study in Morogoro, Tanzania. *Glob Health Action* 2016;9:31735. doi: https://dx.doi.org/10.3402/gha.v9.31735

29. Bress J, Kashemwa G, Amisi C, et al. Delivering integrated care after sexual violence in the Democratic Republic of the Congo. *BMJ Glob Health* 2019;4(1):e001120. doi: 10.1136/bmjgh-2018-001120 [published Online First: 2019/03/23]

30. Brown LL, Van Zyl MAR. Mitigating intimate partner violence among South African women testing HIV positive during mobile counseling and testing. *Aids Care-Psychol Socio-Med Asp Aids-Hiv* 2018;30(1):65-71. doi: 10.1080/09540121.2017.1366414

31. Christofides N, Jewkes R. Acceptability of universal screening for intimate partner violence in voluntary HIV testing and counseling services in South Africa and service implications. *AIDS Care* 2010;22(3):279-85. doi: https://dx.doi.org/10.1080/09540120903193617

32. Cockcroft A, Omer K, Gidado Y, et al. The impact of universal home visits with pregnant women and their spouses on maternal outcomes: a cluster randomised controlled trial in Bauchi State, Nigeria. *BMJ Glob Health* 2019;4(1):e001172. doi: 10.1136/bmjgh-2018-001172 [published Online First: 2019/03/23]

33. Haberland N, Ndwiga C, McCarthy K, et al. Addressing intimate partner violence and power in relationships in HIV testing services: Results of an intervention piloted in Nairobi, Kenya. HIVCore final report: Washington, D.C., USAID | Project Search: HIVCore, 2016:50 p.

34. Kim JC, Askew I, Muvhango L, et al. Comprehensive care and HIV prophylaxis after sexual assault in rural South Africa: the Refentse intervention study. *BMJ* 2009;338:b515. doi: https://dx.doi.org/10.1136/bmj.b515

35. Laisser RM, Nystrom L, Lindmark G, et al. Screening of women for intimate partner violence: a pilot intervention at an outpatient department in Tanzania. *Glob Health Action* 2011;4:7288. doi: 10.3402/gha.v4i0.7288 [published Online First: 2011/10/27]

36. Matseke G, Peltzer K, Habil. Screening and brief intervention for intimate partner violence among antenatal care attendees at primary healthcare clinics in Mpumalanga province, South Africa. *South African Journal of Obstetrics and Gynaecology* 2013;19(2):40-43. doi: http://dx.doi.org/10.7196/SAJOG.637

37. Mutisya RK, Ngure K, Mwachari C. A psychosocial intervention to reduce gender-based violence and antepartum depressive symptoms in pregnant women in Kisumu County, Kenya: A quasi-experimental study. *Pan African Medical Journal* 2018;29 (no pagination)(11) doi: http://dx.doi.org/10.11604/pamj.2018.29.11.13596

For peer review only

38. Samandari G, Delamou A, Traore P, et al. Integrating Intimate Partner Violence Screening and Counseling in a Family Planning Clinic: Evaluation of a Pilot Project in Conakry, Guinea. *African Journal of Reproductive Health* 2016;20(2):86-93.

39. Settergren SK, Mujaya S, Rida W, et al. Cluster randomized trial of comprehensive gender-based violence programming delivered through the HIV/AIDS program platform in Mbeya Region, Tanzania: Tathmini GBV study. *PLoS One* 2018;13(12):e0206074. doi: 10.1371/journal.pone.0206074 [published Online First: 2018/12/07]

40. Sikkema KJ, Mulawa MI, Robertson C, et al. Improving AIDS Care After Trauma (ImpACT): Pilot Outcomes of a Coping intervention Among HIV-Infected Women with Sexual Trauma in South Africa. *AIDS & Behavior* 2018;22(3):1039-52. doi: 10.1007/s10461-017-2013-1

41. Sithole Z, Gombe NT, Juru T, et al. Evaluation of sexual and gender-based violence program in Harare City, Zimbabwe, 2016: a descriptive cross-sectional study. *Pan Afr Med J* 2018;31:200. doi: 10.11604/pamj.2018.31.200.14791 [published Online First: 2019/05/16]

42. Smith JR, Ho LS, Langston A, et al. Clinical care for sexual assault survivors multimedia training: a mixed-methods study of effect on healthcare providers' attitudes, knowledge, confidence, and practice in humanitarian settings. *Confl Health* 2013;7:14. doi: https://dx.doi.org/10.1186/1752-1505-7-14

43. Turan JM, Hatcher AM, Odero M, et al. A Community-Supported Clinic-Based Program for Prevention of Violence against Pregnant Women in Rural Kenya. *AIDS Res Treat* 2013;2013:736926. doi: https://dx.doi.org/10.1155/2013/736926

44. Undie C-C, Maternowska M, Mak'anyengo M, et al. Is routine screening for intimate partner violence feasible in public health care settings in Kenya? *Journal of Interpersonal Violence* 2016;31(2):282-301. doi: http://dx.doi.org/10.1177/0886260514555724

45. Wagman JA, Gray RH, Campbell JC, et al. Effectiveness of an integrated intimate partner violence and HIV prevention intervention in Rakai, Uganda: analysis of an intervention in an existing cluster randomised cohort. *Lancet Glob Health* 2015;3(1):e23-33. doi: 10.1016/S2214-109X(14)70344-4 [published Online First: 2014/12/30]

46. Khalili Z, Navaee M, Shakiba M, et al. [The effect of supportive-educational intervention on psychological distress in pregnant women subjected to domestic violence: A randomized controlled trial]. *Hayat, Journal of School of Nursing and Midwifery, Tehran University of Medical Sciences* 2019;25(2):151-67 (Persian).

47. Taghizadeh Z, Pourbakhtiar M, Ghasemzadeh S, et al. The effect of training problem-solving skills for pregnant women experiencing intimate partner violence: a randomized control trial. *Pan African Medical Journal* 2018;30:11. doi: 10.11604/pamj.2018.30.79.14872

48. Vakily M, Noroozi M, Yamani N. Comparing the effect of group-based and compact disk-based training on midwives' knowledge and attitude toward domestic violence in women of

reproductive age. *Journal of Education and Health Promotion* 2017;6:70. doi: http://dx.doi.org/10.4103/jehp.jehp_44_16

49. Arora S, Deosthali PB, Rege S. Effectiveness of a counselling intervention implemented in antenatal setting for pregnant women facing domestic violence: a pre-experimental study. *BJOG* 2019;126 Suppl 4:50-57. doi: 10.1111/1471-0528.15846 [published Online First: 2019/07/02]

50. Jayatilleke AC, Yoshikawa K, Yasuoka J, et al. Training Sri Lankan public health midwives on intimate partner violence: a pre- and post-intervention study. *BMC Public Health* 2015;15:331. doi: https://dx.doi.org/10.1186/s12889-015-1674-9

51. Sapkota D, Baird K, Saito A, et al. Antenatal-Based Pilot Psychosocial Intervention to Enhance Mental Health of Pregnant Women Experiencing Domestic and Family Violence in Nepal. *J Interpers Violence* 2020:886260520948151. doi: 10.1177/0886260520948151 [published Online First: 2020/08/20]

52. Bott S, Guedes A, Guezmes A, et al. Improving the health sector response to gender-based violence: A resource manual for health care professionals in developing countries. https://www.paho.org/hq/index.php?option=com_content&view=article&id=4517:2010-improving-health-sector-response-gender-violence-manual&Itemid=41342&lang=en: International Planned Parenthood Federation, Western Hemisphere Region., 2004:248.

53. Cripe SM, Sanchez SE, Sanchez E, et al. Intimate partner violence during pregnancy: a pilot intervention program in Lima, Peru. *Journal of Interpersonal Violence* 2010;25(11):2054-76. doi: https://dx.doi.org/10.1177/0886260509354517

54. Kim J, Mokwena L, Ntlemo E, et al. Developing an integrated model for post-rape care and HIV post-exposure prophylaxis in rural South Africa: [Johannesburg], South Africa, Population Council, Frontiers in Reproductive Health, 2007 Nov., 2007:[52] p.

55. Knettel BA, Mulawa MI, Knippler ET, et al. Women's perspectives on ImpACT: a coping intervention to address sexual trauma and improve HIV care engagement in Cape Town, South Africa. *AIDS Care* 2019;31(11):1389-96. doi: 10.1080/09540121.2019.1587368 [published Online First: 2019/03/02]

56. Sapkota D, Baird K, Saito A, et al. 'We don't see because we don't ask': Qualitative exploration of service users' and health professionals' views regarding a psychosocial intervention targeting pregnant women experiencing domestic and family violence. *PLoS One* 2020;15(3):e0230069. doi: 10.1371/journal.pone.0230069 [published Online First: 2020/03/10]

57. Guedes A, Bott S, Cuca Y. Integrating systematic screening for gender-based violence into sexual and reproductive health services: results of a baseline study by the International Planned Parenthood Federation, Western Hemisphere Region. *Int J Gynaecol Obstet* 2002;78 Suppl 1:S57-63. doi: 10.1016/S0020-7292(02)00045-0 [published Online First: 2002/11/14]

For peer review only

58. Rogow D. Living up to their name: Profamilia takes on gender-based violence. In: Rogow D, ed. Quality/Calidad/Qualité. https://pdfs.semanticscholar.org/e31a/25ccf1420d3c63c1dbfbc99000318e27ca1b.pdf: Population Council, 2006:35.

59. Delamou A, Samandari G. Integrating intimate partner violence screening and counseling with family planning services: Experience in Conakry, Guinea. The RESPOND Project Study Series: Contributions to Global Knowledge—Report No. 16. The RESPOND Project Study Series: Contributions to Global Knowledge. http://www.respond-project.org/pages/pubs/research-reports.php, 2014:78.

60. Arango D, Morton M, Gennari F, et al. Interventions to reduce or prevent violence against women and girls: a systematic review of reviews. Women's Voice, Agency and Participation Research Series, ed. Washington DC: World Bank 2014.

61. Jahanfar S, Howard LM, Medley N. Interventions for preventing or reducing domestic violence against pregnant women. *Cochrane Database of Systematic Reviews* 2014(11) doi: 10.1002/14651858.CD009414.pub3

62. Van Parys AS, Verhamme A, Temmerman M, et al. Intimate partner violence and pregnancy: a systematic review of interventions. *PLoS One* 2014;9(1):e85084. doi: 10.1371/journal.pone.0085084 [published Online First: 2014/02/01]

63. Turner DT, Riedel E, Kobeissi LH, et al. Psychosocial interventions for intimate partner violence in low and middle income countries: A meta-analysis of randomised controlled trials. *J Glob Health* 2020;10(1):010409. doi: 10.7189/jogh.10.010409 [published Online First: 2020/05/07]

64. Tarzia L, Bohren MA, Cameron J, et al. Women's experiences and expectations after disclosure of intimate partner abuse to a healthcare provider: A qualitative meta-synthesis. *BMJ Open* 2020;10(11):e041339. doi: 10.1136/bmjopen-2020-041339 [published Online First: 2020/11/29]

65. Colombini M, Dockerty C, Mayhew SH. Barriers and Facilitators to Integrating Health Service Responses to Intimate Partner Violence in Low- and Middle-Income Countries: A Comparative Health Systems and Service Analysis. *Studies in Family Planning* 2017;48(2):179-200. doi: https://dx.doi.org/10.1111/sifp.12021

Figure 1. Flow diagram.

Figure 2. Process-oriented logic model of interventions in sexual and reproductive health services addressing violence against women in low- and middle- income countries.

For peer review only

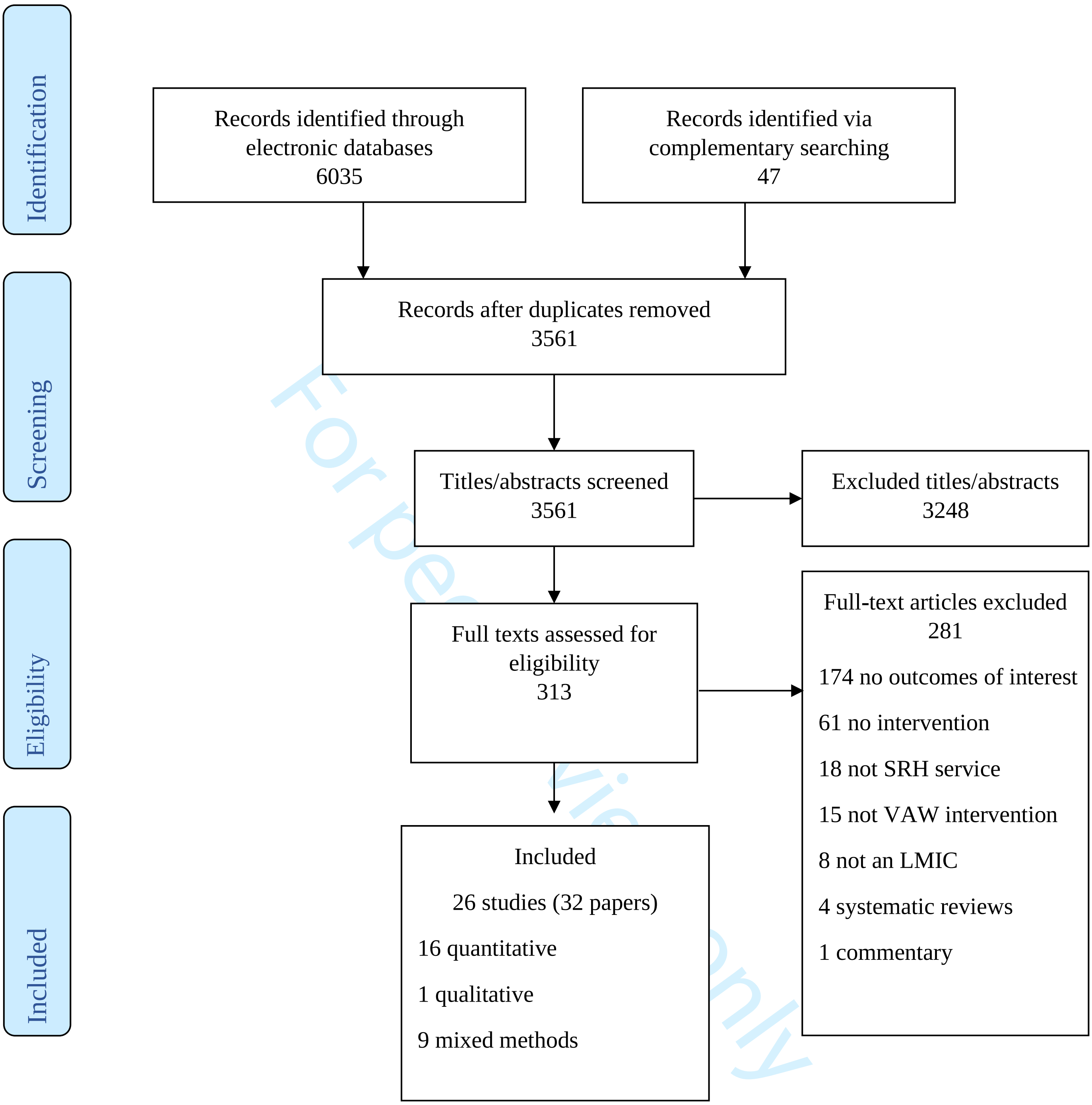

Figure 1. Flow diagram

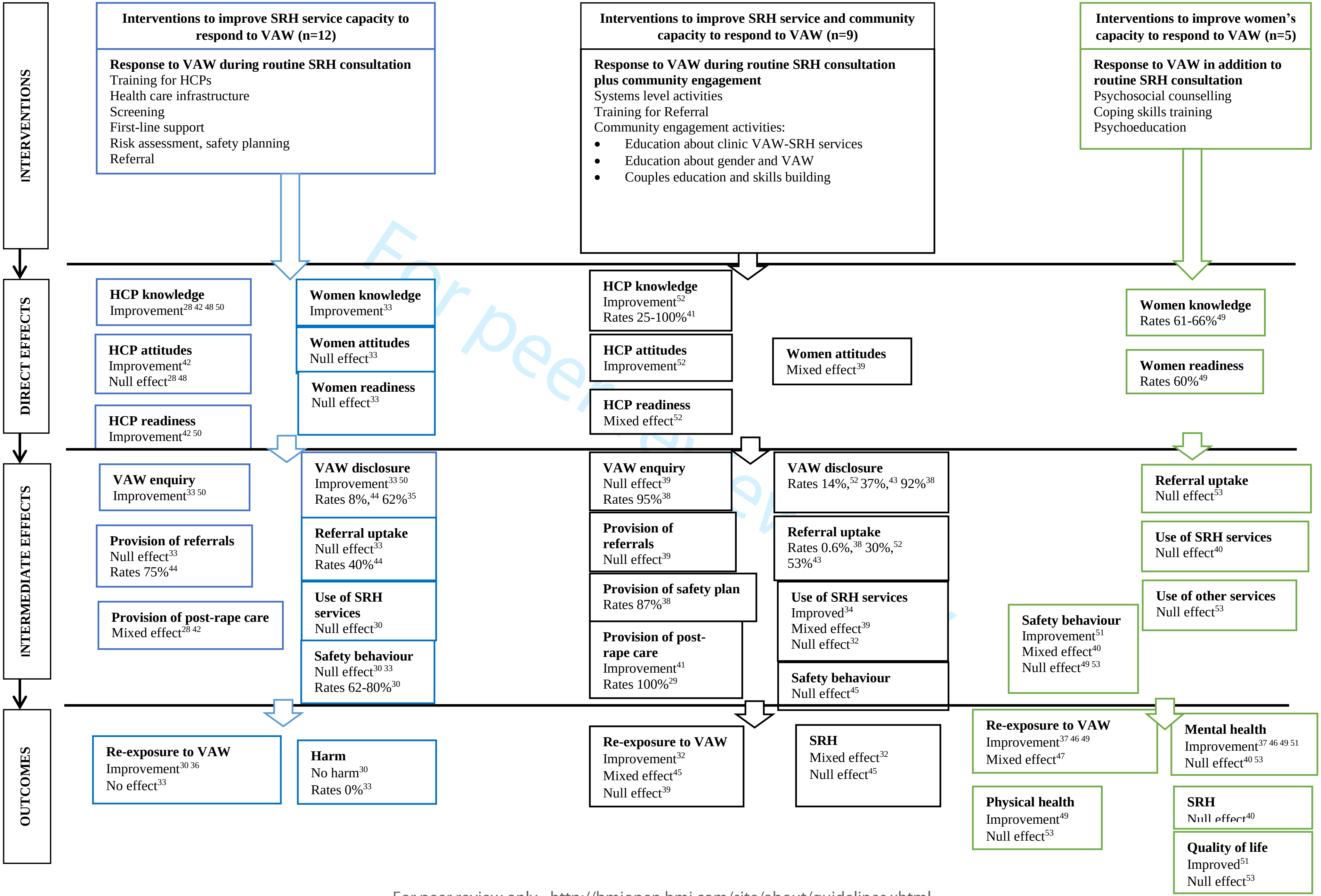
INTERVENTIONS
DIRECT EFFECTS
INTERMEDIATE EFFECTS
OUTCOMES
Interventions to improve SRH service capacity to respond to VAW (n=12)
Response to VAW during routine SRH consultation
Training for HCPs
Health care infrastructure
Screening
First-line support
Risk assessment, safety planning
Referral
Interventions to improve SRH service and community capacity to respond to VAW (n=9)
Response to VAW during routine SRH consultation plus community engagement
Systems level activities
Training for Referral
Community engagement activities:
• Education about clinic VAW-SRH services
• Education about gender and VAW
• Couples education and skills building
Interventions to improve women's capacity to respond to VAW (n=5)
Response to VAW in addition to routine SRH consultation
Psychosocial counselling
Coping skills training
Psychoeducation
HCP knowledge
Improvement[28 42 48 50]
HCP attitudes
Improvement[42]
Null effect[28 48]
HCP readiness
Improvement[42 50]
Women knowledge
Improvement[33]
Women attitudes
Null effect[33]
Women readiness
Null effect[33]
HCP knowledge
Improvement[52]
Rates 25-100%[41]
HCP attitudes
Improvement[52]
HCP readiness
Mixed effect[52]
Women attitudes
Mixed effect[39]
Women knowledge
Rates 61-66%[49]
Women readiness
Rates 60%[49]
VAW enquiry
Improvement[33 50]
Provision of referrals
Null effect[33]
Rates 75%[44]
Provision of post-rape care
Mixed effect[28 42]
VAW disclosure
Improvement[33 50]
Rates 8%,[44] 62%[35]
Referral uptake
Null effect[33]
Rates 40%[44]
Use of SRH services
Null effect[30]
Safety behaviour
Null effect[30 33]
Rates 62-80%[30]
VAW enquiry
Null effect[39]
Rates 95%[38]
Provision of referrals
Null effect[39]
Provision of safety plan
Rates 87%[38]
Provision of post-rape care
Improvement[41]
Rates 100%[29]
VAW disclosure
Rates 14%,[52] 37%,[43] 92%[38]
Referral uptake
Rates 0.6%,[38] 30%,[52] 53%[43]
Use of SRH services
Improved[34]
Mixed effect[39]
Null effect[32]
Safety behaviour
Null effect[45]
Referral uptake
Null effect[53]
Use of SRH services
Null effect[40]
Use of other services
Null effect[53]
Safety behaviour
Improvement[51]
Mixed effect[40]
Null effect[49 53]
Re-exposure to VAW
Improvement[30 36]
No effect[33]
Harm
No harm[30]
Rates 0%[33]
Re-exposure to VAW
Improvement[32]
Mixed effect[45]
Null effect[39]
SRH
Mixed effect[32]
Null effect[45]
Re-exposure to VAW
Improvement[37 46 49]
Mixed effect[47]
Mental health
Improvement[37 46 49 51]
Null effect[40 53]
Physical health
Improvement[49]
Null effect[53]
SRH
Null effect[40]
Quality of life
Improved[51]
Null effect[53]

Supplementary file 1. Search strategy

Searched 20 August 18
Databases:
(VAW AND Interventions AND LMICs AND healthcare) less exclusions
See attached Medline strategy
Grey literature:
("violence against women" OR "intimate partner violence" "domestic violence" OR DVA OR IPV OR VAW) AND (intervention* or prevention OR trial*)

Search results (databases):
Medline/Premedline = 1464
Embase= 1403
Psycinfo= 594
Cochrane =61
Cinahl=314
IMEMR =5
Web of Science= 920
Popline= 880
Lilacs= 392
WHO RHL=2
Total= 6035
Total deduplicated =3514

Search grey literature:
UNFA=8
SVRI = 5
JPHIEGO =3
USAID =4
WHO (IRIS) SEARO =2
WHO (IRIS) EMRO =3
Google=1
Google Scholar=1
ClinicalTrials.gov =15
WORLD Bank
OTHER= 1
Total= 45
Total deduplicated against database search= 43

Grand Total (databases and grey lit) =3557

Database: Ovid MEDLINE(R) and Epub Ahead of Print, In-Process & Other Non-Indexed Citations and Daily <1946 to August 20, 2018>
Search Strategy:
----
1 rape/ or domestic violence/ or exp intimate partner violence/ or battered women/ or Gender-Based Violence/ (19658)
2 (violence/ or sex offenses/ or sexual harassment/ or homicide/ or physical abuse/ or coercion/ or crime victims/) and (female/ or women/ or spouses/ or marriage/ or Sexual partners/) (29381)
3 (violence/ or sex offenses/ or sexual harassment/ or homicide/ or physical abuse/ or coercion/ or crime victims/) and (female* or domestic or spous* or partner* or woman or women or married or marriage* or marital or husband* or wife or wives or boyfriend* or girlfriend* or gender-based or non-partner).tw. (13114)

For peer review only

4 ((sexual abuse or sexual harassment or sexual coercion or violent or violence or assault* or beat or beating or batter* or rape* or sex offense* or sexual offense*) adj4 (female* or domestic or spous* or partner* or woman or women or married or marriage* or marital or husband* or wife or wives or boyfriend* or girlfriend* or gender-based or non-partner)).tw. (18139)
5 (IPV or DVA).tw. (5675)
6 (VAW or date rape).tw. (309)
7 ((woman or women) adj3 relationship* adj3 abus*).tw. (90)
8 ((birth control or fertility control or reproductiv* or contraceptiv* or contraception) adj3 (sabotag* or coerc*)).tw. (105)
9 or/1-8 (52401)
10 ((prevent* or intervention* or eliminat* or program* or approach or approaches or trial* or response* or effective or effectiveness or identify or efficacy or what works or outcome* or treatment* or therap* or identification) adj12 (violent or violence or rape* or DVA or IPV or VAW or harassment or sexual offense* or sex offense* or abus* or assault* or beating or beat or coerc* or female* or domestic or spous* or partner* or woman or women or married or marriage* or marital or husband* or wife or wives or boyfriend* or girlfriend* or gender-based or non-partner)).tw. (398870)
11 rape/pc or sex offenses/pc or domestic violence/pc or exp intimate partner violence/pc or battered women/pc or Gender-Based Violence/pc or Sexual Harassment/pc (4812)
12 (psychosocial support or psychological support or education* or training or home visit* or advocacy).tw. (776643)
13 secondary prevention/ or tertiary prevention/ (18314)
14 ((questioning or interviewing or empower*) adj3 (method* or technique*)).tw. (1434)
15 (patient adj3 information).tw. (16715)
16 ((poster* or information or pamphlet* or leaflet*) adj3 (provision or provide*)).tw. (172170)
17 counsel?ing.ti,ab. (81732)
18 exp counseling/ (40659)
19 exp Clinical Trials as Topic/ (316975)
20 exp clinical trial/ (805706)
21 "Controlled Before-After Studies"/ (348)
22 "outcome and process assessment (health care)"/ or "process assessment (health care)"/ (29451)
23 ((program* or process* or service) adj3 evaluation*).tw. (22711)
24 (pretest* or pre-test* or posttest* or post-test* or pre-intervention* or preintervention* or postintervention* or post-intervention*).tw. (52054)
25 (pre* adj12 post*).tw. (579088)
26 ("before and after" or before-after).tw. (243968)
27 or/10-26 (2980515)
28 9 and 27 (24093)
29 Developing Countries/ (70541)
30 (developing countr* or emerging econom* or third world).tw. (55717)
31 ((low or middle) adj4 income countr*).ti,ab. (16763)
32 LMIC*.ti,ab. (2703)
33 (Afghanistan or Benin or Burkina Faso or Burundi or Cambodia or Central Africa or Chad or Comoros or Congo or Eritrea or Ethiopia or Gambia or Guinea or Bissau or Haiti or North Korea or Liberia or Madagascar or Malawi or Mali or Mozambique or Nepal or Niger or Rwanda or Sierra Leone or Somalia or Tanzania or Togo or Uganda or Zimbabwe).mp. (289340)
34 (Armenia or Bangladesh or Bhutan or Bolivia or Cabo Verde or Cameroon or Cote d'Ivoire or Ivory Coast or Djibouti or Egypt or El Salvador or Georgia or Ghana or Guatemala or Guyana or Honduras or India or Indonesia or Kenya or Kiribati or Kosovo or Kyrgyz* or Lao or Laos or Lesotho or Mauritania or Micronesia or Moldova or Mongolia or Morocco or Myanmar or Nicaragua or Nigeria or Pakistan or Philippines or Samoa or Sao Tome or

For peer review only - http://bmjopen.bmj.com/site/about/guidelines.xhtml

Principe or Senegal or Solomon Islands or Sri Lanka or Sudan or Swaziland or Syria* or Tajikistan or Timor Leste or Ukraine or Uzbekistan or Vanuatu or Vietnam or West Bank or Gaza or Yemen or Zambia).mp. (392622)
35 (Albania or Angola or Argentina or Panama or Tunisia or Palau or Tunisia or Herzegovina or Fiji or Namibia or Algeria or Gabon or Nauru or Grenada or Paraguay or Peru or Azerbaijan or Grenadines or Romania or Belarus or Iran or Russia* or Belize or Iraq or Bosnia or Jamaica or Serbia).mp. (189750)
36 (Botswana or Jordan or South Africa or Brazil or Kazakhstan or Saint Lucia or St Lucia or Bulgaria or Lebanon or Saint Vincent or St Vincent or China or Libya or Suriname or Colombia or Macedonia or Thailand or Costa Rica or Malaysia or Tonga).mp. (435209)
37 (Cuba or Maldives or Turkey or Dominica* or Marshall Islands or Turkmenistan or Mauritius or Tuvalu or Mexico or Venezuela or Ecuador or Montenegro).mp. (122538)
38 or/29-37 (1403313)
39 28 and 38 (4702)
40 exp maternal health services/ or exp reproductive health services/ or family planning services/ (67984)
41 exp pregnancy/ or exp pregnancy trimesters/ or pregnant women/ or peripartum period/ or exp pregnancy complications/ or exp fetal therapies/ or exp Obstetric surgical procedures/ or exp postpartum period/ or obstetric nursing/ or midwifery/ (914606)
42 exp maternal-child nursing/ (5549)
43 (adolescent health services/ or community mental health services/ or community health services/ or rural health services/ or rural nursing/ or family health/ or adolescent health/ or exp primary health care/ or exp general practice/ or general practitioners/ or physicians, family/) and (women or woman or reproductive or sexual health* or "STI" or STD* or "STIS" or contracept* or abortion or childbirth or pregnan*).mp. (26039)
44 reproductive medicine/ or gynecology/ or obstetrics/ or "Obstetrics and Gynecology Department, Hospital"/ (35849)
45 ((sexual or reproductive) adj3 (education or healthcare or care or service* or program* or clinic*)).mp. (12944)
46 ((sexual or reproductive) adj3 (education or health* or care)).jn,in. (14583)
47 ((pregnan* or birth or childbirth or midwife* or midwive* or " mother and baby" or obstetric* or maternal or maternity or postpartum or antepartum or postnatal or post-natal or ante-natal or antenatal or prenatal or pre-natal or perinatal or peri-natal or contraception or contraceptiv* or abortion or fertility or gynae* or gyne* or STD* or "STI" or "STIS" or sexually transmitted or PMS or premenstrual syndrome) adj3 (care or healthcare or clinic* or service* or treatment*)).tw. (109386)
48 (cervical adj2 (smear* or screening)).tw. (11930)
49 vaginal smears/ or papanicolaou test/ (22149)
50 exp Sexually Transmitted Diseases/di, pc, rh, th (103211)
51 exp Women's Health/ (26647)
52 exp Menstruation Disturbances/di, pc, rh, th (5369)
53 ((woman* or women*) adj3 health*).jn,in,mp. (106372)
54 family planning*.jn,mp,in. (50733)
55 exp "diagnostic techniques, obstetrical and gynecological"/ (124519)
56 reproductive health/ or sexual health/ (2688)
57 or/40-56 (1238548)
58 39 and 57 (1822)
59 letter/ (997536)
60 editorial/ (466054)
61 news/ (190965)
62 exp historical article/ (382354)
63 Anecdotes as topic/ (4721)
64 comment/ (730950)
65 (letter or editorial or comment*).ti. (160815)
66 exp animals/ not humans/ (4489180)

For peer review only

For peer review only - http://bmjopen.bmj.com/site/about/guidelines.xhtml

67 exp Animals, Laboratory/ (820540)
68 exp Animal Experimentation/ (8786)
69 exp Models, Animal/ (516652)
70 exp rodentia/ (3047766)
71 (rat or rats or mouse or mice or rodent* or animal*).ti. (1394667)
72 or/59-71 (7516664)
73 58 not 72 (1776)
74 limit 73 to yr="2005 -Current" (1464)

For peer review only

For peer review only - http://bmjopen.bmj.com/site/about/guidelines.xhtml

Online supplementary file 2. Quality appraisal

Risk of bias in randomised controlled trials

Study ID	Bias arising from the randomization process	Bias due to deviations from the intended interventions (assignment)	Bias due to deviations from the intended interventions (adherence)	Bias due to missing outcome data	Bias in measurement of the outcome	Bias in selection of the reported result	Overall risk of bias
Brown 2018[30]	Low	Some concerns	High	Low	Low	Low	High
Cockcroft 2019[32]	Low	Low	Low	Low	Low	Low	Low
Cripe 2013[53]	Low	Low	Low	Low	Some concerns	Some concerns	Some concerns
Haberland 2016[33]	Some concerns	Low	High	Low	Some concerns	Some concerns	High
Khalili 2020[46]	Low	High	High	Low	High	Low	High
Mutisya 2018[37]	Low	High	High	Low	High	Some concerns	High
Sapkota 2020[51]	Low	Low	Low	Low	Low	Low	Low
Settergren 2018[39]	Low	Low	High	Low	High	Low	High
Sikkema 2018[40]	Low	Low	Low	High	Some concerns	Some concerns	High
Tanghizadeh 2018[47]	Low	Low	Low	Low	Low	Some concerns	Some concerns
Vakily 2017[48]	Some concerns	Some concerns	High	High	Low	Some concerns	High
Wagman 2015[45]	Some concerns	High	Some concerns	Low	High	Some concerns	High

Risk of bias (EPOC criteria) in controlled before-after studies

Study ID	Was the allocation sequence adequately generated?	Was the allocation adequately concealed?	Were baseline outcome measurements similar?	Were baseline characteristics similar?	Were incomplete outcome data adequately addressed?	Was knowledge of the allocated interventions adequately prevented during the study?	Was the study adequately protected against contamination ?	Was the study free from selective outcome reporting?	Was the study free from other sources of bias?	Overall risk of bias
Abeid 2016[28]	No	No	Yes	Yes	Yes	Unclear	Unclear	Yes	Unclear	High

Risk of bias (EPOC criteria) in studies without a control group

Study ID	Was the intervention independent of other changes?	Was the shape of the intervention effect pre-specified?	Was the intervention unlikely to affect data collection?	Was knowledge of the allocated interventions adequately prevented during the study?	Were incomplete outcome data adequately addressed?	Was the study free from selective outcome reporting?	Was the study free from other risk of bias?	Overall risk of bias
Arora 2019[49]	No	Yes	Yes	No	Yes	No	Unclear	High
Bott 2004[52]	Unclear	Unclear	Yes	No	No	Unclear	Unclear	High
Bress 2019[29]	No	No	No	No	Unclear	Yes	Unclear	High

Study ID	Was the intervention independent of other changes?	Was the shape of the intervention effect pre-specified?	Was the intervention unlikely to affect data collection?	Was knowledge of the allocated interventions adequately prevented during the study?	Were incomplete outcome data adequately addressed?	Was the study free from selective outcome reporting?	Was the study free from other risk of bias?	Overall risk of bias
Jayatilleke 2015[50]	Unclear	Yes	Yes	No	Unclear	Yes	Unclear	High
Kim 2007[54]	No	Yes	Yes	No	Unclear	No	No	High
Laisser 2011[35]	No	No	No	No	Unclear	Yes	Unclear	High
Matseke 2013[36]	Unclear	Yes	Yes	No	No	Yes	No	High
Samandari 2016[38]	No	Yes	Yes	No	Yes	Yes	Unclear	High
Sithole 2018[41]	Unclear	No	No	No	Unclear	No	Unclear	High
Smith 2013[42]	No	Yes	Yes	Unclear	Yes	Yes	No	High
Turan 2013[43]	Unclear	No	No	No	Unclear	Yes	Unclear	High
Undie 2016[44]	No	No	No	No	Unclear	Unclear	Unclear	High

Quality appraisal of qualitative studies

CASP signalling questions	Bott 2004[52]	Christofides 2010[31]	Haberland 2016[33]	Laisser 2011[35]	Samandari 2016[38]	Sapkota 2020[56]	Sikkema 2018[55]	Smith 2013[42]	Turan 2013[43]	Undie 2016[44]
1. Interprets subjective experiences?	Yes	Yes	Yes	Yes	Yes	Yes	Yes	Yes	Yes	Yes
2. Right methodology?	Yes	Yes	Yes	Yes	Yes	Yes	Yes	Yes	Yes	Yes
3. Appropriate design?	Yes	Yes	Yes	Yes	Yes	Yes	Yes	Yes	Yes	Yes
4. Design justified?	Yes	No	No	Yes	No	Yes	Yes	No	No	No
5. Ethical issues considered?	No	No	Yes	Yes	Yes	Yes	Yes	Yes	Yes	Yes
6. Credibility established?	Yes	No	No	Yes	Yes	Yes	Yes	Yes	Yes	Yes
7. Transferability established?	No	Yes	No	Yes	Yes	Yes	Yes	Yes	No	No
8. Purpose established?	Yes	Yes	Yes	Yes	Yes	Yes	Yes	Yes	No	Yes
9. Recruitment appropriate?	Yes	Yes	Yes	Yes	Yes	Yes	Yes	Yes	Yes	Yes
10. Selection of participants explained?	No	Yes	Yes	Yes	Yes	Yes	Yes	No	Yes	No
11. Participants appropriate?	No	No	No	Yes	Yes	No	Yes	Yes	Yes	No
12. Discussed recruitment?	No	Yes	No	No	No	No	Yes	No	No	No
13. Justified setting?	Yes	Yes	Yes	Yes	Yes	Yes	Yes	Yes	Yes	No
14. How data were collected?	Yes	Yes	Yes	Yes	Yes	Yes	Yes	Yes	Yes	Yes
15. Justified data collection method?	No	No	Unsure	Yes	No	Yes	Yes	No	No	No

For peer review only - http://bmjopen.bmj.com/site/about/guidelines.xhtml

CASP signalling questions	Bott 2004[52]	Christofides 2010[31]	Haberland 2016[33]	Laisser 2011[35]	Samandari 2016[38]	Sapkota 2020[56]	Sikkema 2018[55]	Smith 2013[42]	Turan 2013[43]	Undie 2016[44]
16. Described data collection method?	No	Yes	Yes	Yes	Yes	Yes	Yes	Yes	Yes	Yes
17. Form of data clear?	No	Yes	Yes	Yes	Yes	Yes	Yes	Yes	Yes	Yes
18. Described how data were reduced/transformed for analysis?	No	No	No	Yes	Yes	Yes	Yes	Yes	Yes	Yes
19. Discussed interpretation of findings?	Yes	No	No	Yes	Yes	Yes	Yes	No	Yes	Yes
20. Ensured neutrality?	No	No	No	No	Yes	Yes	Yes	No	Yes	No
**Total (Yes/No/Unsure)**	**10/10/0**	**12/8/0**	**11/8/1**	**18/2/0**	**17/3/0**	**18/2/0**	**20/0/0**	**14/6/0**	**15/5/0**	**12/8/0**

For peer review only

Online supplementary file 3. Included interventions mapped on the Health Systems Wheel framework and models of service integration

Study ID	Study design	Leadership and governance	Multi-sectoral coordination	Workforce development	Health-care delivery	Infrastructure	Financing	Information	Level of VAW service integration
Abeid 2016[28]	CBA			●	●	●		●	Systems
Arora 2019[49]	UBA				●				Provider
Bott 2014[52]	UBA	●	●	●	●	●	●	●	Facility
Bress 2018[29]	Cross-sectional		●	●	●	●		●	Provider
Brown 2018[30]	RCT				●				Facility
Cockcroft 2019[32]	cRCT			●	●	●		●	Provider
Cripe 2010[53]	RCT			●	●				Provider
Cristofides 2010[31]	Qualitative			●	●				Facility
Haberland 2016[33]	RCT			●	●	●	●	●	Facility
Jayatilleke 2015[50]	UBA			●	●				Provider
Khalili 2020[46]	RCT				●				Provider
Kim 2007[54]	UBA	●		●	●	●		●	Facility
Laisser 2011[35]	Cross-sectional			●	●			●	Systems
Matseke 2013[36]	UBA			●	●				Systems
Mutisya 2018[37]	RCT				●		●		Provider
Samandari 2016[38]	Cross-sectional	●	●	●	●	●		●	Systems
Sapkota 2020[51]	RCT				●		●		Provider
Settergren 2018[39]	cRCT	●	●	●	●	●		●	Systems
Sikkema 2018[40]	RCT			●	●				Provider
Sithole 2018[41]	Cross-sectional		●	●	●	●		●	Facility
Smith 2013[42]	UBA			●					Facility
Taghizadeh 2018[47]	RCT				●				Provider
Turan 2013[43]	Cross-sectional		●	●	●	●	●	●	Systems
Undie 2016[44]	Cross-sectional			●	●		●	●	Facility
Vakily 2018[48]	RCT			●					
Wagman 2015[45]	cRCT		●	●	●				Facility

Note. VAW violence against women. RCT randomised controlled trial. cRCT cluster randomised controlled trial. CBA controlled before-after. UBA uncontrolled before-after. Provider-level integration when one trained health care provider delivers most of the VAW work. Facility-level integration when several trained HCPs deliver most VAW work within one heath care facility. Systems-level integration when trained HCP identifies patients affected by VAW, provides first-line support and clinical care, and then refers them to higher level facilities with VAW specialist or external VAW services.

For peer review only

Supplementary file 4. Effects and outcomes of interventions on response to VAW during routine SRH consultation

Outcomes	Results (95% CI, p)	Effect (95% CI)	N of participants (design/follow up)	Study	Risk of bias	Direction of effect/interpretation
**Direct effects**						
**HCP knowledge about VAW, relevant procedures (n=4)**						
Mean (SD) score of knowledge about domestic violence, post-intervention	Group training = 16.1 (1.9)<br>CD training = 17.7 (1.1)<br>p<0.001	-	35 HCPs (RCT/2 month)	Vakily 2017[48]	High	2-hour CD training improved HCP knowledge about domestic violence more than group training
Change in proportion with correct knowledge on sexual violence	Intervention = 31.4%<br>Control = -22.3%	Net effect = 53.7% (32.2; 75.1)	HCPs (CBA/12 months	Abeid 2016[28]	High	5-day training, guidelines, infrastructure improvement improved HCPs knowledge about sexual violence
Median (IQR) score of knowledge about IPV	Pre-intervention = 0.62 (0.43-0.81)<br>Post-intervention = 0.88 (0.82-0.94)<br>p<0.001	-	408 HCPs (UBA/6 months)	Jayatilleke 2015[50]	High	4-day training, handbook, external referral improved HCP knowledge about IPV
Mean (95% CI) score of knowledge in providing care to sexual assault survivors	Pre-intervention = 49.09 (45.57; 51.34)<br>Post-intervention = 61.59 (59.04; 64.42)	MD = 12.50 (10.29; 16.24)	106 HCPs (UBA/3 months)	Smith 2013[42]	High	4-day training, infrastructure improvement contributed towards improved HCP knowledge about providing clinical care to survivors of sexual violence
**HCP attitudes about VAW (n=3)**						
Mean (SD) score of attitudes about domestic violence, post-intervention	Group training = 46.9 (4.9)<br>CD training = 45.4 (6.4)<br>p = 0.3	-	35 HCPs (RCT/2 month)	Vakily 2017[48]	High	Neither group nor CD 2-hour training had effect on HCP attitudes about domestic violence
Mean (95% CI) score of attitudes about sexual violence	Pre-intervention = 71.76 (66.79; 73.14)<br>Post-intervention = 77.20 (72.53; 78.34)	MD = 5.44 (1.89; 8.98)	106 HCPs (UBA/3 months)	Smith 2013[42]	High	4-day training, infrastructure improvement contributed towards improved attitudes about sexual violence
Change in proportion with accepting attitude towards sexual violence	Intervention = -4.1%<br>Control = 6.8%	Net effect = -10.9% (-27.2; 5.5)	HCPs (CBA/12 months	Abeid 2016[28]	High	5-day training, guidelines, infrastructure improvement had no effect on HCP attitudes about sexual violence
**HCP readiness for identifying and responding to VAW (n=2)**						
Median (IQR) score of perceived barriers to IPV identification and response	Pre-intervention = 2.43 (2.14-3.14)<br>Post-intervention = 1.14 (1.14-1.28)<br>p<0.001		408 HCPs (UBA/6 months)	Jayatilleke 2015[50]	High	4-day training, handbook, external referral reduced HCP perceived barriers to identifying and responding to IPV
Median (IQR) score of perceived responsibilities to identify and respond to IPV	Pre-intervention = 3.20 (2.80-3.95)<br>Post-intervention = 4.60 (4.20-4.80)<br>p<0.001					4-day training, handbook increased HCP perceived responsibility and self-confidence to identify and respond to IPV

For peer review only

Outcomes	Results (95% CI, p)	Effect (95% CI)	N of participants (design/follow up)	Study	Risk of bias	Direction of effect/interpretation
Median (IQR) score of self-confidence to identify and respond to IPV	Pre-intervention = 1.81 (1.38-2.12)<br>Post-intervention = 2.75 (2.62-2.88)<br>p<0.001					
Mean (95% CI) score of HCPs' confidence in providing care to sexual assault survivors	Pre-intervention = 58.16 (53.86; 63.90)<br>Post-intervention = 72.66 (66.21; 74.30)	MD = 14.50 (8.22; 20.77)	106 HCPs (UBA/3 months)	Smith 2013[42]	High	4-day training, infrastructure improvement contributed towards improved confidence in providing clinical care to sexual assault survivors
**Women knowledge about VAW (n=1)**						
Mean difference (95% CI) in women's IPV knowledge score, post-intervention	-	MD = 0.16<br>Crude β=0.176 (0.02; 0.033)<br>Adjusted β=0.155 (0.00-0.31)	337 women (RCT/1 month)	Haberland 2016[33]	High	HCP training and ongoing support, 29-minute integrated HIV-IPV consultation, referral to IPV counsellor in ANC clinic improved knowledge about IPV and women's rights among pregnant women
Mean (SD) score of learning about women's rights in relationship	Intervention = 2.6 (1.1)<br>Control = 2.0 (1.0)<br>p<0.0001	-				
**Women attitudes about VAW (n=1)**						
Proportion (n) who justified wife beating, post-intervention	Intervention = 18.3% (49/268)<br>Control = 21.8% (58/267)<br>p=0.33	-	337 women (RCT/1 month)	Haberlan 2016[33]	High	HCP training and ongoing support, 29-minute integrated HIV-IPV consultation, assisted onsite referral had no effect on attitudes about IPV among pregnant women
**Women readiness for addressing VAW (n=1)**						
Proportion (n) who felt more confident in how deserve to be treated, post-intervention	Intervention = 82% (73/107)<br>Control = 71.6% (73/134)<br>p=0.12	-	337 women (RCT/1 month)	Haberlan 2016[33]	High	HCP training and ongoing support, 29-minute integrated HIV-IPV consultation, assisted onsite referral had no effect on self-confidence among pregnant women
**Intermediate effects**						
**VAW enquiry rate (n=2)**						
Proportion (n) screened for IPV, post-intervention	Intervention = 76% (81/107)<br>Control = 22% (29/134)<br>p<0.0001	-	337 women (RCT/1 month)	Haberland 2016[33]	High	HCP training and ongoing support, 29-minute integrated HIV-IPV consultation, assisted onsite referral increased IPV enquiry rate
Proportion (n) who discussed IPV	Pre-intervention = 67.3% (201)<br>Post-intervention = 96.5% (387)<br>p<0.01	-	408 HCPs (UBA/6 months)	Jayatilleke 2015[50]	High	4-day HCP training, handbook, external referral increased IPV enquiry rate
**Provision of referrals to VAW services (n=2)**						
Proportion (n) referred to GBV centre of those disclosed, post-intervention	Intervention = 56% (19/34)<br>Control = 33% (3/9)<br>p=0.28	-	337 women (RCT/1 month)	Haberland 2016[33]	High	HCP training and ongoing support, 29-minute integrated HIV-IPV consultation, referral to IPV counsellor in ANC clinic had no effect on referral rate

Outcomes	Results (95% CI, p)	Effect (95% CI)	N of participants (design/follow up)	Study	Risk of bias	Direction of effect/interpretation
Proportion (n) referred to the medical officer or Heath/IPV services	Pre-intervention = 6.5% (13)<br>Post-intervention = 22.4% (87)<br>p not reported	-	408 HCPs (UBA/6 months)	Jayatilleke 2015[50]	High	4-day training, handbook, external referral had no effect on referral rates to external IPV services
Proportion (n) referred to GBV centre of those disclosed, post-intervention	75% (73/95)	-	1210 women (Cross sectional/7 months)	Undie 2016[44]	High	HCP training, integrated IPV-HIV consultation, assisted referral contributed towards 75% referral rate to on-site GBV centre
**Provision of post-rape care (n=2)**						
Change in proportion who used a rape kit	Intervention = 59.6%<br>Control = -4.5%	Net effect = 64.1% (46.7; 81.5)	100 HCPs (CBA/12 months)	Abeid 2016[28]	High	5-day training, guidelines, infrastructure improvement contributed towards improvement on 10 out of 18 indicators of post-rape care
Change in proportion who gave prophylactic treatment for STI	Intervention = 10.9%<br>Control = 3.4%	Net effect 7.5% = (-14.5; 29.5)				
Proportion of eligible patients who received emergency contraception	Pre-intervention = 50%<br>Post-intervention = 82%<br>p<0.01	-	60 patients (UBA/3 months)	Smith 2013[42]	High	4-day training, infrastructure improvement contributed towards improvement on 6 out of 10 indicators of post-rape care service
Proportion of eligible patients who received HIV post-exposure prophylaxis	Pre-intervention = 42%<br>Post-intervention = 92%<br>p<0.001	-				
Proportion of eligible patients who received STI prophylaxis and treatment	Pre-intervention = 45%<br>Post-intervention = 96%<br>p<0.01	-				
**VAW disclosure rate (n=4)**						
Proportion (n) who disclosed IPV of those screened, post-intervention	Intervention = 32% (34/107)<br>Control = 7% (9/134)<br>p<0.0001	-	337 women (RCT/1 month)	Haberland 2016[33]	High	HCP training and ongoing support, 29-minute integrated HIV-IPV consultation, referral to IPV counsellor in ANC clinic increased IPV identification rate
Proportion (n) who identified at least one IPV during past 3 months	Pre-intervention = 73.3% (299)<br>Post-intervention = 98.5% (402)<br>p<0.001	-	408 HCPs (UBA/6 months)	Jayatilleke 2015[50]	High	4-day training, handbook increased IPV identification rate
Proportion who disclosed IPV of those screened	62%	-	102 women (Cross-sectional/3 weeks)	Laisser 2011[35]	High	HCP training, infrastructure improvement, integrated ANC-IPV consultation, external referral contributed towards 62% IPV identification rate
Proportion (n) who disclosed IPV of those screened	8% (95/1210)	-	1210 women (Cross-sectional/7 months)	Undie 2016[44]	High	HCP training, integrated HIV-IPV consultation, assisted onsite referral contributed towards 8% IPV identification rate
**VAW referral uptake (n=2)**						
Proportion (n) who used GBV centre out of those referred, post-intervention	Intervention = 63% (12/19)<br>Control = 100% (3/3)<br>p=0.52	-	337 women (RCT/1 month)	Haberland 2016[33]	High	HCP training and ongoing support, 29-minute integrated HIV-IPV consultation, referral to IPV

Outcomes	Results (95% CI, p)	Effect (95% CI)	N of participants (design/follow up)	Study	Risk of bias	Direction of effect/interpretation
						counsellor in ANC clinic had no effect on uptake of referrals to on-site GBV centre
Proportion (n) who used GBV centre out of those referred, post-intervention	40% (29/73)	-	1210 women (Cross sectional/7 months)	Undie 2016[44]	High	HCP training, integrated IPV-HIV consultation, assisted onsite referral contributed towards 40% uptake of referrals to on-site GBV centre
**Use of SRH services (n=1)**						
Proportion (n) who were linked to medical care to receive lab reports on CD4 count and viral load, post-intervention	Intervention = 43.13% (69/160)<br>Control = 38.50% (30/78)<br>p = 0.493	-	166 women (RCT/1 month)	Brown 2018[30]	High	7-minute integrated HIV-IPV consultation over phone had no effect on uptake of HIV services among women with experience of IPV
**Safety behaviour (n=2)**						
Mean (SD) pre-post difference score of perceived risk and safety	Intervention = 0.33 (3.07)<br>Control = 0.13 (3.05)<br>p=0.278	-	166 women (RCT/1 month)	Brown 2018[30]	High	7-minute integrated HIV-IPV consultation over phone had no effect on perceived risk and safety among HIV-positive women with experience of IPV
Proportion (n) who used safety plan, post-intervention	Intervention = 61.88% (99/160)	-				Most HIV-positive women who received 7-minute integrated IPV-HIV consultation used safety plan and employed at least one safety strategy
Proportion (n) who employed at least one safety strategy	Intervention = 80% (128/160)	-				
Proportion (n) who took an action following the IPV-enhanced HIV counselling, post-intervention	Intervention = 45.5% (25/66)<br>Control = 30.5% (18/79)<br>p=0.073	-	337 women (RCT/1 month)	Haberland 2016[33]	High	HCP training and ongoing support, 29-minute integrated HIV-IPV consultation, referral to IPV counsellor in ANC clinic had no effect on coping behaviour and 7 behaviour indicators of HIV care among pregnant women
Proportion (n) who can ask partner to use a condom, post-intervention	Intervention = 58.3% (35/107)<br>Control = 51.2% (43/134)<br>p=0.31	-				
**Outcomes**						
**Re-exposure to VAW (n=3)**						
Proportion (n) who did not experience IPV upon partner notification of serostatus, post-intervention	Intervention = 96.9% (155/160)<br>Control = 88% (71/79)	OR = 4.37 (1.46; 13.44)	166 HIV-positive women (RCT/1 month)	Brown 2018[30]	High	7-minute integrated HIV-IPV consultation over phone consultation reduced IPV upon partner notification about serostatus among HIV-positive women
Proportion (n) who experienced any IPV, post-intervention	Intervention = 16.0% (43/337)<br>Control = 18.7% (50/351),<br>p=0.43	-	337 pregnant women (RCT/1 month)	Haberland 2016[33]	High	HCP training and ongoing support, 29-minute integrated HIV-IPV consultation, referral to IPV counsellor in ANC clinic had no effect on any IPV since baseline assessment
Mean (SD) danger assessment score	Pre-intervention = 6.02 (2.97)<br>Post-intervention = 2.82 (0.27)	MD = 3.20 (3.56) (2.43; 3.98)	84 women (UBA/3 months)	Matseke 2013[36]	High	HPC training, 30-minute integrated ANC-IPV consultation, external referral contributed towards reduction in potential risk of becoming a victim of femicide among pregnant women

For peer review only

Outcomes	Results (95% CI, p)	Effect (95% CI)	N of participants (design/follow up)	Study	Risk of bias	Direction of effect/interpretation
**Harm (n=2)**						
Proportion (n) who reported that service had not placed them in greater danger, post-intervention	Intervention = 96.25% (154/160)<br>Control = 93.67% (74/79)<br>p = 0.512	-	166 women (RCT/1 month)	Brown 2018[30]	High	7-minute integrated HIV-IPV consultation over phone did not put HIV-positive women in greater danger
Proportion who reported harmful effects	Intervention = 0	-	337 pregnant women (RCT/1 month)	Haberland 2016[33]	High	HCP training and ongoing support, 29-minute integrated HIV-IPV consultation, referral to IPV counsellor in ANC clinic had no harmful effect on pregnant women

Note. VAW violence against women. GBV gender-based violence. DV domestic violence. IPV intimate partner violence. SRH sexual and reproductive health. STI Sexually transmitted infections. HIV human immunodeficiency virus. ANC antenatal care. HCP health care provider. RCT randomised controlled trial. CBA controlled before-after study. UBA uncontrolled before-after study. CI confidence interval. SD standard deviation. IQR interquartile range. MD mean difference. OR odds ratio.

For peer review only

Supplementary file 5. Effects and outcomes of interventions on response to VAW during routine SRH consultation plus community engagement

Outcomes	Results	Effect (95% CI)	N of participants (design/follow up)	Study	Risk of bias	Direction of effect/interpretation
**Direct effects**						
**HCP knowledge about VAW and relevant procedures (n=2)**						
Proportion who knew whether there was law that deals with family violence	Pre-intervention = 71%<br>Post-intervention = 90%	19%	?HCPs (UBA/3 years)	Bott 2014[52]	High	Systems level activities, HCP training and ongoing support, infrastructure improvement, integrated family planning-GBV consultation, referral to onsite GBV specialist, community education contributed towards 19% increase in HCP knowledge about legal side of VAW
Proportion who could explain legal obligation of providers regarding family violence	Pre-intervention = 14%<br>Post-intervention = 69%	55%				
Proportion (n) who knew the main objectives of the programme	100% (35/35)		35 HCPs (Cross sectional/4-year service data)	Sithole 2018[41]	High	HCP training, infrastructure improvement, community education on post-rape care contributed towards 25% to 100% HCP awareness about post-rape care
Proportion of doctors who knew the tools to monitor the programme	25% (1/4)					
Proportion of doctors who knew the correct treatment guidelines	25% (1/4)					
Proportion of doctors who did not know the management process	75% (3/4)					
Proportion of nurses who knew the management process	100% (27/27)					
HCP attitudes about VAW (n=1)						
Reduction in proportion who blamed victims of physical and sexual violence (5 indicators), pre-post-intervention	-	By 29% for women provoke physical aggression<br>By 13% for men cannot control their sexual behaviour	?HCPs (UBA/3 years)	Bott 2014[52]	High	Systems level activities, HCP training and ongoing support, infrastructure improvement, integrated family planning-GBV consultation, referral to onsite GBV specialist, community education contributed towards 13-29% reduction in negative attitudes about GBV among HCPs
**HCP readiness for identifying and responding to VAW (n=1)**						
Reduction in proportion of cited 9 barriers to identifying IPV, pre-post intervention	-	By 29% for cultural divide between client and provider<br>By 3% for time constraints	?HCPs (UBA/3 years)	Bott 2014[52]	High	Systems level activities, HCP training and ongoing support, infrastructure improvement, integrated family planning-GBV consultation, referral to onsite GBV specialist, community education contributed 3% to 29% reduction in perceived barriers to identifying GBV among HCPs

For peer review only

Outcomes	Results	Effect (95% CI)	N of participants (design/follow up)	Study	Risk of bias	Direction of effect/interpretation
Increase in proportion who felt prepared to provide counselling about emergency contraception to GBV victims	-	By 96% for counselling about emergency contraception				Systems level activities, HCP training and ongoing support, infrastructure improvement, integrated family planning-GBV consultation, referral to onsite GBV specialist, community education contributed towards 95% increase in HCP preparedness to identify and respond to GBV patients
**Women attitudes about VAW (n=1)**						
Proportion (n) who justified husband physical abuse because of childcare, post-intervention	Intervention = 41.8% (261/625)<br>Control = 45.5% (284/624)	OR = 0.81 (0.60; 1.09)	656 women (cluster RCT/28 months)	Settergren 2018[39]	High	Systems level activities, HCP training, infrastructure improvement, integrated HIV-GBV consultation, onsite and external referral, community and couple education improved 1 out of 5 indicators of women's accepting attitudes towards VAW
Proportion (n) who justified husband physical abuse because she refuses to have sex with her partner, post-intervention	Intervention = 21.0% (131/625)<br>Control = 23.7% (148/624)	OR = 0.65 (0.46; 0.91)				
Mean (SD) score of the Violence domain of the Gender Equitable Men Scale, post-intervention	Intervention = 13.17 (3.98)<br>Control = 12.51 (3.93)	MD = 1.08 (0.52; 1.65)				Systems level activities, HCP training, infrastructure improvement, integrated HIV-GBV consultation, onsite and external referral, community and couple education improved women's attitudes towards more equitable gender roles
Mean (SD) score of the Domestic chores and daily life domain domain of the Gender Equitable Men Scale, post-intervention	Intervention = 8.74 (3.63)<br>Control = 7.62 (3.14)	MD = 1.26 (0.81; 1.71)				
**Intermediate effects**						
**VAW enquiry rate (n=2)**						
Proportion (n) who received GBV screening and counselling, post-intervention	Intervention = 88.5% (1251/1413)<br>Control = 91/7% (442/482)<br>p=0.785	-	656 women (cluster RCT/28 months)	Settergren 2018[39]	High	Systems level activities, HCP training, infrastructure improvement, integrated HIV-GBV consultation, onsite and external referral, community and couple education had no effect on GBV enquiry rate
Proportion (n) who were screened for IPV of those attended clinic	94.5% (171/181)	-	171 women (Cross-sectional/4 months)	Samandari 2016[38]	High	System level activities, 7-day HCP training and ongoing support, integrated family planning-IPV consultation, external referral, community education contributed towards 95% IPV enquiry rate
**Provision of VAW referrals (n=2)**						
Proportion (n) who were referred to safe house and shelter of those screened, post-intervention	Intervention = 12.3% (173/1412)<br>Control = 2.3% (11/488)<br>p=0.216	-	656 women (cluster RCT/28 months) (cluster RCT/28 months)	Settergren 2018[39]	High	Systems level activities, HCP training, infrastructure improvement, integrated HIV-GBV consultation, onsite and external referral, community and couple education

Outcomes	Results	Effect (95% CI)	N of participants (design/follow up)	Study	Risk of bias	Direction of effect/interpretation
						had no effect on rates of referrals to safe house and shelter
Proportion (n) who were signposted to IPV services of those disclosed	100% (157/157)	-	171 women (Cross-sectional/4 months)	Samandari 2016[38]	High	System level activities, 7-day HCP training and ongoing support, integrated family planning-IPV consultation, external referral, community education contributed towards 100% signposting to IPV services
**Provision of safety planning (n=1)**						
Proportion (n) who received safety planning of those disclosed IPV	87.3% (137/157)	-	171 women (Cross-sectional/4 months)	Samandari 2016[38]	High	System level activities, 7-day HCP training and ongoing support, integrated family planning-IPV consultation, external referral, community education contributed towards 87% safety planning rate
**Provision of post-rape care (n=3)**						
Mean number of rape cases presenting to hospital per month.	Pre-intervention = 8<br>Post-intervention = 13	-	334 survivors of sexual assault (UBA and cross-sectional/not reported)	Kim 2007[54]	High	Systems level activities, 2-day HCP training, infrastructure improvement, community education on post-rape care contributed towards increased number of rape cases presenting to hospital
Proportion (n) of eligible patients who received post-rape medical kit	100% (2,081/2,081)	-	13 sites, 2081 patients (Cross-sectional/4-year service data)	Bress 2018[29]	High	HCP training and ongoing support, infrastructure improvement, community education on post-rape care contributed towards 100% provision of post-rape care medical kit
Change in proportion who attended within 72 hours, over 4 years	-	46%	80 HCPs, 1669 patients (Cross-sectional/4 years)	Sithole 2018[41]	High	HCP training, infrastructure improvement, community education on post-rape care contributed towards improvement on 6 indicators of post-rape care provision
Change in proportion who received HIV post-exposure prophylaxis, over 4 years	-	31%				
Change in proportion who received counselling, over 4 years	-	65%				
Change in proportion who received HIV testing, over 4 years	-	96.4%				
Change in proportion who received emergency contraception, over 4 years	-	8%				
Change in proportion who received STI prophylaxis	-	26%				
**VAW disclosure rates (n=2)**						
Proportion who disclosed GBV of those screened	14%	-	? women (UBA/3 years)	Bott 2004[52]	High	Systems level activities, HCP training and ongoing support, infrastructure improvement, integrated family planning-GBV consultation, referral to onsite GBV specialist, community education contributed towards 14% GBV identification rate

For peer review only

Outcomes	Results	Effect (95% CI)	N of participants (design/follow up)	Study	Risk of bias	Direction of effect/interpretation
Proportion (n) who disclosed IPV of those screened	91.8% (157/171)	-	171 women (Cross-sectional/4 months)	Samandari 2016[38]	High	System level activities, 7-day HCP training and ongoing support, integrated family planning-IPV consultation, external referral, community education contributed towards 92% IPV identification rate
Proportion (n) who disclosed GBV of those screened	37% (49/134)	-	134 women (Cross-sectional/5 months)	Turan 2013[43]	High	40-hour HCP training, integrated ANC-GBV consultation, assisted external referral, community education contributed towards 37% IPV identification rate
**VAW referrals uptake (n=2)**						
Proportion who took referral of those disclosed GBV	30%	-	? women (UBA/3 years)	Bott 2004[52]	High	Systems level activities, HCP training and ongoing support, infrastructure improvement, integrated family planning-GBV consultation, referral to onsite GBV specialist, community education contributed towards 30% uptake of referrals
Proportion (n) who took referral of those disclosed IPV	0.6% (1/157)	-	171 women (Cross-sectional/4 months)	Samandari 2016[38]	High	System level activities, 7-day HCP training and ongoing support, integrated family planning-IPV consultation, external referral, community education contributed towards 0.6% uptake of external referrals
Proportion (n) who took referral of those disclosed GBV	53% (26/49)	-	134 women (Cross-sectional/5 months)	Turan 2013[43]	High	40-hour HCP training, integrated ANC-GBV consultation, assisted external referral, community education contributed towards 53% uptake of referrals
**Use of SRH services (n=1)**						
Proportion (n) who attended any antenatal care, post-intervention	Intervention = 88.7% (1597/1800)<br>Control = 82.4% (1526/1851)	RD = 0.063 (-0.044; 0.170)	1837 women (cluster RCT/12 months)	Cockcroft 2019[32]	Low	HCP training, infrastructure improvement, integrated DV-universal home visits that discussed domestic violence, heavy work in pregnancy, ignorance of danger signs, and lack of spousal communication with pregnant women and their spouses had no effect on 8 indicators of use of antenatal care, institutional delivery, or skilled birth attendance
Proportion (n) who delivered in a health facility, post-intervention	Intervention = 30.1% (475/1579)<br>Control = 21.9 (391/1785)	RD = 0.082 (-0.071; 0.235)				
Proportion (n) who delivered by a skilled health worker post-intervention	Intervention = 29.3 (463/1579)<br>Control = 22.7 (404/1783)	RD = 0.067 (-0.081; 0.214)				
Mean (SD of GBV client visits per facility, post-intervention	Intervention = 237.8 (110.58)<br>Control = 81.5 (46.09)<br>p=0.010	-	656 women (cluster RCT/28 months)	Settergren 2018[39]	High	Systems level activities, HCP training, infrastructure improvement, integrated HIV-GBV consultation, onsite and external referral, community and couple education

For peer review only

Outcomes	Results	Effect (95% CI)	N of participants (design/follow up)	Study	Risk of bias	Direction of effect/interpretation
						increased use of SRH services by women with experience of GBV
Proportion (n) of GBV clients who received HIV testing and counselling, post-intervention	Intervention = 73.3% (1038/1416)<br>Control = 20.9% (102/488)<br>p=<0.001	-				Systems level activities, HCP training, infrastructure improvement, integrated HIV-GBV consultation, onsite and external referral, community and couple education increased use of 4 (HIV testing and counselling, family planning, forensic exam) out of 24 SRH services
Proportion (n) of GBV clients who received STI test, post-intervention	Intervention = 21.8% (308/1415)<br>Control = 11.5% (56/488)<br>p=0.128	-				
Safety behaviour (n=1)						
Proportion (n) of women who used condom, post-intervention	Intervention = 17.0% (157/931)<br>Control = 16.0% (192/1170)	PRR = 1.03 (0.85; 1.25)<br>aPRR = 1.01 (0.84; 1.21)	6 facilities, 1812 women (cluster RCT/35 months)	Wagman 2015[45]	High	HCP training, integrated HIV-IPV consultation, onsite referral, community education had no effect on 6 indicators of risk behaviours among women
**Outcomes**						
**Re-exposure to VAW (n=3)**						
Proportion (n) who did not experience physical domestic violence during pregnancy, post-intervention	Intervention = 97.4 (1772/1820)<br>Control = 90.9 (1677/1844)	RD = 0.064 (0.045; 0.084)	1837 women (RCT/12 months)	Cockcroft 2019[32]	Low	HCP training, infrastructure improvement, integrated DV-universal home visits that discussed domestic violence, heavy work in pregnancy, ignorance of danger signs, and lack of spousal communication with pregnant women and their spouses reduced physical domestic violence
Proportion (n) who experienced any IPV in past 12 months, post-intervention	Intervention = 37.2% (207/556)<br>Control = 45.7% (268/587)	OR = 0.85 (0.62; 1.16)	6 facilities, 735 households (cluster RCT/28 months)	Settergren 2018[39]	High	Systems level activities, HCP training, infrastructure improvement, integrated HIV-GBV consultation, onsite and external referral, community and couple education had no effect on any IPV
Proportion (n) who experienced physical IPV in past 12 months, post-intervention	Intervention = 12% (217/1812)<br>Control = 16% (346/2127)	PRR = 0.74 (0.63; 0.86)<br>aPRR = 0.79 (0.67; 0.92)	6 facilities, 1812 women (cluster RCT/35 months)	Wagman 2015[45]	High	HCP training, integrated HIV-IPV consultation, onsite referral, community education reduced incidents of physical and sexual IPV and had no effect on emotional IPV.
Proportion (n) who experienced emotional IPV in past 12 months, post-intervention	Intervention = 18% (311/1737)<br>Control = 20% (409/2039)	PRR = 0.89 (0.78; 1.02)<br>aPRR = 0.91 (0.79; 1.04)				
Proportion (n) who experienced sexual IPV in past 12 months, post-intervention	Intervention = 10% (167/1737)<br>Control = 13% (261/2038)	PRR = 0.75 (0.62; 0.90)<br>aPRR = 0.80 (0.67; 0.97)				
**Sexual and reproductive health (n=1)**						

For peer review only

Outcomes	Results	Effect (95% CI)	N of participants (design/follow up)	Study	Risk of bias	Direction of effect/interpretation
Proportion (n) who did not have swelling of face or hands, post-intervention	Intervention = 97.4% (1790/1837)<br>Control = 71.1% (1317/1853)	RD = 0.264 (0.194; 0.333)	1837women (RCT/12 months)	Cockcroft 2019[32]	Low	HCP training, infrastructure improvement, integrated DV-universal home visits that discussed domestic violence, heavy work in pregnancy, ignorance of danger signs, and lack of spousal communication with pregnant women and their spouses improved 9 out of 13 indicators of pregnancy and postpartum complications
Proportion (n) who did not have raised blood pressure, post-intervention	Intervention = 96.6% (1409/1458)<br>Control = 85.1% (1269/1492)	RD = 0.116 (0.042; 0.190)				
Proportion (n) who did not have post-partum sepsis, post-intervention	Intervention = 81.1% (1478/1822)<br>Control = 48.8% (903/1852)	RD = 0.324 (95% CI 0.115; 0.493)				
Incidence of HIV per100 person-years	Intervention = 0.99<br>Control = 1.15	IRR = 0.86 (0.61; 1.22<br>aIRR = 0.72 (0.49; 1.07))	6 facilities, 1812 women (cluster RCT/35 months)	Wagman 2015[45]	High	HCP training, integrated HIV-IPV consultation, onsite referral, community education had no effect on incidence of HIV among women

Note. VAW violence against women. GBV gender-based violence. DV domestic violence. IPV intimate partner violence. SRH sexual and reproductive health. STI Sexually transmitted infections. HIV human immunodeficiency virus. ANC antenatal care. HCP health care provider. RCT randomised controlled trial. CBA controlled before-after study. UBA uncontrolled before-after study. CI confidence interval. SD standard deviation. IQR interquartile range. MD mean difference. OR odds ratio. RD risk difference. IRR incidence rate ratio.

For peer review only

Supplementary file 6. Effects and outcomes of interventions on response to VAW in addition to routine SRH consultation

Outcomes	Results	Effect (95% CI)	N of participants (design/follow up)	Study	Risk of bias	Direction of effect/interpretation
**Direct effects**						
**Women knowledge about VAW (n=1)**						
Proportion who recognised violence as an issue of power, post-intervention	60.6%		155 women (UBA/ First prenatal appointment to 6 weeks after delivery)	Arora 2019[49]	High	After 2 or more 30-45-minute psychosocial counselling sessions, around 60% of pregnant women were aware about domestic violence and its impact on health
Proportion who recognised the impact of violence on health, post-intervention	65.5%					
**Women readiness for addressing VAW (n=1)**						
Proportion who recognised the need to take steps to address violence, post intervention	59.9%		155 women (UBA/ First prenatal appointment to 6 weeks after delivery)	Arora 2019[49]	High	After 2 or more 30-45-minute psychosocial counselling sessions, 60% of pregnant women were ready to address VAW
**Intermediate effects**						
**VAW referral uptake (n=1)**						
Proportion (n) who used specialist IPV services, post-intervention	Intervention = 0.96% (1/104)<br>Control = 1.00% (1/100)	-	110 women (RCT/Prenatal appointment to 1 week after delivery)	Cripe 2010[53]	Some concerns	1 30-minute psychosocial counselling session, resource card, external referral had no effect on uptake of external referrals
**Use of SRH services (n=1)**						
Proportion (n) who missed antiretroviral medication, post-intervention	Intervention = 42.3% (19)<br>Control = 36.4% (25)	-	32 women (RCT/6 months)	Sikkema 2018[40]	High	7 90-minute psychosocial sessions on coping had no effect on engagement with HIV treatment among women with a history of sexual violence
Proportion (n) with high levels of non-retention in care, post-intervention	Intervention = 42.3% (26)<br>Control = 33.3% (27)	-				
**Use of non-health services (n=1)**						
Proportion (n) who used legal services, post-intervention	Intervention = 1.92% (2/104)<br>Control = 3.00% (3/100)	-	110 women (RCT/Prenatal appointment to 1 week after delivery)	Cripe 2010[53]	Some concerns	1 30-minute psychosocial counselling session, resource card, external referral had no effect on use of community resources among pregnant women
Proportion (n) who used police, post-intervention	Intervention = 0.96% (1/104)<br>Control = 4.00% (4/100)	-				
Proportion (n) who used social services, post-intervention	Intervention = 1.92% (2/104)<br>Control = 2.00% (2/100)	-				
**Safety behaviour (n=4)**						
Mean (SD) score of using safety behaviours, post-intervention	Intervention = 9.50 (2.63)<br>Control = 7.74 (2.42)	MD = 2.41 (1.43; 3.40)	70 women (RCT/Prenatal appointment to 6 weeks after delivery)	Sapkota 2020[51]	Low	1 35-45-minute psychosocial counselling session, resource card, contact details of the counsellor increased use of safety behaviours among pregnant women

For peer review only

Outcomes	Results	Effect (95% CI)	N of participants (design/follow up)	Study	Risk of bias	Direction of effect/interpretation
Proportion who adopted safety behaviours, post-intervention	Intervention = 30.3%<br>Control = 11.2%		110 women (RCT/Prenatal appointment to 1 week after delivery)	Cripe 2010[53]	Some concerns	1 30-minute psychosocial counselling session, resource card, external referral had no effect on safety behaviours among pregnant women
Mean (SD) score of avoidance, coping post-intervention	Intervention = 2.17 (0.13)<br>Control = 1.99 (0.09)	-	32 women (RCT/6 months)	Sikkema 2018[40]	High	7 90-minute psychosocial training sessions reduced avoidance coping, but had no effect on social coping among women with a history of sexual violence
Mean (SD) score of social coping, post-intervention	Intervention = 2.90 (0.10)<br>Control = 2.58 (0.10)	-				
Proportion (n) who used adaptive coping strategies at individual level pre- and post-intervention	Pre-intervention = 51.4% (73)<br>Post-intervention = 59.1% (84)<br>p=0.193		155 women (UBA/ First prenatal appointment to 6 weeks after delivery)	Arora 2019[49]	High	2 or more 30-45-minute psychosocial counselling sessions had no effect on coping behaviours among pregnant women
Proportion who used adaptive coping strategies at informal and formal levels pre- and post-intervention	Pre-intervention = 85.2% (121)<br>Post-intervention = 86.6% (123)<br>p=0.832					
**Outcomes**						
**Re-exposure to VAW (n=4)**						
Mean (SD) score of verbal and physical IPV, post-intervention	Intervention = 11.62 (2.05)<br>Control = 13.28 (1.94)<br>p<0.001	-	50 pregnant women (RCT/2 months)	Khalili 2019[46]	High	4 90-minute psychoeducational counselling sessions reduced verbal and physical IPV among pregnant women
Mean (SD) score of total IPV, post-intervention	Intervention = 17.70 (11.12)<br>Control = 31.22 (21.17)	MD = 13.51 (9.99; 17.02)	141 pregnant women (RCT/6 months)	Mutisya 2018[37]	High	1-3 30-35-minute psychosocial counselling sessions, risk assessment, safety planning, resource card, external referral reduced IPV among pregnant women
Proportion who experienced physical IPV, post intervention	Intervention = 51.2%<br>Control = 65.9%	RR = 0.78 (0.63; 0.93)	125 women (RCT/3 months)	Tanghizaden 2018[47]	Some concerns	4 90-minute psychosocial training sessions on problem-solving skills reduced physical and psychological IPV but had no effect on sexual IPV among pregnant women
Proportion who experienced psychological IPV, post intervention	Intervention = 67.4%<br>Control = 92.4%	RR = 0.73 (0.64; 0.83)				
Proportion who experienced sexual IPV, post intervention	Intervention = 50.4%<br>Control = 57.6%	RR = 0.87 (0.69; 1.09)				
Change in proportion who experienced physical domestic violence, before-after	74.6% to 3.5%	-	155 women (UBA/ First prenatal appointment to 6 weeks after delivery)	Arora 2019[49]	High	2 or more 30-45-minute psychosocial counselling sessions reduced physical, emotional, and financial domestic violence among pregnant women

Outcomes	Results	Effect (95% CI)	N of participants (design/follow up)	Study	Risk of bias	Direction of effect/interpretation
Change in proportion who experienced emotional domestic violence, before-after	98.6% to 34.5%	-				
Change in proportion who experienced financial domestic violence, before-after	72.5% to 11.3%	-				
**Mental health (n=6)**						
Mean (SD) score of anxiety, post-intervention	Intervention = 4.33 (3.84)<br>Control = 6.93 (4.87)	MD = -3.73 (-5.42; -2.04)	70 women (RCT/prenatal appointment to 6 weeks after delivery)	Sapkota 2020[51]	Low	1 35-45-minute psychosocial counselling session, resource card, contact details of the counsellor reduced anxiety and depression among pregnant women
Mean (SD) score of depression, post-intervention	Intervention = 3.51 (3.46)<br>Control = 6.13 (3.68)	MD = -3.41 (-4.84; -1.99)				
Mean (SD) score of postnatal depression, post-intervention	Intervention = 5.34 (4.23)<br>Control = 12.46 (4.22)	MD = 7.12 (6.21; 8.03)	141 women (RCT/6 months)	Mutisya 2018[37]	High	1-3 30-35-minute psychosocial counselling sessions with risk assessment, safety planning, external referral, and resource card reduced depression among pregnant women
Mean (SD) score of PTSD, post-intervention	Intervention = 28.61 (5.04)<br>Control = 22.50 (3.47)	-	32 women (RCT/6 months)	Sikkema 2018[40]	High	7 90-minute psychosocial training sessions had no effect on PTSD symptoms among women with a history of sexual violence
Mean (SD) score of psychological distress, post-intervention	Intervention = 22.28 (3.81)<br>Control = 24.06 (4.16)<br>$p<0.001$	-	50 women (RCT/2 months)	Khalili 2019[46]	High	4 90-minute psychoeducational counselling sessions reduced psychological distress among pregnant women
Difference between baseline and post-intervention mean (SD) score for mental health	Intervention = 2.50 (20.95)<br>Control = 2.04 (19.61)	MD = 4.54 (-1.07; 10.15)	110 women (RCT/Prenatal appointment to 1 week after delivery)	Cripe 2010[53]	Some concerns	1 30-minute psychosocial counselling session, resource card, external referral had no effect on mental health of pregnant women
Change in proportion who experienced any emotional health problems	96.5% to 33.1%	-	155 women (UBA/ First prenatal appointment to 6 weeks after delivery)	Arora 2019[49]	High	2 or more 30-45-minute psychosocial counselling sessions reduced % of pregnant women with emotional health problems.
**Sexual and reproductive health (n=1)**						
Proportion (n) of those with unsuppressed HIV viral load, post-intervention	Intervention = 15.8% (19)<br>Control = 20.0% (25)<br>($\chi 2$ (1) = 0.13, p = 0.72)	-	32 women (RCT/6 months)	Sikkema 2018[40]	High	7 90-minute psychosocial training sessions had no effect on adherence to therapy measured by HIV viral load among women with a history of sexual abuse
**Physical health (n=2)**						
Difference between baseline and post-intervention mean (SD) score for general health	Intervention = 5.30 (15.62)<br>Control = 4.74 (14.67)	MD = 0.05 (-6.80; 7.79)	110 women (RCT/Prenatal appointment to 1 week after delivery)	Cripe 2010[53]	Some concerns	1 30-minute psychosocial counselling session, resource card, external referral had no effect on general health

For peer review only

Outcomes	Results	Effect (95% CI)	N of participants (design/follow up)	Study	Risk of bias	Direction of effect/interpretation
Change in proportion who experienced any physical health problems	54.6% to 10.5%	-	155 women (UBA/ First prenatal appointment to 6 weeks after delivery)	Arora 2019[49]	High	2 or more individual psychosocial counselling sessions reduced % of pregnant women with physical health problems.
**Quality of life (n=2)**						
Mean (SD) score of overall quality of life, post-intervention	Intervention = 17.22 (3.00)<br>Control = 15.19 (2.77)	MD = 2.45 (1.51; 3.39)	70 women (RCT/prenatal appointment to 6 weeks after delivery)	Sapkota 2020[51]	Low	1 35-45-minute psychosocial counselling session, resource card, contact details of the counsellor improved quality of life among pregnant women
Difference between baseline and post-intervention mean (SD) score for physical functioning	Intervention = -15.67 (28.35)<br>Control = -15.70 (25.06)	MD = 0.03 (-7.37; 7.42)	110 women (RCT/Prenatal appointment to 1 week after delivery)	Cripe et 2010[53]	Some concerns	1 30-minute psychosocial counselling session, resource card, external referral had no effect on general health, bodily pain, vitality, social functioning among pregnant women.
Difference between baseline and post-intervention mean (SD) score for bodily pain	Intervention = -7.40 (28.33)<br>Control = -7.90 (24.28)	MD = 0.50 (-6.80; 7.79)				
Difference between baseline and post-intervention mean (SD) score for vitality	Intervention = -0.19 (22.34)<br>Control = -3.65 (22.06)	MD = 3.46 (-2.67; 9.59)				
Difference between baseline and post-intervention mean (SD) score for social functioning	Intervention = -0.36 (34.94)<br>Control = -3.50 (37.06)	MD = 3.14 (-6.80; 13.08)				

Note. VAW violence against women. GBV gender-based violence. DV domestic violence. IPV intimate partner violence. SRH sexual and reproductive health. STI Sexually transmitted infections. HIV human immunodeficiency virus. ANC antenatal care. HCP health care provider. RCT randomised controlled trial. CBA controlled before-after study. UBA uncontrolled before-after study. CI confidence interval. SD standard deviation. IQR interquartile range. MD mean difference. RR relative risk.

For peer review only

Supplementary file 7. Barriers to identification and response to VAW in sexual and reproductive health services

Theme	Description	Discussed by	Supporting text
Acceptability of VAW	Attitudes and social norms that regulate the acceptability of VAW at individual and community levels	Cristofides 2010,[31] Laisser 2011,[35] Smith 2013,[42] Turan 2013[43]	**Women:** "Women did not want a referral because they did not feel the violence was serious or they felt these were personal issues that they will solve on their own"[33] **HCPs:** "Some of these patients are themselves to be blamed. You know some women don't want to be polite to their husbands and adhere to the norms in their marriages that is why they are beaten. It takes time, need to be more patient and expertize to screen, which we miss. It may be too costly for training. (FGD3 Male Nurse)"[35] **Community:** "So when somebody is saying that women are not supposed to be beaten, that. . . they should go to somebody and take some action, in the community it is like that person is acting against the will of the community. To the men it is like he is an outcast in the community, an outlaw who is not supposed to be there. . . . In social places you will hear them saying that he is not a good person because if he is preaching to our ladies and women to take action against us, then it is like he wants to bring a revolution, women are going to overpower us and then we are going to be voiceless. (Focus Group #1, Respondent #8)"[43]
Fear of negative consequences	Real or potential negative consequences (psychological, legal, financial) of engaging in VAW work that could make the situation worse for individuals and healthcare system	Bott 2004,[52] Christofides 2010,[31] Haberland 2016,[33] Knettel 2019[55] Laisser 2011,[35] Samandari 2016,[38] Sapkota 2020,[56] Sithole 2018[41]	**Women:** "The sessions would irritate me when we talked about my rape; I hated to talk about it even though when I had talked about it, I would feel better. My heart would feel sore. Even talking about my HIV status irritated me because I still beat myself for infecting my child"[55] "a few women stated that privacy concerns made it difficult for them to participate in the intervention, especially the group sessions. One participant explained that she was "afraid that I might be seen by a participant who knows me and who might go around discussing my problems."[55] **HCPs:** "Providers were responsible for all IPV screening and counseling, as well as their regular FP duties. This led not only to an increased burden of duty for providers, but also the experience of secondary trauma, resulting from the exposure to clients' IPV stories."[38] **Healthcare system**: "In one shift we normally attend up to 60 plus in a room for the two clinicians. Sometimes we reach up to 100 clients when it is a busy day, but if we are to attend one client at a time then it will be only 15 clients per day in a room. Where will others go?" Male clinician"[35]
Limited readiness for VAW work in SRH services	Structural unreadiness within healthcare system: lack of support from leadership, time pressure, insufficient budget, lack of adequate resources, limited privacy	Abeid 2016,[27] Bott 2004,[52] Christofides 2010,[31] Haberland 2016,[33] Laisser 2011,[35] Sammandari 2016,[38] Undie 2016[44]	"System level factors may have influenced the implementation of IPV screening by lay counsellors …Other factors included inadequate management and supervision, burn-out, and small stipends which adversely affect counsellors' motivation to do something perceived as extra."[31] "The HCWs felt they had not much to offer to the women who were experiencing IPV. This category thus represents an uncertainty as to whether the health care system is ready for routine screening for IPV and suggests a need for reinforced organizational change."[35] "Perceived barriers to replication and scale-up included inadequate funding, insufficient clinic staff, and lack of political commitment for IPV services on the part of MOHPH"[38] "Providers' main criticism was the longer time required to conduct the enhanced counseling. It created delays in the system, frustrating clients who were tired of being at the hospital for so long. Providers also felt the effects of extra time. One noted explicitly that they are supposed to see a certain number of clients each day and if they do not meet their targets they will have problems with management."[33] "D3: It is also difficult to examine a patient in front of another one even if we use curtains. There is one examination bed for two of us and when you ask questions about STD patients feel embarrassed. Although we try to use low voice, people like to listen to others' conversations. (FGD1 Female Clinician"[35]
	Wider systems unreadiness: lack of services to refer to, poor referral system, untrained staff in non-health services	Abeid 2016,[27] Bott 2004,[52] Christofides 2010,[31] Laisser 2011[35]	"Providing referrals to women who disclosed current experiences of IPV may be of limited utility where services are hard to access"[31]

For peer review only

	Society unreadiness: poverty, no money for transport fare and healthcare services, no transport, financial dependence on husband.	Haberland 2016,[33] Knettel 2019,[55] Laisser 2011,[35] Sithole 2018[41]	"D1: You know I have nothing much to say but would like to do the screening -the resources are my dilemma. Many women are poor 'wanyonge' and are not strong enough to fight with their husbands but maybe this would be their good start. They will be happier later in future. (FGD3 Female Clinician)"[35] "Her issue was that the partner used to beat her and to abuse her physically when she asked for bus fare to come to the clinic. So you see at the end of the day if she doesn't get help to deal with the violence she won't be able to come to the clinic because she is being abused when she asks for money to come to the clinic and it will affect her overall outcome…"[33] "Getting to the clinic would be a challenge as I do not work and often had to borrow money."[55]
	Women's unreadiness for VAW services offered by HCPs: the demand-supply gap between women's preferences for adequate response to VAW and what HCPs offered to them	Christofides 2010,[31] Haberland 2016,[33] Undie 2016[44]	"However, one woman questioned whether there was any point in talking to the lay counselor unless the counselor would go home and make her husband stop. Other participants, who had not disclosed abuse, suggested that health care providers could talk to a woman's abusive partner and this would stop the violence. Others suggested that if abusive partners knew about IPV screening they would stop. This seems perhaps unrealistic."[31]
			"Although the initial intention of the intervention was for IPV-positive clients to receive same-day services at the GBV clinic, this was not always possible, because women did not have the time, GBV clinic staff were not always available, and some clients preferred to have their initial GBV clinic appointment on a later date. There were occasions when providers referred clients to the GBV clinic and the clients initially complied with the referral, only to find that their needs could not be attended to immediately due to the unavailability of staff atthe GBV clinic."[44]

For peer review only

BMJ Open

INTERVENTIONS IN SEXUAL AND REPRODUCTIVE HEALTH SERVICES ADDRESSING VIOLENCE AGAINST WOMEN IN LOW- AND MIDDLE- INCOME COUNTRIES: A MIXED-METHODS SYSTEMATIC REVIEW

Journal:	*BMJ Open*
Manuscript ID	bmjopen-2021-051924.R1
Article Type:	Original research
Date Submitted by the Author:	20-Oct-2021

Complete List of Authors:	Lewis, Natalia; University of Bristol, Bristol Medical School (PHS)<br>Munas, Muzrif; University of Peradeniya; University of Bristol Faculty of Health Sciences, Bristol Medical School (PHS)<br>Colombini, Manuela; London School of Hygiene and Tropical Medicine, Department of Global Health and Development<br>D'Oliveira, AF; University of São Paulo Institute of Biomedical Sciences, Faculty of Medicine<br>Pereira, Stephanie ; University of São Paulo Institute of Biomedical Sciences, Faculty of Medicine<br>Shrestha, Satya; Kathmandu University School of Medical Sciences; University of Bristol<br>Rajapakse, Thilini; University of Peradeniya<br>Shaheen, Amira ; An-najah National University Faculty of Medicine and Health Sciences<br>Rishal , Poonam; Kathmandu University School of Medical Sciences<br>Alkaiyat, Abdulsalam ; An-najah National University Faculty of Medicine and Health Sciences<br>Richards, Alison; University of Bristol, Bristol Medical School (PHS); NIHR ARC West, University Hospitals Bristol NHS Foundation Trust<br>Garcia-Moreno, Claudia; Organisation mondiale de la Sante, Department of Reproductive Health and Research<br>Feder, Gene; University of Bristol Faculty of Health Sciences, Centre for Academic Primary Care<br>Bacchus, Loraine; London School of Hygiene and Tropical Medicine Faculty of Public Health and Policy

**Primary Subject Heading**:	Global health
Secondary Subject Heading:	Health services research, Sexual health
Keywords:	PUBLIC HEALTH, REPRODUCTIVE MEDICINE, SEXUAL MEDICINE, Organisation of health services < HEALTH SERVICES ADMINISTRATION & MANAGEMENT, OBSTETRICS

SCHOLARONE™
Manuscripts

I, the Submitting Author has the right to grant and does grant on behalf of all authors of the Work (as defined in the below author licence), an exclusive licence and/or a non-exclusive licence for contributions from authors who are: i) UK Crown employees; ii) where BMJ has agreed a CC-BY licence shall apply, and/or iii) in accordance with the terms applicable for US Federal Government officers or employees acting as part of their official duties; on a worldwide, perpetual, irrevocable, royalty-free basis to BMJ Publishing Group Ltd ("BMJ") its licensees and where the relevant Journal is co-owned by BMJ to the co-owners of the Journal, to publish the Work in this journal and any other BMJ products and to exploit all rights, as set out in our licence.

The Submitting Author accepts and understands that any supply made under these terms is made by BMJ to the Submitting Author unless you are acting as an employee on behalf of your employer or a postgraduate student of an affiliated institution which is paying any applicable article publishing charge ("APC") for Open Access articles. Where the Submitting Author wishes to make the Work available on an Open Access basis (and intends to pay the relevant APC), the terms of reuse of such Open Access shall be governed by a Creative Commons licence – details of these licences and which Creative Commons licence will apply to this Work are set out in our licence referred to above.

Other than as permitted in any relevant BMJ Author's Self Archiving Policies, I confirm this Work has not been accepted for publication elsewhere, is not being considered for publication elsewhere and does not duplicate material already published. I confirm all authors consent to publication of this Work and authorise the granting of this licence.

For peer review only

INTERVENTIONS IN SEXUAL AND REPRODUCTIVE HEALTH SERVICES ADDRESSING VIOLENCE AGAINST WOMEN IN LOW- AND MIDDLE- INCOME COUNTRIES: A MIXED-METHODS SYSTEMATIC REVIEW

Natalia V Lewis,[1] Muzrif Munas,[1,2] Manuela Colombini,[3] Ana Flavia d'Oliveira,[4] Stephanie Pereira,[4] Satya Shrestha,[1,5] Thilini Rajapakse,[2] Amira Shaheen,[6] Poonam Rishal,[5] Abdulsalam Alkaiyat,[6] Alison Richards,[1,7] Claudia M García-Moreno,[8] Gene S Feder,[1] Loraine J Bacchus[3]

[1]Centre for Academic Primary Care, Population Health Sciences, Bristol Medical School, University of Bristol, UK

[2]University of Peradeniya, Faculty of Medicine, University of Peradeniya, Sri Lanka

[3]Department of Global Health and Development, Faculty of Public Health and Policy, London School of Hygiene & Tropical Medicine, UK

[4]Faculty of Medicine, University of São Paulo, Brazil

[5]School of Medical Sciences, Kathmandu University, Nepal

[6]Public Health Department, Faculty of Medicine and Health Sciences, An-Najah National University, Nablus, Occupied Palestinian Territories

[7]The National Institute for Health Research Applied Research Collaboration West (NIHR ARC West) at University Hospitals Bristol and Weston NHS Foundation Trust, UK

[8]Department of Sexual and Reproductive Health and Research, World Health Organisation

Correspondence to: Dr Natalia V Lewis, nat.lewis@bristol.ac.uk

Word count: 5779

For peer review only

Key words: domestic violence, intimate partner violence, violence against women, developing countries, low- and middle-income countries, systematic review, reproductive health services.

ABSTRACT

Objectives. To synthesise evidence on the effectiveness, cost-effectiveness, and barriers to responding to violence against women (VAW) in sexual and reproductive health (SRH) services in low- and middle-income countries (LMICs).

Design. Mixed-methods systematic review.

Data sources. Medline, Embase, Psycinfo, Cochrane, Cinahl, IMEMR, Web of Science, Popline, Lilacs, WHO RHL, ClinicalTrials.gov, Google, Google Scholar, websites of key organisations through December 2019.

Eligibility criteria. Studies of any design that evaluated VAW interventions in SRH services in LMICs.

Data extraction and synthesis. Concurrent narrative quantitative and thematic qualitative syntheses, integration through line of argument and mapping onto a logic model. Two reviewers extracted data and appraised quality.

Results. 26 studies of varied interventions using heterogeneous outcomes. Of ten interventions that strengthened health systems capacity to respond to VAW during routine SRH consultation, three reported no harm and reduction in some types of violence. Of nine interventions that strengthened health systems and communities' capacity to respond to VAW, three reported conflicting effects on re-exposure to some types of VAW and mixed effect on SRH. The interventions increased identification of VAW but had no effect on the provision (75-100%) and uptake (0.6-53%) of referrals to VAW services. Of seven psychosocial interventions in addition to SRH consultation that strengthened women's readiness to address VAW, four reduced re-exposure to some types of VAW and improved health. Factors that disrupted the pathway to better outcomes included accepting attitudes towards VAW, fear of consequences, and limited readiness of the society, health systems, and individuals. No study evaluated cost-effectiveness.

Conclusions. Some VAW interventions in SRH services reduced re-exposure to some types of VAW and improved some health outcomes in single studies. Future interventions should strengthen capacity to address VAW across health systems, communities, and individual women. First-line support should be better tailored to women's needs and expectations.

PROSPERO protocol CRD42019137167.

STRENGTHS AND LIMITATIONS OF THIS STUDY

For peer review only

- This review was carried out by a team of researchers from the UK and low- and middle-income countries with expertise and experience in health system responses to violence against women and global health.
- Inclusion of peer-reviewed and grey reports of studies of any design resulted in selection of the most relevant studies.
- The logic model approach to the integration of synthesis findings produced evidence in a format understandable to the end-users of this review.
- Most included studies had methodological limitations and high risk of bias.
- We could not perform meta-analysis of quantitative findings because primary studies evaluated varied interventions and used different instruments to measure varied outcomes.

INTRODUCTION

Violence against women (VAW) is a violation of global health and human rights.[1] The most common forms of VAW are intimate partner violence (IPV) and non-partner sexual violence (NPSV). One in three women worldwide have experienced physical and/or sexual violence, mostly by an intimate partner. VAW is more prevalent in low- and middle-income countries (LMICs). Exposure to VAW is associated with mental and physical health problems, including increased sexually transmitted infection (STI) and HIV, unplanned pregnancy and abortion, gynaecological conditions.[2 3] Although IPV against men is increasingly recognised within the context of both same sex and heterosexual relationships, understanding of male victimisation and its health consequences is still poorly understood[4] and there is a dearth of primary research on the healthcare response to male victims and perpetrators.[5]

The healthcare system has a key role in preventing VAW because most women attend sexual and reproductive health (SRH) services at some point.[6 7] The main role of the healthcare system is to contribute towards secondary and tertiary prevention through early detection of VAW and mitigation of its impact which can prevent ill health and reoccurrence of violence. Healthcare providers (HCPs) are uniquely placed to identify victims/survivors, provide first-line support and clinical care, and connect them with other services. Healthcare systems can also contribute to primary prevention through early identification of children exposed to violence in the home and support to programmes like home visiting or early childhood development.[8] The capacity of healthcare systems to respond to VAW is defined as the cumulative availability and strength of the following *building blocks* from the Health Systems Wheel: (i) leadership and governance, (ii) multi-sectoral coordination, (iii) workforce development, (iv) healthcare delivery, (v) infrastructure, (vi) financing, (vii) monitoring and evaluation (WHO 2010).[9 10]The Health Systems Wheel[11] highlights key components that need to be in place to support individual HCPs and organisations to offer a comprehensive and client-centred response to VAW. It assumes that all elements of the health system – individual, organisational,

For peer review only

contextual, and structural – impact on provision of response to VAW. The WHO guidelines for evidence-based health systems response to VAW adopted the Health Systems Wheel framework to recommend intervention activities across the health systems *building blocks*.[12 13] In LMICs, healthcare delivery for VAW has been implemented through integration at the level of individual HCPs, healthcare facility, and healthcare system.[14]

Systematic reviews[15 16] and WHO guidelines[17] found scant evidence from LMICs on effectiveness of VAW interventions in healthcare. This study addresses the gap by answering two questions: (i) What is the evidence for effectiveness and cost-effectiveness of interventions in SRH services that address VAW? (ii) What are the barriers to the effectiveness?

METHODS

We conducted concurrent quantitative and qualitative evidence syntheses with integration into a line of argument[18] and mapping onto a logic model.[19] The mixed-methods design allowed integration of diverse types of evidence to inform VAW research and intervention development in LMICs. Our analysis was informed by the WHO Health Systems Wheel framework for responding to VAW.[8] We defined the health systems capacity to respond to VAW as the cumulative availability and strength of the Health Systems Wheel *building blocks*. We looked at the capacity of the health systems at three levels: individual providers (e.g., attitudes, knowledge, confidence, behaviour, and practices), services and organisations (e.g., infrastructure, availability of supplies/medicines), community (attitudes, knowledge, practices).[13] We defined women's capacity to respond to VAW as their readiness and ability to seek help, disclose abuse, get referrals, and receive services. We followed the Cochrane[20] and PRISMA guidelines.[21] The study protocol was registered with PROSPERO (CRD42019137167).

Search strategy and selection criteria

We included primary intervention studies reported in any language with an English abstract published since 2005, the year of the first published evaluation of VAW interventions in SRH services (expert opinion from the study advisory group). We identified earlier studies through reference checking. We used terminology and definitions from WHO guidance on strengthening health systems to respond to VAW (Table 1).[13]

Table1. Study inclusion and exclusion criteria with justification

	Inclusion criteria	Exclusion criteria
Participants	Recipients of healthcare services - women of reproductive age (15-49 years old)<br>AND/OR<br>Healthcare providers – organisations (e.g., hospital, clinic, primary care centre, other service delivery points) or individuals (e.g., health care professional, community health worker, or any other person who is trained to deliver healthcare in their community).<br>Studies which recruited only a subset of recipients or providers of healthcare services.	Female children and girls under 15 years old.<br>While recognizing that pregnancies occur among young adolescents 10-14, most studies consider women aged 15-49 years as the main group using SRH services in LMICs.<br>Male recipients of healthcare services.

For peer review only

	Inclusion criteria	Exclusion criteria
Interventions	Any intervention addressing violence against women (VAW). These are complex interventions aimed to identify women affected by violence, provide first-line support, clinical care, and signpost, or refer to available community support services including specialist VAW services. Any definition of VAW, including any type of IPV, domestic violence and abuse, family violence or non-partner sexual violence against a woman, including transgender women.	No intervention Hypothetical intervention addressing VAW. We are synthesising evidence of interventions that have been tested. Female genital mutilation/cutting, trafficking. These type of VAW were addressed in recent systematic reviews. 'Honour'-based violence, forced marriage. There is an overlap between IPV, domestic violence and abuse and 'honour'-based violence and forced marriage. Therefore, we will capture relevant studies through including papers on IPV and domestic violence and abuse.
Comparators	Controlled studies: usual care, no VAW intervention, delayed VAW intervention, minimal intervention (e.g., information provision). Uncontrolled studies: group before the intervention. No control group.	
Outcomes	Outcome is an event or measurement collected for participants in a study. Primary outcomes: any health outcomes for survivors of VAW (for example, re-exposure to VAW, sexual and reproductive health, mental health, physical healthy, quality of life), any harms, cost effectiveness of VAW interventions. AND/OR Secondary outcomes: patient and provider health-related cognitive and emotional outcomes (for example, knowledge, attitudes, confidence, readiness); health-related behaviour and practices (for example, identification and disclosure of VAW, provision and uptake of referrals and SRH services). Phenomenon of interest: provider and recipient experiences of and views on VAW interventions.	
Study type	Primary intervention studies of any design. Primary studies that used quantitative designs such as randomised controlled trials, controlled and uncontrolled before-after studies, interrupted time series studies, cross-sectional studies. Primary studies that used qualitative designs such as ethnographic research, interview or focus-group based studies, case studies, process evaluations and mixed methods designs. We include these studies if they had used qualitative methods for data collection and analysis and reported quotes from participants. Mixed-methods studies.	Systematic reviews. We used systematic reviews to identify potentially eligible primary studies.
Context	Studies conducted in SRH services in a country defined as LMIC by the World Bank, including humanitarian settings. Depending on country context, SRH services can be delivered at any level of healthcare provision and usually include contraceptive services, maternal and perinatal health, treatment for STI, HIV and reproductive tract infections, abortion, fertility treatment and gynaecological treatment.	
Report type	Full-text peer reviewed studies, conference abstracts, grey literature, unpublished studies.	Animal studies, opinion pieces, editorials, and publication which did not report primary data.

VAW violence against women, LMICs low-income and middle-income countries, SRH sexual and reproductive health, IPV intimate partner violence.

An information specialist (AR) applied the search strategy to Medline, Embase, Psycinfo, Cochrane, Cinahl, IMEMR, Web of Science, Popline, Lilacs, WHO RHL, ClinicalTrials.gov (20 August 2018 and 3-4 December 2019) (online supplementary file 1). AR searched for grey literature via Google, Google Scholar, and websites of key organisations in the field of VAW and SRH in LMICs (UNFPA, SVRI, JPHIEGO, USAID, WHO (IRIS) SEARO, WHO (IRIS) EMRO, World Bank). AR uploaded all records into EndNote and deduplicated. Two pairs of reviewers (NVL and MM, AFO and MC)

For peer review only

independently assessed eligibility. Disagreements were resolved through consensus or third opinion (LJB). NVL checked references and citations.

Data analysis

NVL adapted the Cochrane Effective Practice and Organisation of Care (EPOC) data extraction form.[22] We collated multiple reports from the same study and used the most detailed report as the primary source for extracting study results. The included studies were divided among reviewers who worked in pairs, one to extract data and another to check. The pairs reconciled data extraction through discussion. We extracted study details on setting, study design and aim, sample size, participants characteristics, intervention characteristics and theories, and outcomes relevant to our review questions. For each quantitative outcome, we extracted type of measure and effect estimates as reported in the primary study. If authors did not report intervention effects, we extracted the post-intervention point estimate. If a follow-up measure was reported repeatedly, we extracted the latest measure. We judged intervention effectiveness by improvement in any primary or secondary outcome listed in the individual studies (Table 1). We used authors' interpretation of their findings based on statistical significance or 95% confidence intervals (CI) and categorised effect estimates as *improvement, mixed effect,* or *null effect*. We ascribed a *mixed effect* when one or more, but not all measures of the same outcome changed under the same intervention (e.g., reduction in physical and sexual but not psychological IPV, improvement in some coping behaviours but not in others). NVL asked corresponding authors to check data extraction forms for their studies and provide missing information; nine responded.

Reviewers assessed the quality of the primary studies as part of data extraction. For randomised controlled trials (RCTs), we used the Revised Cochrane risk-of-bias tool for randomized trials (RoB 2).[23] For quasi-experimental studies we adapted the criteria listed by the EPOC Group.[24] For qualitative studies we adapted the Critical Appraisal Skills Programme (CASP) Qualitative Checklist.[25] We did not exclude studies based on their methodological quality.

We summarised interventions by mapping them onto the Health Systems Wheel[8] and models of health system responses to VAW in LMICs.[14] It was not possible to conduct a meta-analysis of quantitative outcomes due to the heterogeneity of the interventions, the outcomes, and their measurement. We undertook a narrative quantitative synthesis[26] and thematic qualitative synthesis,[27] summarised quantitative and qualitative syntheses in tables, and integrated them through a line of argument[18] and mapping onto a process-oriented logic model.[19] Reviewers (NVL, MC, LJB) drafted the logic model by mapping primary and secondary outcomes in the hypothesised logical order of occurrence and refined it through three iterative cycles of revisions.

For peer review only

1. Direct effects result from intervention activities producing structural changes at service level and changes in health-related cognitive and emotional outcomes among HCPs and women indicating improvement in the health systems capacity and women readiness to respond to VAW.
2. Intermediate effects result from direct effects producing changes in health-related behaviour and practices indicating improvement in the health systems capacity and women readiness to respond to VAW. HCPs identify women affected by VAW and provide first-line support; women disclose VAW, use offered support, develop adaptive coping strategies.
3. Health outcomes result from intermediate effects producing changes in women health and safety indicating improvement in their readiness to cope with VAW. Women use adaptive coping strategies and safety behaviours; these lead to reduction in re-exposure to VAW and better health.

PATIENT AND PUBLIC INVOLVEMENT

No patients or members of the public were involved in this study.

RESULTS

Searches identified 6082 records, we assessed 313 full text reports and included 32[28-59] reporting on 26 studies[28-35 38-40 42 45-47 49 50 52-60] (Figure 1, online supplementary file 2).

Characteristics of included studies

Of the 26 studies, 18 were from sub-Saharan Africa,[28 31-34 38 42 45-47 49 52-54 56 57 59 60] three from the Middle East,[40 55 58] three from South Asia,[29 39 50] and two from South America.[30 35] Twelve quantitative evaluations were randomised controlled trials (RCTs),[32 34 35 38 40 47 50 52 55 58-60] six were uncontrolled before-after (UBA) studies,[29 30 39 42 46 54] six cross sectional studies,[31 45 49 53 56 57] and one was a controlled before-after evaluation (CBA).[28] Nine qualitative evaluations were components of mixed-methods studies: three embedded in RCTs,[38 44 51] two carried out alongside UBA studies,[30 54] three alongside cross-sectional studies,[49 56 57] and one standalone qualitative study.[33] No two studies of similar design evaluated the same intervention and outcomes. The duration of follow up period ranged from two weeks[33] to 4 years and 7 months.[59]

Most interventions took place in antenatal care (ANC) services (n=11),[29 34 35 39 40 45 47 50 55 56 58] followed by HIV testing and treatment (n=8),[32 33 38 46 52 57 59 60] services for victims of sexual violence (n=5),[28 31 42 53 54] and family planning (n=2).[30 49] The SRH services were provided in primary care (n=15),[30 32-34 39 40 46 47 49 52 53 55 56 58 59] hospital (n=7),[29 35 38 42 45 50 57] and across both (n=4).[28 31 54 60] Included studies used different definitions and measures of VAW. A majority (n=11) targeted IPV.[32 33 35 38 39 45 46 49 55 57 59] Six interventions targeted sexual violence by intimate partners and non-partners.[28 31 42 52-54] Five interventions focused on domestic violence (DV) from any family member[29 34 40 50 58] and four targeted VAW from intimate partners and non-partners.[30 47 56 60] In studies that reported sample size, 901 HCPs received VAW interventions with the average sample size of 100 ranging from 4[49] to 408.[39] A total of

For peer review only

12,078 women of reproductive age received VAW interventions, with the average sample size of 549 ranging from 32[52] to 2,081.[31]

Quality appraisal

Most quantitative studies were at high risk of bias (online supplementary file 3). Of 12 RCTs, seven had high risk of bias from deviations in intervention adherence,[32 38 40 47 58-60] seven had high risk from measurement of outcomes[35 38 40 47 52 59 60] and seven had concerns from selective reporting of outcomes.[35 38 47 52 55 58 59] Of 13 non-randomised studies (all at high risk of bias), only four adequately addressed missing outcome data.[28 29 49 54] Of ten qualitative evaluations, five scored 15 and above on the 20-point CASP checklist,[44 45 49 51 56] indicating relatively high quality of research design and conduct. The main weaknesses were insufficient justification of methods, reporting of recruitment and strategies for neutrality.

For peer review only

Types of interventions

All interventions were complex healthcare interventions,[61] however only two[30 60] included components across all domains on the Health Systems Wheel[8] (Table 2) and only four were theoretically informed.[32 34 50 59]

Table 2. Included interventions mapped on the Health Systems Wheel framework and models of service integration

Study ID	Study design	Leadership and governance	Multi-sectoral coordination	Workforce development	Health-care delivery	Infrastructure	Financing	Information	Level of VAW service integration
Abeid 2016[28]	CBA			•	•	•	•	•	Systems
Arora 2019[29]	UBA				•		•		Provider
Bott 2014[30]	UBA	•	•	•	•	•	•	•	Facility
Bress 2018[31]	Cross-sectional		•	•	•	•	•	•	Provider
Brown 2018[32]	RCT				•		•		Facility
Cockcroft 2019[34]	cRCT			•	•	•	•	•	Provider
Cripe 2010[35]	RCT			•	•		•		Provider
Cristofides 2010[33]	Qualitative			•	•		•		Facility
Haberland 2016[38]	RCT			•	•	•	•	•	Facility
Jayatilleke 2015[39]	UBA			•	•		•		Provider
Khalili 2020[40]	RCT				•		•		Provider
Kim 2007[42]	UBA	•		•	•	•	•	•	Facility
Laisser 2011[45]	Cross-sectional			•	•		•	•	Systems
Matseke 2013[46]	UBA			•	•		•		Systems
Mutisya 2018[47]	RCT				•		•		Provider
Samandari 2016[49]	Cross-sectional	•	•	•	•	•	•	•	Systems
Sapkota 2020[50]	RCT				•		•		Provider
Settergren 2018[60]	cRCT	•	•	•	•	•	•	•	Systems
Sikkema 2018[52]	RCT			•	•		•		Provider
Sithole 2018[53]	Cross-sectional		•	•	•	•	•	•	Facility
Smith 2013[54]	UBA			•			•		Facility
Taghizadeh 2018[55]	RCT				•		•		Provider
Turan 2013[56]	Cross-sectional		•	•	•	•	•	•	Systems

Undie 2016[57]	Cross-sectional			•	•		•	•	Facility
Vakily 2018[58]	RCT			•			•		
Wagman 2015[59]	cRCT		•	•	•		•		Facility

Note. VAW violence against women. RCT randomised controlled trial. cRCT cluster randomised controlled trial. CBA controlled before-after. UBA uncontrolled before-after. Provider-level integration when one trained health care provider delivers most of the VAW work. Facility-level integration when several trained HCPs deliver most VAW work within one heath care facility. Systems-level integration when trained HCP identifies patients affected by VAW, provides first-line support and clinical care, and then refers them to higher level facilities with VAW specialist or external VAW services.

Most VAW work was delivered by a single HCP (n=10) or by several HCPs within the same facility (n=9). Only six interventions were integrated at a systems-level with HCPs identifying VAW cases, providing clinical care and first-line support, and referring to external VAW services.[28 45 46 49 56 60] We clustered 26 interventions into three categories based on the target group(s) and location of the common activities (online supplementary file 2):

1. *Response to VAW during routine SRH consultation* (n=10). These interventions aimed to strengthen health system capacity to respond to VAW through integrating identification and first-line support into routine SRH consultations.[28 32 33 38 39 45 46 54 57 58] This comprised training for HCPs in VAW screening, basic psychosocial counselling, and linkage to VAW resources. Training aimed to improve HCP knowledge, attitudes, and practices on VAW. Identification and response by trained HCPs aimed to increase women's readiness to respond to VAW. Duration of the integrated SRH-VAW consultation ranged between 7[32] and 30 minutes.[38 46]
2. *Response to VAW during routine SRH consultation plus community engagement* (n=9). These interventions aimed to strengthen health system capacity to respond to VAW across SRH service and surrounding communities.[30 31 34 42 49 53 56 59 60] Service-based activities were similar to the first category. The community-based activities aimed to shift gender norms and improve access to integrated SRH-VAW services through raising awareness about post-rape care,[31 42 53] education on gender and VAW,[30 49 53 56 59 60] and couples' education about VAW.[34 60] Integrated SRH-VAW consultations supported by community engagement aimed to increase women's readiness to respond to VAW.
3. *Response to VAW in addition to routine SRH consultation* (n=7). These interventions aimed to strengthen women's readiness to respond to VAW.[29 35 40 47 50 52 55] Study personnel screened women attending routine SRH services and delivered the interventions to self-selected women with experience of VAW. This comprised more intensive support through specialist psychosocial counselling,[29 35 47 50] coping skills training[52 55] and psychoeducation.[40 52 55] The average number of sessions was three (range 1 to 7) with each session lasting from 30[29 35 47] to 90 minutes.[40 52 55] Interventions were delivered face-to-face individually,[29 35 40 47 50] in a group,[55] and mixed format.[52]

Interventions effects and outcomes

For peer review only

For peer review only - http://bmjopen.bmj.com/site/about/guidelines.xhtml

The logic model displays all outcomes of interest in the three intervention categories (Figure 2). The arrows illustrate the hypothesised flow of change from intervention activities through health-related direct and intermediate effects to health outcomes.

None of the primary studies reported outcomes at service level. Most studies that evaluated interventions that aimed to strengthen health system capacity to respond to VAW across SRH service and community measured direct and intermediate effects on HCP and women's knowledge, attitudes, and behaviour. In contrast, all studies that evaluated interventions that aimed to strengthen women's readiness to cope with VAW reported their health outcomes, but only a few looked at preceding changes in women's cognition, emotions, and behaviour (Figure 2, Table 3).

For peer review only

Table 3. Health-related effects and outcomes in randomised and non-randomised evaluations of interventions addressing VAW in SRH services

	Intervention category	Improvement		Mixed effect		Null effect		Studies, n
		RCT	Non-randomised	RCT	Non-randomised	RCT	Non-randomised	
**Response to VAW during routine SRH consultation (n=10)**								
**Direct effect on health-related cognition and emotions**	HCP knowledge	**Vakily 2017[58]**	Jayatilleke 2015[39]<br>Smith[54]<br>Abeid 2016[28]					4
	HCP attitudes		Smith 2013[54]				**Vakily 2017[58]**<br>Abeid 2016[28]	3
	HCP readiness		Jayatilleke 2015[39]<br>Smith 2013[54]					2
	Women's knowledge	Haberland 2016[38]						1
	Women's attitude					Haberland 2013[38]		1
	Women's readiness					Haberland 2013[38]		1
**Intermediate effects on health-related behaviour and practices**	HCP behaviour		Jayatilleke 2015[39]	Haberland 2013[38]	Smith 2013[54]<br>Abeid 2016[28]			4
	Women's behaviour			Haberland 2016[38]		Brown 2018[32]		2
**Health outcomes**	Re-exposure to VAW	Brown 2018[32]	Matseke[46]			Haberland 2016[38]		3
	Any harm					Brown 2018[32]<br>Haberland[38]		2
**Response to VAW during SRH consultation plus community engagement**								
**Direct effects on health-related cognition and emotions**	HCP attitudes		Bott 2004[30]					1
	HCP readiness				Bott 2004[30]			1
	Women attitude			Settergren 2018[60]				1
**Intermediate effect on behaviour and practices**	HCP behaviour					Settergren 2018[60]		1
	Women behaviour		Kim 2007[42]	Settergren 2018[60]		**Cockcroft 2019[34]**<br>Wagman 2015[59]		4
**Health outcomes**	Re-exposure to VAW	**Cockcroft 2019[34]**		Wagman 2015[59]		Settergren 2018[60]		3
	Sexual and reproductive health			**Cockcroft 2019[34]**		Wagman 2015[59]		2
**Response to VAW in addition to routine SRH consultation**								

For peer review only

	Intervention category	Improvement		Mixed effect		Null effect		Studies, n
		RCT	Non-randomised	RCT	Non-randomised	RCT	Non-randomised	
**Intermediate effects on health-related behaviour and practices**	Women behaviour	**Sapkota 2020[50]**		Sikkema 2018[52]		Cripe 2010[35]	Arora 2019[29]	4
**Health outcomes**	Re-exposure to VAW	**Khalili 2019[40]**<br>**Mutisya 2018[47]**	Arora 2019[29]	Tanghizaden 2018[55]				4
	Sexual and reproductive health					Sikkema 2018[52]		1
	Physical health		Arora 2019[29]					1
	Mental health	**Khalili 2019[40]**<br>**Mutisya 2018[47]**<br>**Sapkota 2020[50]**	Arora 2019[29]			Cripe 2010[35]<br>Sikkema 2018[52]		6
	Quality of life	**Sapkota 2020[50]**				Cripe 2010[35]		1
	Studies, n	7	7	6	2	7	3	

Note. HCP health-care providers, VAW violence against women, SRH sexual and reproductive health., RCT randomised controlled trial. Bold indicates studies that reported sample size calculation.

For peer review only

Direct effects on cognition and emotions. The routine SRH-VAW intervention category had overall positive direct effects on HCP and women's knowledge, attitudes, and readiness. Interventions with community engagement reported mixed and improved direct effects.

Intermediate effects on behaviour and practices. Changes in professional behaviour were measured through the rates of VAW screening, provision of referrals to support services, and post-rape care. Changes in women's behaviour were measured through VAW disclosure, uptake of referrals and other services. The overall evidence was uncertain across all three intervention categories with RCTs and non-randomised evaluations reporting improved, mixed, and null effects.

Health outcomes. Only half the studies reported measures of health and re-exposure to VAW; two interventions reported no harm resulting from taking part in interventions,[32 38] and 10 led to some health improvement. The overall direction of effect on any outcomes of interest was towards improvement in the *routine SRH-VAW consultation* category and in the *additional response* category. In contrast, most interventions in the *plus community engagement* category reported mixed or null effect on women's health and re-exposure to VAW. We found that although some interventions did not reduce re-exposure to VAW, none reported violence escalation. Of 10 studies that measured re-exposure to VAW, six found a reduction,[29 32 34 40 46 47] two reductions in some violence types but not in others,[55 59] and two reported no change.[38 60]

Of 26 studies, only two reported changes across all three domains of the logic model, one from the *routine SRH-VAW consultation* category[38] and one from the *plus community engagement* category.[60] Four evaluations of the *additional response* category reported changes across two domains - intermediate effects on behaviour and practices and women's health outcomes.[29 35 50 52] These six studies were consistent with our hypotheses. If intervention improved women's safety behaviour and use of support services, their health improved.[50] Mixed or null effect on HCP and women's cognitive and behavioural outcomes suggested some explanation for no change in re-exposure to VAW.[38 60] Contradicting direct and indirect effects and outcomes[29 52] indicated possible barriers on the pathway from intervention activities to outcomes.

Response to VAW during routine SRH consultation

Of ten evaluations, two RCTs[32 38] and one UBA[46] studies reported conflicting findings on re-exposure to VAW; none measured women's health (Figure 2, Table 3, Supplementary file 4). These three interventions did not lead to escalation of violence. There was some evidence for the reduction in HIV-disclosure-related violence at up to 2-month follow-up[32] and risk of becoming a victim of femicide at 3-month follow-up[46] possibly through some improvement in HCP's and women's cognition and practice. Two RCTs reported that integrated HIV-IPV consultation caused no harm to women.[32 38] However, all studies were at high risk of bias.

An RCT[38] and UBA[57] in the Kenyan hospital with on-site GBV centre reported convergent findings. The UBA study of an integrated HIV-IPV consultation with assisted referral to GBV centre reported 8% IPV disclosure rate, 75% referrals provision and 40% uptake.[57] The RCT of an integrated HIV-IPV consultation with referral to in-service GBV specialist found increased rates of IPV screening but no effect on provision of referrals. The 29-minute integrated HIV-IPV consultation increased women's knowledge about VAW and IPV disclosure, but had no effect on their attitudes, readiness to address VAW, uptake of referrals and re-exposure to IPV.[38]

Another RCT of a 7-minute integrated HIV-IPV consultation over the phone reported that 62% of women used a safety plan and 80% employed at least one safety strategy, however their use of SRH services and perceived risk and safety did not change. Despite no effect on women's behaviour, the trial reported a four-fold reduction in HIV-disclosure-related violence (OR 4.37; 95% CI 1.46-13.44).[35] One UBA found that a 30-minute integrated HIV-IPV consultation contributed towards a reduction in the risk of femicide (mean difference 3.2, SD 3.56; 95% CI 2.43-3.98).[32]

Other randomised and non-randomised evaluations of varied one-off training for HCPs reported conflicting findings on their knowledge, attitudes, and readiness to address VAW. Non-randomised studies reported increased IPV screening rates,[39] low VAW disclosure rates,[45] and mixed effect on provision of post-rape care.[28 54]

Qualitative evaluations confirmed that training increased HCP ability to respond to VAW during routine SRH consultations.[38 45 54 57] One evaluation of HCP training on post-rape care described a potential mechanism of impact on HCP negative attitudes by separating personal beliefs about victims from the provision of clinical care.[54] Women found that integrated HIV-IPV consultation improved their knowledge about IPV. They benefited from emotional support and felt empowered.[33 38 57]

Response to VAW during routine SRH consultation plus community engagement

Three cluster RCTs reported conflicting findings on women's SRH and re-exposure to VAW. The overall effect was uncertain (Figure 2, Table 3, Supplementary file 5).[34 59 60] A Nigerian RCT at low risk of bias evaluated universal home visits that discussed DV and other risk factors with pregnant women and their spouses. The trial reported no effect on women's use of SRH services, reduction in the proportion who experienced physical DV (RD 0.064 (95% CI 0.045-0.084), and mixed effect on pregnancy and birth indicators.[34] A Tanzanian RCT at high risk of bias evaluated integrated HIV-VAW consultation, onsite and external referrals, community, and couple education. Intervention had a mixed effect on women's attitudes about VAW and gender roles, no effect on rates of enquiry and referrals, mixed effect on women's use of SRH services, and null effect on re-exposure to IPV (OR = 0.85, 95% CI 0.62-1.16).[60] A Ugandan RCT at high risk of bias evaluated integrated HIV-IPV consultation, onsite referral, and community education. The intervention had no effect on women's safety behaviour and null effect on SRH. Re-exposure to physical and sexual IPV reduced (relative

For peer review only

prevalence risk ratios (PRR) of 0.74 (95% CI 0.63-0.86), 0.75 (95% CI 0.62-0.90), respectively), but psychological IPV and HIV did not change.[59]

Non-randomised studies reported more positive effects on HCP knowledge, attitudes, preparedness,[30] and provision and use of post-rape care.[31 42 53] They also reported high rates of IPV screening and provision of referrals and clinical care by HCPs vs low uptake of referrals and other services by women.[49 56]

Qualitative evaluations confirmed that VAW training transformed HCP attitudes towards patients and their own work and improved their diagnostic and counselling skills. HCPs appreciated the intervention and expressed a willingness to continue VAW work.[30 49 56 57] Women felt empowered and supported by HCPs.[30 33 45 49] Community engagement raised awareness about SRH-VAW services.[56]

Response to VAW in addition to routine SRH consultation

This intervention category had the most robust evidence from six RCTs[35 40 47 50 52 55] and one UBA study[29] (Figure 2, Table 3, Supplementary file 6). The studies reported conflicting results with more evidence for a reduction in re-exposure to VAW at up to 6-month post-intervention and improvement in health possibly through improvement in women's safety behaviour. There was some evidence that longer interventions produced better outcomes.[29 40 47]

Psychosocial counselling. Three RCTs of counselling sessions for pregnant women with experience of VAW reported conflicting results with no evidence for a dose-response.[35 47 50] The overall effect was towards reduction in re-exposure to violence and improvement in health outcomes. An adequately powered Nepalese RCT with low risk of bias evaluated a 35-45-minute psychosocial counselling session with a resource card and counsellor's contact details. The trial reported positive effects on women's self-efficacy (MD 0.5; 95% CI 0.30-0.72), perceived social support (MD 0.73; 95% CI 0.39-1.06), safety behaviours (MD 2.41; 95% CI 1.43-3.40), anxiety (MD -3.73; 95% CI -5.42 to -2.04), depression (MD -3.41; 95% CI -4.84 to -1.99), and quality of life (MD 2.45; 95% CI 1.51-3.39).[50] The embedded qualitative study confirmed that women felt empowered, supported, and valued by the counsellor.[51] In contrast, a Peruvian RCT (with some bias concerns) of a 30-minute counselling session with a resource card and external referral had no effect on women's safety behaviours, health, use of community resources.[35] A Kenyan RCT of up to three 30-35-minute counselling sessions with resource card, safety planning and external referral reduced depression (MD = 7.12; 95% CI 6.21-8.03) and re-exposure to IPV (MD = 13.51; 95% CI 9.99-17.02).[47] Similarly, an Indian UBA evaluation of two or more 30-45-minute psychosocial counselling sessions found that most women had increased awareness of and readiness to address VAW. Physical violence and health problems decreased.[29]

For peer review only

Coping skills training. Two RCTs with high risk of bias evaluated more intensive training interventions and found mixed effects on behaviour and mixed and null effects on VAW and health.[52 55] An Iranian RCT of four 90-minute group sessions reported a reduction in re-exposure to physical IPV (RR 0.78; 95% CI 0.63-0.83) and psychological IPV (RR 0.73; 95% CI 0.64-0.83), but null effect on sexual IPV (RR 0.87; 95% CI 0.69-1.09).[55] A South African RCT of seven 90-minute sessions reported null effect on coping behaviour, use of SRH services, post-traumatic stress disorder (PTSD) and HIV viral load among HIV positive women with a history of sexual violence.[52] However, the embedded qualitative evaluation found that training increased women's knowledge about VAW impact and improved their self-esteem, coping and communication skills.[44]

Psychoeducation. An Iranian RCT with high risk of bias of four 90-minute sessions with pregnant women reported reduction in scores of IPV and psychological distress.[40 41]

Cost effectiveness outcomes

No studies evaluated cost effectiveness of VAW interventions in SRH services. One study of an integrated HIV-IPV consultation paid HCPs $6 per day for identifying patients experiencing VAW and referring them to the on-site GBV clinic.[57] One evaluation of post-rape service improvement with community engagement reported resource costs.[42 43] Seven studies across all three intervention categories mentioned intervention costs but did not report actual data.[30 38 47 50 53 56 57]

Barriers to intervention effects and outcomes

Supplementary file 7 summarises factors that women and HCPs perceived as barriers to intervention implementation and impact. We developed three analytical themes cross-cutting through individual, community, and system levels.

Acceptability of VAW. Four evaluations of interventions on *response during routine SRH consultation* and *response with community engagement* described attitudes accepting violence and patriarchal gender norms as major barrier to behaviour change.[33 45 54 56]

Fear of negative consequences. Eight studies across all three interventions categories identified fear of negative consequences as a barrier to identification, disclosure, and engagement in VAW interventions.[30 33 38 44 45 49 51 53]

Limited readiness. Evaluations reported limited readiness for engaging in VAW interventions at system and individual levels. In evaluations of *response to VAW during routine SRH consultation*[28 33 38 45 57] and *response with community engagement*,[30 49] HCPs consistently mentioned chronic problems with staffing, inadequate funding, no private space, lack of support from leadership, and high demand for basic SRH services without the additional VAW work. Readiness gaps at system level included the lack of services to refer to, poor referral systems and untrained staff in other agencies. Screening identified many IPV-positive women and specialist services could not address the increased

For peer review only

demand.[30 33 45 56] Across all intervention categories, HCPs and women described barriers at societal level that prevented women from accessing SRH services, using referrals, and participating in psychosocial interventions. Work-related conflicts, no money for transport, and financial dependence on husband were mentioned most frequently.[38 44 45 53] Finally, two evaluations of *response to VAW during routine SRH consultation* explored reasons for low uptake of referrals to specialist services. Some women had expectations that could not be met by the current services. Instead of referral, they wanted HCPs to talk to their partners about stopping the abuse.[33] Some women wanted to receive all SRH and VAW services on the same day.[38 57]

DISCUSSION

We conducted a mixed-methods systematic review of studies from LMICs on the effectiveness and barriers to strengthening SRH services response to VAW. We grouped 26 heterogeneous complex interventions into three categories: (i) response to VAW during routine SRH consultation, (ii) response to VAW during routine SRH consultation plus community engagement, and (iii) response to VAW in addition to routine SRH consultation. We mapped outcomes on a process-oriented logic model illustrating the hypothesised changes from intervention through direct and intermediate effects on health-related cognition, emotions, and behaviour to health outcomes. We cannot conclude which intervention was the most effective in improving any of these effects and outcomes due to heterogeneity of the interventions and measures at varying time points. Overall, ten interventions did not escalate violence and two reported no harmful events. We found mixed effects on women's health and re-occurrence of VAW across all three categories, with studies reporting conflicting findings. Evaluations of the varied *responses to VAW during routine SRH consultation* found reduction in HIV-disclosure-related IPV and potential risk of becoming a victim of femicide, but no effect on IPV in the past month. Some of these effects could be attributed to improvement in HCPs' readiness, screening, and provision of first-line support for VAW. For women, these effects could be attributed to increased knowledge about VAW and disclosure of violence. *Response to VAW during routine SRH consultation plus community engagement* had uncertain evidence with single studies reporting improvement, mixed effect, and no effect on re-exposure to violence and SRH possibly through some improvement in provision and use of SRH services. More intensive psychosocial interventions delivered to women with experience of VAW *in addition to routine SRH consultation* had the most robust evidence for reduction in re-occurrence of violence and improvement in health outcomes possibly through an improvement in safety behaviours. We identified individual, community, and system-level barriers that could disrupt the pathway from intervention activities to outcomes: (i) attitudes and social norms that accept and normalise violence, (ii) fear of negative consequences and (iii) limited readiness of individuals, health systems and society to address VAW. No studies reported cost-effectiveness analysis.

Strengths and limitations

For peer review only

This review is methodologically strong. It involved a multidisciplinary team of researchers from LMICs and the UK with content and methodological expertise in health systems response to VAW and global health. We followed the Cochrane method and included studies of any design reported in peer-reviewed and grey literature in any language with English abstract. This comprehensive approach ensured inclusion of the most relevant studies from the field and reduced the potential for bias/errors.

The evidence for VAW interventions in SRH settings is weak because of the methodological limitations of the primary studies and uncertain effectiveness of the interventions. Each study used differing operational definitions and outcomes measures, relied on self-report, and evaluated a different complex intervention. No studies measured contextual and implementation factors nor adjusted their analysis for those factors which could mediate the effect of the intervention on outcomes of individual HCPs and women, although those were explored in some qualitative evaluations. Because of the diverse complex interventions and outcomes measures we could not perform a meta-analysis. Our findings should be interpreted with caution because two thirds of trials and all 13 quasi-experimental studies had high risk of bias.

The evidence we found is applicable to ANC and HIV services and depends on the intervention category. Interventions that strengthened capacity of HIV and ANC services to respond to VAW can increase identification and provision of first-line support to women experiencing violence which can lead to reduction in HIV-disclosure-related IPV, physical and sexual IPV, and the risk of becoming a victim of femicide. More intensive psychosocial interventions that strengthen women's readiness to cope with VAW can increase use of SRH services and safety behaviours, reduce re-exposure to IPV and DV, and improve health and quality of life. The positive effect of additional psychosocial interventions can be explained by their theoretical underpinning, higher dose of provider-patient contact, delivery by study personnel specialised in counselling and VAW, and samples of self-selected women who could be more motivated and ready for change. The first two intervention categories might appear less effective because few studies examined women's outcomes beyond the point of identification and first-line response. Our findings suggest that future VAW interventions should strengthen multi-level capacity across individual HCPs and women, SRH services, and communities.

The uncertain evidence for the two SRH consultation-based intervention categories is consistent with other evidence for a healthcare response to VAW in LMICs[15 62] and to VAW among pregnant women.[16 63 64] The evidence for the effectiveness of longer psychosocial interventions as an addition to routine SRH consultations is in line with a recent meta-analysis which found that psychosocial interventions in healthcare settings and communities in LMICs led to a 25-27% reduction in IPV.[65]

For peer review only

An important finding on the direct effects of interventions is that increasing awareness about VAW and relevant procedures often did not lead to a shift in judgemental attitudes towards victims, the major barrier to changes in professional and patient behaviour and practices. An exception was studies in the *plus community engagement category*, one reporting improvement in HCP attitudes and one partial shift in women's attitudes and gender norms. These findings can be explained by the community and societal roots of gender norms and attitudes of HCPs and women which are best addressed at community and societal levels. Our findings suggest that a shift in individual's attitudes potentially leading to behavioural change can be achieved through service-based plus community-based education. This finding is consistent with a review of evidence on what works to prevent VAW in LMICs. The review found good evidence for community activism approaches to shift harmful gender attitudes, roles and social norms.[66]

An important finding is that routine integrated SRH-VAW consultations with referral or signposting to VAW/GBV specialist or other services did not increase women's use of these services. This gap between availability and acceptability of referrals to other services has several explanations. Our themes of barriers to intervention impact suggested that HCP response may not have matched women's needs and expectations, or the VAW services were not accessible, or contextual factors prevented women from accessing them. This finding suggests that integrated SRH-VAW consultation and VAW services require better tailoring to women's needs and expectations. This should be based on understanding what women want and need and what is feasible. A recent qualitative meta-synthesis found that after disclosing IPV to HCPs, women wanted assistance with documentation of injuries, insurance issues and help with connecting to community services more than referrals to IPV services.[67] The feasibility and acceptability of HCPs engaging with men who use violence needs further exploration in LMICs.

Finally, most interventions in the first two categories targeted the behaviour of individual HCPs rather than the SRH service or health system. The expectation was that trained HCPs would integrate VAW work into their clinical practice without structural changes to the environment, support from leadership, supervision, monitoring and incentivisation. Most qualitative evaluations described passionate HCPs who were enthusiastic about helping patients experiencing violence. However, some studies reported HCPs concerns about unrealistic expectations and limited health system readiness for embedding VAW work in routine practice. This finding is in line with other studies on health systems readiness for responding to VAW.[9 10] The obstacles to integrating a VAW response in SRH services overlap with those reported in the systematic review of barriers and facilitators to integrating health systems responses to IPV in LMICs.[68]

This review's results are relevant for practitioners and policy makers in LMICs. The logic model approach allowed us to: (i) illustrate the hypothesised cause-result pathway, (ii) map evidence from

For peer review only

primary studies for the direct and intermediate effects and outcomes, (iii) identify barriers that can disrupt the trajectory of changes. It allowed us to present evidence in a format understandable to end users: people who develop, deliver, evaluate and fund VAW interventions in LMICs. We need more methodologically robust evaluations of interventions for strengthening the capacity of the health systems, communities, and individual women to respond to VAW with measures throughout the pathway from intervention activities to women's outcomes. Absence of evidence on the cost-effectiveness of VAW interventions in SRH services is another gap. Finally, very few interventions have been evaluated in LMICs outside Africa.

CONCLUSION

We found that interventions to improve response to VAW in SRH services did not escalate violence. Some interventions that strengthened capacity of HIV and ANC services increased identification and reduced some types of IPV. Some interventions that strengthened capacity of HIV and ANC services and communities improved use of SRH services and reduced re-exposure to some types of VAW. Several studies identified a gap between provision and uptake of referrals to VAW services suggesting that first-line support should be better tailored to women's needs and preferences. Most additional psychosocial interventions that strengthened women's readiness to respond to VAW reduced re-exposure to violence and improved health. Our findings are relevant to people who develop, implement, evaluate, and fund VAW interventions in healthcare. Future interventions should have better theoretical development and use a systemic approach to strengthen the capacity to respond to VAW across the healthcare systems, communities, and women. Future evaluations of VAW interventions in SRH services in LMICs should have longer follow-up and use standardised measures of individual-, organisation-, and system- level outcomes on the pathway from intervention to women's health.

CONTRIBUTORS

GF, LJB, NVL planned the review. All co-authors contributed to the protocol development. AR constructed and ran searchers. NVL and MM screened titles and abstracts. NVL, MM, LJB, MC, AFO screened full texts. NVL, MM, MC, AFO, SP, SS, TR, AS, PR, AA, AR, LJB worked in pairs on data extraction, risk assessment. NVL summarised the findings. NVL, MC, LJB developed logic model. NVL wrote first draft of the manuscript. All co-authors contributed to a further two revisions and approved final manuscript.

FUNDING

This research was funded by the National Institute for Health Research (NIHR) (17/63/125) using UK aid from the UK Government to support global health research. The views expressed in this publication are those of the authors and not necessarily those of the NIHR or the UK government.

For peer review only

AR was funded by the NIHR Applied Research Collaboration West (NIHR ARC West). The views expressed in this article are those of the authors and not necessarily those of the NIHR or the Department of Health and Social Care.

DISCLAIMER

The funder of the study had no role in study design, data collection, data analysis, data interpretation, or writing of the report. The corresponding author had full access to all the data in the study and had final responsibility for the decision to submit for publication.

COMPETING INTERESTS

None declared.

ETHICS APPROVAL

Ethics approval was not required. All data used in this review were already in the public domain.

PATIENT CONSENT FOR PUBLICATION

Not required.

DATA SHARING STATEMENT

No additional data available. All data relevant to the study are included in the article and uploaded as supplementary files.

REFERENCES

1. UN General Assembly. Declaration on the Elimination of Violence against Women. https://www.refworld.org/docid/3b00f25d2c.html UN General Assembly, 20 December 1993.

2. Campbell JC. Health consequences of intimate partner violence. *Lancet* 2002;359(9314):1331-6. doi: 10.1016/S0140-6736(02)08336-8 [published Online First: 2002/04/20]

3. WHO. Violence against women prevalence estimates, 2018. Global, regional and national prevalence estimates for intimate partner violence against women and global and regional prevalence estimates for non-partner sexual violence against women. Geneva: World Health Organization, on behalf of the United Nations Inter-Agency Working Group on Violence Against Women Estimation and Data (UNICEF, UNFPA, UNODC, UNSD, UNWomen), 2021:112.

4. Miltz AR, Lampe FC, Bacchus LJ, et al. Intimate partner violence, depression, and sexual behaviour among gay, bisexual and other men who have sex with men in the PROUD trial. *BMC Public Health* 2019;19(1):431. doi: 10.1186/s12889-019-6757-6 [published Online First: 2019/04/27]

5. Tarzia L, Forsdike K, Feder G, et al. Interventions in Health Settings for Male Perpetrators or Victims of Intimate Partner Violence. *Trauma Violence Abuse* 2020;21(1):123-37. doi: 10.1177/1524838017744772 [published Online First: 2018/01/16]

For peer review only

6. Ansara DL, Hindin MJ. Formal and informal help-seeking associated with women's and men's experiences of intimate partner violence in Canada. *Soc Sci Med* 2010;70(7):1011-8. doi: 10.1016/j.socscimed.2009.12.009 [published Online First: 2010/02/04]

7. Bonomi AE, Anderson ML, Rivara FP, et al. Health care utilization and costs associated with physical and nonphysical-only intimate partner violence. *Health Serv Res* 2009;44(3):1052-67. doi: 10.1111/j.1475-6773.2009.00955.x [published Online First: 2009/08/14]

8. Garcia-Moreno C, Hegarty K, d'Oliveira AF, et al. The health-systems response to violence against women. *Lancet* 2015;385(9977):1567-79. doi: https://dx.doi.org/10.1016/S0140-6736(14)61837-7

9. Colombini M, Alkaiyat A, Shaheen A, et al. Exploring health system readiness for adopting interventions to address intimate partner violence: a case study from the occupied Palestinian Territory. *Health Policy Plan* 2020;35(3):245-56. doi: 10.1093/heapol/czz151 [published Online First: 2019/12/13]

10. d'Oliveira A, Pereira S, Bacchus LJ, et al. Are We Asking Too Much of the Health Sector? Exploring the Readiness of Brazilian Primary Healthcare to Respond to Domestic Violence Against Women. *Int J Health Policy Manag* 2020 doi: 10.34172/ijhpm.2020.237 [published Online First: 2020/12/18]

11. Colombini M, Mayhew SH, Ali SH, et al. An integrated health sector response to violence against women in Malaysia: lessons for supporting scale up. *BMC Public Health* 2012;12:548. doi: 10.1186/1471-2458-12-548 [published Online First: 2012/07/26]

12. WHO. Health care for women subjected to intimate partner violence or sexual violence. A clinical handbook. Luxembourg: WHO, 2014:108.

13. WHO. Strengthening health systems to respond to women subjected to intimate partner violence or sexual violence: a manual for health managers. Geneva, Switzerland.: World Health Organisation, 2017.

14. Colombini M, Mayhew S, Watts C. Health-sector responses to intimate partner violence in low- and middle-income settings: a review of current models, challenges and opportunities. *Bulletin of the World Health Organization* 2008;86(8):635-42.

15. Kirk L, Terry S, Lokuge K, et al. Effectiveness of secondary and tertiary prevention for violence against women in low and low-middle income countries: a systematic review. *BMC Public Health* 2017;17(1):622. doi: https://dx.doi.org/10.1186/s12889-017-4502-6

For peer review only

16. Sapkota D, Baird K, Saito A, et al. Interventions for reducing and/or controlling domestic violence among pregnant women in low- and middle-income countries: a systematic review. *Syst Rev* 2019;8(1):79. doi: 10.1186/s13643-019-0998-4 [published Online First: 2019/04/04]

17. WHO. Responding to intimate partner violence and sexual violence against women: WHO clinical and policy guidelines. https://www.who.int/reproductivehealth/publications/violence/9789241548595/en/: WHO, 2013:68.

18. Sandelowski M, Voils CI, Barroso J. Defining and Designing Mixed Research Synthesis Studies. *Res Sch* 2006;13(1):29. [published Online First: 2006/04/01]

19. Rohwer A, Pfadenhauer L, Burns J, et al. Series: Clinical Epidemiology in South Africa. Paper 3: Logic models help make sense of complexity in systematic reviews and health technology assessments. *J Clin Epidemiol* 2017;83:37-47. doi: 10.1016/j.jclinepi.2016.06.012 [published Online First: 2016/10/30]

20. Higgins J, Thomas J, Chandler J, et al. Cochrane handbook for systematic reviews of interventions version 6.1 (updated September 2020). Second edition. ed. www.training.cochrane.org/handbook: Cochrane, 2020:pages cm.

21. Page MJ, McKenzie JE, Bossuyt PM, et al. The PRISMA 2020 statement: an updated guideline for reporting systematic reviews. *BMJ* 2021;372:n71. doi: 10.1136/bmj.n71 [published Online First: 2021/03/31]

22. Cochrane Effective Practice and Organisation of Care (EPOC). Data collection form. EPOC Resources for review authors. epoc.cochrane.org/resources/epoc-specific-resources-review-authors 2017.

23. Higgins J, Savović J, Page M, et al. Chapter 8: Assessing risk of bias in a randomized trial. In: Higgins J, Thomas J, Chandler J, et al., eds. Cochrane Handbook for Systematic Reviews of Interventions version 61 (updated September 2020). https://training.cochrane.org/cochrane-handbook-systematic-reviews-interventions: Cochrane 2020.

24. EPOC. Suggested risk of bias criteria for EPOC reviews. Cochrane Effective Practice and Organisation of Care (EPOC) EPOC Resources for review authors. epoc.cochrane.org/resources/epoc-resources-review-authors, 2017.

25. Critical Appraisal Skills Programme. CASP Qualitative Checklist [online]. https://casp-uk.net/casp-tools-checklists/, 2018.

For peer review only

26. EPOC. Synthesising results when it does not make sense to do a meta-analysis. EPOC Resources for review authors. https://epoc.cochrane.org/sites/epoc.cochrane.org/files/public/uploads/Resources-for-authors2017/synthesising_results_when_meta-analysis_does_not_make_sense.pdf, 2017.

27. Thomas J, Harden A. Methods for the thematic synthesis of qualitative research in systematic reviews. *BMC Med Res Methodol* 2008;8:45. doi: 10.1186/1471-2288-8-45 [published Online First: 2008/07/12]

28. Abeid M, Muganyizi P, Mpembeni R, et al. Evaluation of a training program for health care workers to improve the quality of care for rape survivors: a quasi-experimental design study in Morogoro, Tanzania. *Glob Health Action* 2016;9:31735. doi: https://dx.doi.org/10.3402/gha.v9.31735

29. Arora S, Deosthali PB, Rege S. Effectiveness of a counselling intervention implemented in antenatal setting for pregnant women facing domestic violence: a pre-experimental study. *BJOG* 2019;126 Suppl 4:50-57. doi: 10.1111/1471-0528.15846 [published Online First: 2019/07/02]

30. Bott S, Guedes A, Guezmes A, et al. Improving the health sector response to gender-based violence: A resource manual for health care professionals in developing countries. https://www.paho.org/hq/index.php?option=com_content&view=article&id=4517:2010-improving-health-sector-response-gender-violence-manual&Itemid=41342&lang=en: International Planned Parenthood Federation, Western Hemisphere Region., 2004:248.

31. Bress J, Kashemwa G, Amisi C, et al. Delivering integrated care after sexual violence in the Democratic Republic of the Congo. *BMJ Glob Health* 2019;4(1):e001120. doi: 10.1136/bmjgh-2018-001120 [published Online First: 2019/03/23]

32. Brown LL, Van Zyl MAR. Mitigating intimate partner violence among South African women testing HIV positive during mobile counseling and testing. *Aids Care-Psychol Socio-Med Asp Aids-Hiv* 2018;30(1):65-71. doi: 10.1080/09540121.2017.1366414

33. Christofides N, Jewkes R. Acceptability of universal screening for intimate partner violence in voluntary HIV testing and counseling services in South Africa and service implications. *AIDS Care* 2010;22(3):279-85. doi: https://dx.doi.org/10.1080/09540120903193617

34. Cockcroft A, Omer K, Gidado Y, et al. The impact of universal home visits with pregnant women and their spouses on maternal outcomes: a cluster randomised controlled trial in Bauchi State, Nigeria. *BMJ Glob Health* 2019;4(1):e001172. doi: 10.1136/bmjgh-2018-001172 [published Online First: 2019/03/23]

35. Cripe SM, Sanchez SE, Sanchez E, et al. Intimate partner violence during pregnancy: a pilot intervention program in Lima, Peru. *Journal of Interpersonal Violence* 2010;25(11):2054-76. doi: https://dx.doi.org/10.1177/0886260509354517

For peer review only

36. Delamou A, Samandari G. Integrating intimate partner violence screening and counseling with family planning services: Experience in Conakry, Guinea. The RESPOND Project Study Series: Contributions to Global Knowledge—Report No. 16. The RESPOND Project Study Series: Contributions to Global Knowledge. http://www.respond-project.org/pages/pubs/research-reports.php, 2014:78.

37. Guedes A, Bott S, Cuca Y. Integrating systematic screening for gender-based violence into sexual and reproductive health services: results of a baseline study by the International Planned Parenthood Federation, Western Hemisphere Region. *Int J Gynaecol Obstet* 2002;78 Suppl 1:S57-63. doi: 10.1016/S0020-7292(02)00045-0 [published Online First: 2002/11/14]

38. Haberland N, Ndwiga C, McCarthy K, et al. Addressing intimate partner violence and power in relationships in HIV testing services: Results of an intervention piloted in Nairobi, Kenya. HIVCore final report: Washington, D.C., USAID | Project Search: HIVCore, 2016:50 p.

39. Jayatilleke AC, Yoshikawa K, Yasuoka J, et al. Training Sri Lankan public health midwives on intimate partner violence: a pre- and post-intervention study. *BMC Public Health* 2015;15:331. doi: https://dx.doi.org/10.1186/s12889-015-1674-9

40. Khalili Z, Navaee M, Shakiba M, et al. [The effect of supportive-educational intervention on psychological distress in pregnant women subjected to domestic violence: A randomized controlled trial]. *Hayat, Journal of School of Nursing and Midwifery, Tehran University of Medical Sciences* 2019;25(2):151-67 (Persian).

41. Khalili Z, Navaee M, Shakiba M, et al. The Effect of a Supportive-Educational Intervention on Maternal-Fetal Attachment of Pregnant Women Facing Domestic Violence: A Randomized Controlled Trial. *Iranian Journal of Psychiatry and Behavioral Sciences* 2020;14(1) doi: ARTN e92070

10.5812/ijpbs.92070

42. Kim J, Mokwena L, Ntlemo E, et al. Developing an integrated model for post-rape care and HIV post-exposure prophylaxis in rural South Africa: [Johannesburg], South Africa, Population Council, Frontiers in Reproductive Health, 2007 Nov., 2007:[52] p.

43. Kim JC, Askew I, Muvhango L, et al. Comprehensive care and HIV prophylaxis after sexual assault in rural South Africa: the Refentse intervention study. *BMJ* 2009;338:b515. doi: https://dx.doi.org/10.1136/bmj.b515

44. Knettel BA, Mulawa MI, Knippler ET, et al. Women's perspectives on ImpACT: a coping intervention to address sexual trauma and improve HIV care engagement in Cape Town, South

For peer review only

Africa. *AIDS Care* 2019;31(11):1389-96. doi: 10.1080/09540121.2019.1587368 [published Online First: 2019/03/02]

45. Laisser RM, Nystrom L, Lindmark G, et al. Screening of women for intimate partner violence: a pilot intervention at an outpatient department in Tanzania. *Glob Health Action* 2011;4:7288. doi: 10.3402/gha.v4i0.7288 [published Online First: 2011/10/27]

46. Matseke G, Peltzer K, Habil. Screening and brief intervention for intimate partner violence among antenatal care attendees at primary healthcare clinics in Mpumalanga province, South Africa. *South African Journal of Obstetrics and Gynaecology* 2013;19(2):40-43. doi: http://dx.doi.org/10.7196/SAJOG.637

47. Mutisya RK, Ngure K, Mwachari C. A psychosocial intervention to reduce gender-based violence and antepartum depressive symptoms in pregnant women in Kisumu County, Kenya: A quasi-experimental study. *Pan African Medical Journal* 2018;29 (no pagination)(11) doi: http://dx.doi.org/10.11604/pamj.2018.29.11.13596

48. Rogow D. Living up to their name: Profamilia takes on gender-based violence. In: Rogow D, ed. Quality/Calidad/Qualité. https://pdfs.semanticscholar.org/e31a/25ccf1420d3c63c1dbfbc99000318e27ca1b.pdf: Population Council, 2006:35.

49. Samandari G, Delamou A, Traore P, et al. Integrating Intimate Partner Violence Screening and Counseling in a Family Planning Clinic: Evaluation of a Pilot Project in Conakry, Guinea. *African Journal of Reproductive Health* 2016;20(2):86-93.

50. Sapkota D, Baird K, Saito A, et al. Antenatal-Based Pilot Psychosocial Intervention to Enhance Mental Health of Pregnant Women Experiencing Domestic and Family Violence in Nepal. *J Interpers Violence* 2020:886260520948151. doi: 10.1177/0886260520948151 [published Online First: 2020/08/20]

51. Sapkota D, Baird K, Saito A, et al. 'We don't see because we don't ask': Qualitative exploration of service users' and health professionals' views regarding a psychosocial intervention targeting pregnant women experiencing domestic and family violence. *PLoS One* 2020;15(3):e0230069. doi: 10.1371/journal.pone.0230069 [published Online First: 2020/03/10]

52. Sikkema KJ, Mulawa MI, Robertson C, et al. Improving AIDS Care After Trauma (ImpACT): Pilot Outcomes of a Coping intervention Among HIV-Infected Women with Sexual Trauma in South Africa. *AIDS & Behavior* 2018;22(3):1039-52. doi: 10.1007/s10461-017-2013-1

For peer review only

53. Sithole Z, Gombe NT, Juru T, et al. Evaluation of sexual and gender-based violence program in Harare City, Zimbabwe, 2016: a descriptive cross-sectional study. *Pan Afr Med J* 2018;31:200. doi: 10.11604/pamj.2018.31.200.14791 [published Online First: 2019/05/16]

54. Smith JR, Ho LS, Langston A, et al. Clinical care for sexual assault survivors multimedia training: a mixed-methods study of effect on healthcare providers' attitudes, knowledge, confidence, and practice in humanitarian settings. *Confl Health* 2013;7:14. doi: https://dx.doi.org/10.1186/1752-1505-7-14

55. Taghizadeh Z, Pourbakhtiar M, Ghasemzadeh S, et al. The effect of training problem-solving skills for pregnant women experiencing intimate partner violence: a randomized control trial. *Pan African Medical Journal* 2018;30:11. doi: 10.11604/pamj.2018.30.79.14872

56. Turan JM, Hatcher AM, Odero M, et al. A Community-Supported Clinic-Based Program for Prevention of Violence against Pregnant Women in Rural Kenya. *AIDS Res Treat* 2013;2013:736926. doi: https://dx.doi.org/10.1155/2013/736926

57. Undie C-C, Maternowska M, Mak'anyengo M, et al. Is routine screening for intimate partner violence feasible in public health care settings in Kenya? *Journal of Interpersonal Violence* 2016;31(2):282-301. doi: http://dx.doi.org/10.1177/0886260514555724

58. Vakily M, Noroozi M, Yamani N. Comparing the effect of group-based and compact disk-based training on midwives' knowledge and attitude toward domestic violence in women of reproductive age. *Journal of Education and Health Promotion* 2017;6:70. doi: http://dx.doi.org/10.4103/jehp.jehp_44_16

59. Wagman JA, Gray RH, Campbell JC, et al. Effectiveness of an integrated intimate partner violence and HIV prevention intervention in Rakai, Uganda: analysis of an intervention in an existing cluster randomised cohort. *Lancet Glob Health* 2015;3(1):e23-33. doi: 10.1016/S2214-109X(14)70344-4 [published Online First: 2014/12/30]

60. Settergren SK, Mujaya S, Rida W, et al. Cluster randomized trial of comprehensive gender-based violence programming delivered through the HIV/AIDS program platform in Mbeya Region, Tanzania: Tathmini GBV study. *PLoS One* 2018;13(12):e0206074. doi: 10.1371/journal.pone.0206074 [published Online First: 2018/12/07]

61. Skivington K, Matthews L, Simpson SA, et al. A new framework for developing and evaluating complex interventions: update of Medical Research Council guidance. *BMJ* 2021;374:n2061. doi: 10.1136/bmj.n2061 [published Online First: 2021/10/02]

For peer review only

62. Arango D, Morton M, Gennari F, et al. Interventions to reduce or prevent violence against women and girls: a systematic review of reviews. Women's Voice, Agency and Participation Research Series, ed. Washington DC: World Bank 2014.

63. Jahanfar S, Howard LM, Medley N. Interventions for preventing or reducing domestic violence against pregnant women. *Cochrane Database of Systematic Reviews* 2014(11) doi: 10.1002/14651858.CD009414.pub3

64. Van Parys AS, Verhamme A, Temmerman M, et al. Intimate partner violence and pregnancy: a systematic review of interventions. *PLoS One* 2014;9(1):e85084. doi: 10.1371/journal.pone.0085084 [published Online First: 2014/02/01]

65. Turner DT, Riedel E, Kobeissi LH, et al. Psychosocial interventions for intimate partner violence in low and middle income countries: A meta-analysis of randomised controlled trials. *J Glob Health* 2020;10(1):010409. doi: 10.7189/jogh.10.010409 [published Online First: 2020/05/07]

66. Kerr-Wilson A, Gibbs A, E. MF, et al. What works to prevent violence against women and girls global programme. A rigorous global evidence review of interventions to prevent violence against women and girls. Pretoria: South Africa, 2020:96.

67. Tarzia L, Bohren MA, Cameron J, et al. Women's experiences and expectations after disclosure of intimate partner abuse to a healthcare provider: A qualitative meta-synthesis. *BMJ Open* 2020;10(11):e041339. doi: 10.1136/bmjopen-2020-041339 [published Online First: 2020/11/29]

68. Colombini M, Dockerty C, Mayhew SH. Barriers and Facilitators to Integrating Health Service Responses to Intimate Partner Violence in Low- and Middle-Income Countries: A Comparative Health Systems and Service Analysis. *Studies in Family Planning* 2017;48(2):179-200. doi: https://dx.doi.org/10.1111/sifp.12021

Figure 1. Flow diagram.

Figure 2. Process-oriented logic model of interventions in sexual and reproductive health services addressing violence against women in low- and middle- income countries.

For peer review only

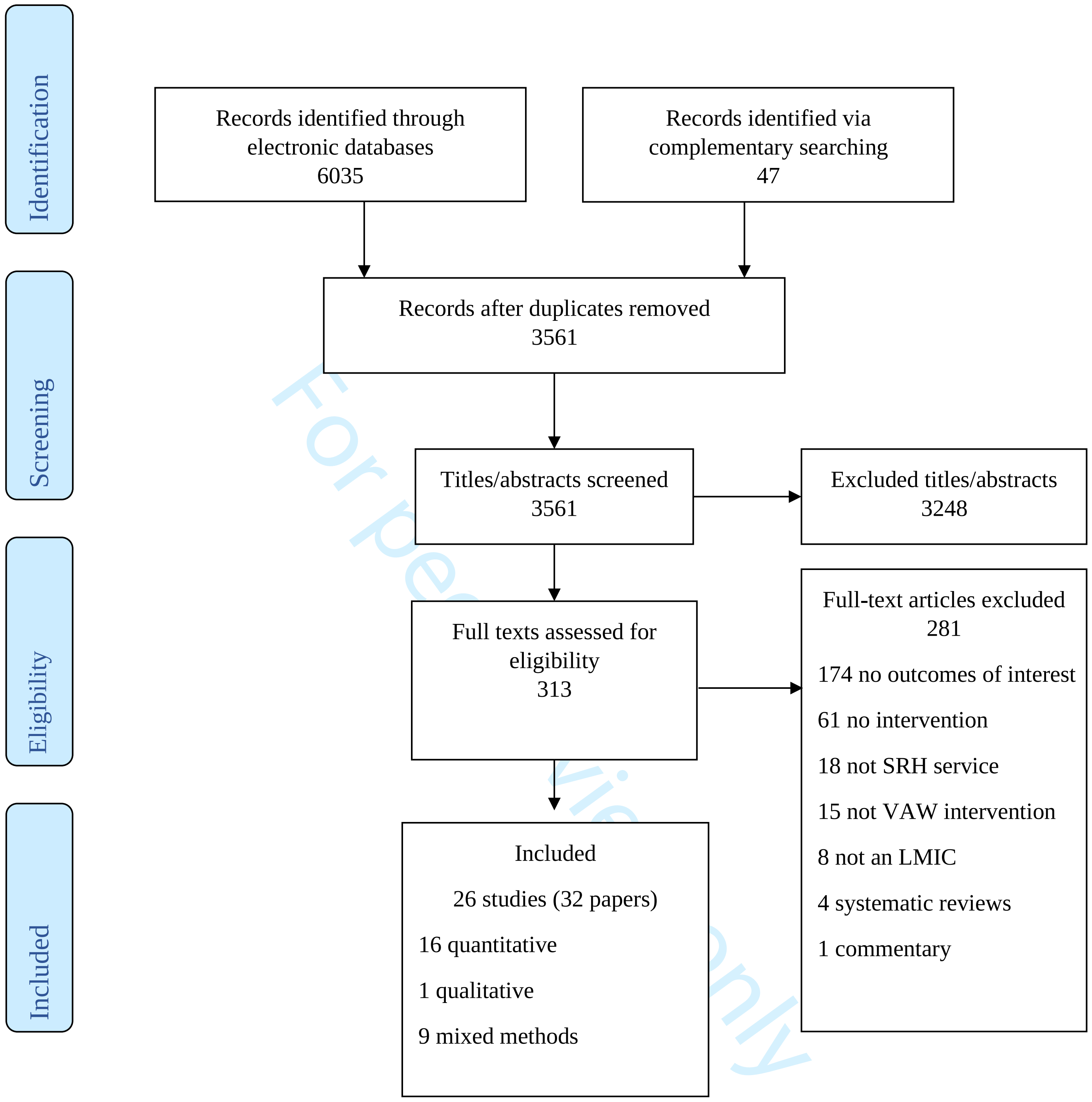

Figure 1. Flow diagram

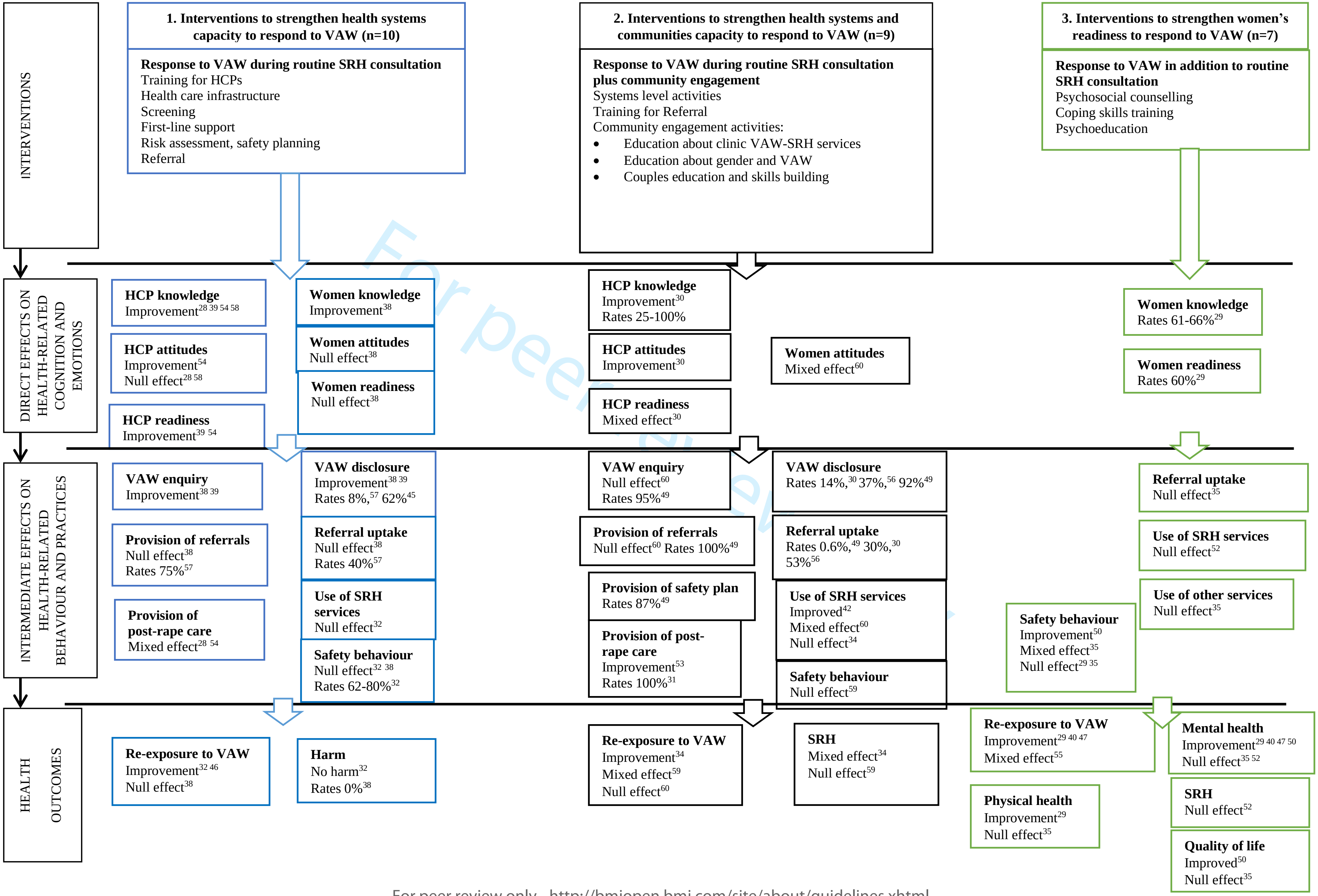
INTERVENTIONS
1. Interventions to strengthen health systems capacity to respond to VAW (n=10)
Response to VAW during routine SRH consultation
Training for HCPs
Health care infrastructure
Screening
First-line support
Risk assessment, safety planning
Referral
2. Interventions to strengthen health systems and communities capacity to respond to VAW (n=9)
Response to VAW during routine SRH consultation plus community engagement
Systems level activities
Training for Referral
Community engagement activities:
• Education about clinic VAW-SRH services
• Education about gender and VAW
• Couples education and skills building
3. Interventions to strengthen women's readiness to respond to VAW (n=7)
Response to VAW in addition to routine SRH consultation
Psychosocial counselling
Coping skills training
Psychoeducation
DIRECT EFFECTS ON HEALTH-RELATED COGNITION AND EMOTIONS
HCP knowledge Improvement[28 39 54 58]
HCP attitudes Improvement[54] Null effect[28 58]
HCP readiness Improvement[39 54]
Women knowledge Improvement[38]
Women attitudes Null effect[38]
Women readiness Null effect[38]
HCP knowledge Improvement[30] Rates 25-100%
HCP attitudes Improvement[30]
HCP readiness Mixed effect[30]
Women attitudes Mixed effect[60]
Women knowledge Rates 61-66%[29]
Women readiness Rates 60%[29]
INTERMEDIATE EFFECTS ON HEALTH-RELATED BEHAVIOUR AND PRACTICES
VAW enquiry Improvement[38 39]
Provision of referrals Null effect[38] Rates 75%[57]
Provision of post-rape care Mixed effect[28 54]
VAW disclosure Improvement[38 39] Rates 8%,[57] 62%[45]
Referral uptake Null effect[38] Rates 40%[57]
Use of SRH services Null effect[32]
Safety behaviour Null effect[32 38] Rates 62-80%[32]
VAW enquiry Null effect[60] Rates 95%[49]
Provision of referrals Null effect[60] Rates 100%[49]
Provision of safety plan Rates 87%[49]
Provision of post-rape care Improvement[53] Rates 100%[31]
VAW disclosure Rates 14%,[30] 37%,[56] 92%[49]
Referral uptake Rates 0.6%,[49] 30%,[30] 53%[56]
Use of SRH services Improved[42] Mixed effect[60] Null effect[34]
Safety behaviour Null effect[59]
Referral uptake Null effect[35]
Use of SRH services Null effect[52]
Use of other services Null effect[35]
Safety behaviour Improvement[50] Mixed effect[35] Null effect[29 35]
HEALTH OUTCOMES
Re-exposure to VAW Improvement[32 46] Null effect[38]
Harm No harm[32] Rates 0%[38]
Re-exposure to VAW Improvement[34] Mixed effect[59] Null effect[60]
SRH Mixed effect[34] Null effect[59]
Re-exposure to VAW Improvement[29 40 47] Mixed effect[55]
Physical health Improvement[29] Null effect[35]
Mental health Improvement[29 40 47 50] Null effect[35 52]
SRH Null effect[52]
Quality of life Improved[50] Null effect[35]

Supplementary file 1. Search strategy

Searched 20 August 18
Databases:
(VAW AND Interventions AND LMICs AND healthcare) less exclusions
See attached Medline strategy
Grey literature:
("violence against women" OR "intimate partner violence" "domestic violence" OR DVA OR IPV OR VAW) AND (intervention* or prevention OR trial*)

Search results (databases):
Medline/Premedline = 1464
Embase= 1403
Psycinfo= 594
Cochrane =61
Cinahl=314
IMEMR =5
Web of Science= 920
Popline= 880
Lilacs= 392
WHO RHL=2
Total= 6035
Total deduplicated =3514

Search grey literature:
UNFA=8
SVRI = 5
JPHIEGO =3
USAID =4
WHO (IRIS) SEARO =2
WHO (IRIS) EMRO =3
Google=1
Google Scholar=1
ClinicalTrials.gov =15
WORLD Bank
OTHER= 1
Total= 45
Total deduplicated against database search= 43

Grand Total (databases and grey lit) =3557

Database: Ovid MEDLINE(R) and Epub Ahead of Print, In-Process & Other Non-Indexed Citations and Daily <1946 to August 20, 2018>
Search Strategy:
----
1 rape/ or domestic violence/ or exp intimate partner violence/ or battered women/ or Gender-Based Violence/ (19658)
2 (violence/ or sex offenses/ or sexual harassment/ or homicide/ or physical abuse/ or coercion/ or crime victims/) and (female/ or women/ or spouses/ or marriage/ or Sexual partners/) (29381)
3 (violence/ or sex offenses/ or sexual harassment/ or homicide/ or physical abuse/ or coercion/ or crime victims/) and (female* or domestic or spous* or partner* or woman or women or married or marriage* or marital or husband* or wife or wives or boyfriend* or girlfriend* or gender-based or non-partner).tw. (13114)

For peer review only - http://bmjopen.bmj.com/site/about/guidelines.xhtml

4 ((sexual abuse or sexual harassment or sexual coercion or violent or violence or assault* or beat or beating or batter* or rape* or sex offense* or sexual offense*) adj4 (female* or domestic or spous* or partner* or woman or women or married or marriage* or marital or husband* or wife or wives or boyfriend* or girlfriend* or gender-based or non-partner)).tw. (18139)
5 (IPV or DVA).tw. (5675)
6 (VAW or date rape).tw. (309)
7 ((woman or women) adj3 relationship* adj3 abus*).tw. (90)
8 ((birth control or fertility control or reproductiv* or contraceptiv* or contraception) adj3 (sabotag* or coerc*)).tw. (105)
9 or/1-8 (52401)
10 ((prevent* or intervention* or eliminat* or program* or approach or approaches or trial* or response* or effective or effectiveness or identify or efficacy or what works or outcome* or treatment* or therap* or identification) adj12 (violent or violence or rape* or DVA or IPV or VAW or harassment or sexual offense* or sex offense* or abus* or assault* or beating or beat or coerc* or female* or domestic or spous* or partner* or woman or women or married or marriage* or marital or husband* or wife or wives or boyfriend* or girlfriend* or gender-based or non-partner)).tw. (398870)
11 rape/pc or sex offenses/pc or domestic violence/pc or exp intimate partner violence/pc or battered women/pc or Gender-Based Violence/pc or Sexual Harassment/pc (4812)
12 (psychosocial support or psychological support or education* or training or home visit* or advocacy).tw. (776643)
13 secondary prevention/ or tertiary prevention/ (18314)
14 ((questioning or interviewing or empower*) adj3 (method* or technique*)).tw. (1434)
15 (patient adj3 information).tw. (16715)
16 ((poster* or information or pamphlet* or leaflet*) adj3 (provision or provide*)).tw. (172170)
17 counsel?ing.ti,ab. (81732)
18 exp counseling/ (40659)
19 exp Clinical Trials as Topic/ (316975)
20 exp clinical trial/ (805706)
21 "Controlled Before-After Studies"/ (348)
22 "outcome and process assessment (health care)"/ or "process assessment (health care)"/ (29451)
23 ((program* or process* or service) adj3 evaluation*).tw. (22711)
24 (pretest* or pre-test* or posttest* or post-test* or pre-intervention* or preintervention* or postintervention* or post-intervention*).tw. (52054)
25 (pre* adj12 post*).tw. (579088)
26 ("before and after" or before-after).tw. (243968)
27 or/10-26 (2980515)
28 9 and 27 (24093)
29 Developing Countries/ (70541)
30 (developing countr* or emerging econom* or third world).tw. (55717)
31 ((low or middle) adj4 income countr*).ti,ab. (16763)
32 LMIC*.ti,ab. (2703)
33 (Afghanistan or Benin or Burkina Faso or Burundi or Cambodia or Central Africa or Chad or Comoros or Congo or Eritrea or Ethiopia or Gambia or Guinea or Bissau or Haiti or North Korea or Liberia or Madagascar or Malawi or Mali or Mozambique or Nepal or Niger or Rwanda or Sierra Leone or Somalia or Tanzania or Togo or Uganda or Zimbabwe).mp. (289340)
34 (Armenia or Bangladesh or Bhutan or Bolivia or Cabo Verde or Cameroon or Cote d'Ivoire or Ivory Coast or Djibouti or Egypt or El Salvador or Georgia or Ghana or Guatemala or Guyana or Honduras or India or Indonesia or Kenya or Kiribati or Kosovo or Kyrgyz* or Lao or Laos or Lesotho or Mauritania or Micronesia or Moldova or Mongolia or Morocco or Myanmar or Nicaragua or Nigeria or Pakistan or Philippines or Samoa or Sao Tome or

For peer review only - http://bmjopen.bmj.com/site/about/guidelines.xhtml

Principe or Senegal or Solomon Islands or Sri Lanka or Sudan or Swaziland or Syria* or Tajikistan or Timor Leste or Ukraine or Uzbekistan or Vanuatu or Vietnam or West Bank or Gaza or Yemen or Zambia).mp. (392622)
35 (Albania or Angola or Argentina or Panama or Tunisia or Palau or Tunisia or Herzegovina or Fiji or Namibia or Algeria or Gabon or Nauru or Grenada or Paraguay or Peru or Azerbaijan or Grenadines or Romania or Belarus or Iran or Russia* or Belize or Iraq or Bosnia or Jamaica or Serbia).mp. (189750)
36 (Botswana or Jordan or South Africa or Brazil or Kazakhstan or Saint Lucia or St Lucia or Bulgaria or Lebanon or Saint Vincent or St Vincent or China or Libya or Suriname or Colombia or Macedonia or Thailand or Costa Rica or Malaysia or Tonga).mp. (435209)
37 (Cuba or Maldives or Turkey or Dominica* or Marshall Islands or Turkmenistan or Mauritius or Tuvalu or Mexico or Venezuela or Ecuador or Montenegro).mp. (122538)
38 or/29-37 (1403313)
39 28 and 38 (4702)
40 exp maternal health services/ or exp reproductive health services/ or family planning services/ (67984)
41 exp pregnancy/ or exp pregnancy trimesters/ or pregnant women/ or peripartum period/ or exp pregnancy complications/ or exp fetal therapies/ or exp Obstetric surgical procedures/ or exp postpartum period/ or obstetric nursing/ or midwifery/ (914606)
42 exp maternal-child nursing/ (5549)
43 (adolescent health services/ or community mental health services/ or community health services/ or rural health services/ or rural nursing/ or family health/ or adolescent health/ or exp primary health care/ or exp general practice/ or general practitioners/ or physicians, family/) and (women or woman or reproductive or sexual health* or "STI" or STD* or "STIS" or contracept* or abortion or childbirth or pregnan*).mp. (26039)
44 reproductive medicine/ or gynecology/ or obstetrics/ or "Obstetrics and Gynecology Department, Hospital"/ (35849)
45 ((sexual or reproductive) adj3 (education or healthcare or care or service* or program* or clinic*)).mp. (12944)
46 ((sexual or reproductive) adj3 (education or health* or care)).jn,in. (14583)
47 ((pregnan* or birth or childbirth or midwife* or midwive* or " mother and baby" or obstetric* or maternal or maternity or postpartum or antepartum or postnatal or post-natal or ante-natal or antenatal or prenatal or pre-natal or perinatal or peri-natal or contraception or contraceptiv* or abortion or fertility or gynae* or gyne* or STD* or "STI" or "STIS" or sexually transmitted or PMS or premenstrual syndrome) adj3 (care or healthcare or clinic* or service* or treatment*)).tw. (109386)
48 (cervical adj2 (smear* or screening)).tw. (11930)
49 vaginal smears/ or papanicolaou test/ (22149)
50 exp Sexually Transmitted Diseases/di, pc, rh, th (103211)
51 exp Women's Health/ (26647)
52 exp Menstruation Disturbances/di, pc, rh, th (5369)
53 ((woman* or women*) adj3 health*).jn,in,mp. (106372)
54 family planning*.jn,mp,in. (50733)
55 exp "diagnostic techniques, obstetrical and gynecological"/ (124519)
56 reproductive health/ or sexual health/ (2688)
57 or/40-56 (1238548)
58 39 and 57 (1822)
59 letter/ (997536)
60 editorial/ (466054)
61 news/ (190965)
62 exp historical article/ (382354)
63 Anecdotes as topic/ (4721)
64 comment/ (730950)
65 (letter or editorial or comment*).ti. (160815)
66 exp animals/ not humans/ (4489180)

For peer review only - http://bmjopen.bmj.com/site/about/guidelines.xhtml

67 exp Animals, Laboratory/ (820540)
68 exp Animal Experimentation/ (8786)
69 exp Models, Animal/ (516652)
70 exp rodentia/ (3047766)
71 (rat or rats or mouse or mice or rodent* or animal*).ti. (1394667)
72 or/59-71 (7516664)
73 58 not 72 (1776)
74 limit 73 to yr="2005 -Current" (1464)

For peer review only

For peer review only - http://bmjopen.bmj.com/site/about/guidelines.xhtml

Supplementary file 2. Characteristics of included studies by intervention category and level of evidence

Study, publication	Country	Setting	Sample characteristics	Intervention (n) vs comparison (n)	Design/	Follow up	Primary outcomes of interest for this review	Secondary outcomes of interest for this review
**Response to VAW during routine SRH consultation (n=10)**								
Vakily 2017[58]	Iran	Antenatal clinic, 32 outpatient health centres	HCPs (midwives)	2-hour HCP training computer assisted (35) vs face-to-face (35)	RCT	2 months		Knowledge and attitudes about DV
Brown 2018[32]	South Africa	HIV testing and counselling, community, NGO	HIV positive women 18+	7-minute integrated HIV-IPV consultation over phone (166) vs standard care (83)	RCT	1 month	IPV upon partner notification of serostatus, harm	Perceived safety, safety behaviours, access to HIV treatment
*Haberland 2016[38]	Kenya	HIV testing in antenatal clinic, hospital with GBV centre	HCPs (HIV testing counsellors) Pregnant women 15-49	HCP training and ongoing support, 29-minute integrated HIV-IPV consultation, referral to IPV counsellor in ANC clinic (337) vs standard care (351)	RCT Nested mixed-method process evaluation	1 month	Any IPV, harm	IPV screening, referrals Women's knowledge, attitudes, self-esteem, perceived intervention effect, HIV care Intervention acceptability
Abeid 2016[28]	Tanzania	Post-rape care service, 5 health centres and referral hospitals	HCPs (doctors, nurses, assistant medical/clinical officers)	5-day training, guidelines, infrastructure improvement (100) vs minimal intervention (53)	Controlled before-after	12 months		Knowledge and attitudes about sexual violence and post-rape care Provision of post-rape care
Jayatilleke 2015[39]	Sri Lanka	Antenatal clinic, community	HCPs (midwives)	4-day training, handbook, external referral (408)	Uncontrolled before-after	6 months		Knowledge, practices, responsibility, readiness for identifying and responding to IPV, provision of referrals
Matseke 2013[46]	South Africa	HIV testing and counselling in antenatal clinic, 16 primary health care clinics	Pregnant women 18+	HPC training, 30-minute integrated ANC-IPV consultation, external referral (160)	Uncontrolled before-after	3 months	Perceived risk of becoming a victim of femicide	
Smith 2013[54]	Kenya, Ethiopia, Jordan, Democratic Republic of Congo	Post-rape care service, 35 humanitarian settings, NGO	HCPs (doctors, nurses, midwives)	4-day training, infrastructure improvement (106)	Uncontrolled before-after Qualitative study	3 months		Attitudes, knowledge, skills on sexual violence and post-rape care, provision of post-rape care
Laisser 2011[45]	Tanzania	Antenatal clinic, hospital	HCPs (clinical/medical	HCP training (39), infrastructure improvement, integrated ANC-IPV	Cross sectional	3 weeks		Intervention acceptability

For peer review only

**Study, publication**	**Country**	**Setting**	**Sample characteristics**	**Intervention (n) vs comparison (n)**	**Design/**	**Follow up**	**Primary outcomes of interest for this review**	**Secondary outcomes of interest for this review**
			officers, nursing officers) Women 18+	consultation (102), external referral	Qualitative study			
Undie 2016[57]	Kenya	HIV testing in antenatal clinic, hospital with GBV centre	Women	HCP training, integrated HIV-IPV consultation (1210), assisted referral to on-site GBV centre	Cross sectional Qualitative study	7 months		IPV screening, referrals Intervention acceptability
Cristofides 2010[33]	South Africa	HIV testing and counselling, primary health care clinic	HCPs (lay counsellors) Women	HCP training (16), integrated HIV-IPV consultation (35), external referral	Qualitative study	2 weeks		Intervention acceptability
**Response to VAW during routine SRH consultation plus community engagement (n=9)**								
Cockcroft 2019[34]	Nigeria	Universal home visits, 4 communities	Pregnant women 14-49	HCP training, infrastructure improvement, integrated DV-universal home visits that discussed domestic violence, heavy work in pregnancy, ignorance of danger signs, and lack of spousal communication with pregnant women (1837) and their partners vs delayed intervention (1853)	Cluster RCT	12 months	Physical DV, pregnancy delivery, postnatal complications	Use of SRH services
Settergren 2018[60]	Tanzania	HIV/AIDS services, hospital, and health centre	Women 15-49	Systems level activities, HCP training, infrastructure improvement, integrated-HIV-GBV consultation, onsite and external referral, community, and couple education (6 facilities, 656 women) vs standard care (6 facilities, 643 women)	Cluster RCT	28 months	Any IPV	Provision of services to IPV positive patients
Wagman 2015[59]	Uganda	HIV testing and counselling, community	Women 15-49	HCP training, integrated HIV-IPV consultation, onsite referral (6 facilities, 1812 women) vs standard care (5 facilities, 2127 women)	Cluster RCT	4 years and 7 months	Physical, emotional, sexual IPV, HIV incidence	Risk behaviours and HIV disclosure
*Bott 2014[30 37 48]	Dominican Republic, Peru, Venezuela	3 family planning clinics, NGO	HCP (doctors, nurses, midwives, counsellors, social workers, psychologists, receptionists) Women 12+	Systems level activities, HCP training and ongoing support, infrastructure improvement, integrated GBV-family planning consultation, referral to onsite GBV specialist (4 clinics)	Uncontrolled before-after Qualitative study	3 years		HCPs attitudes, knowledge, readiness for identifying and responding to GBV Intervention acceptability

For peer review only - http://bmjopen.bmj.com/site/about/guidelines.xhtml

Study, publication	Country	Setting	Sample characteristics	Intervention (n) vs comparison (n)	Design/	Follow up	Primary outcomes of interest for this review	Secondary outcomes of interest for this review
Kim 2007[42 43]	South Africa	Post-rape care service, hospital	Survivors of sexual violence	Systems level activities, 2-day HCP training (334), infrastructure improvement, community education on post-rape care	Uncontrolled before-after	No information		Use, quality, and cost of post-rape care service
Bress 2018[31]	Democratic Republic of Congo	Post-rape care service, 12 primary care clinics and referral hospital	Survivors of sexual violence 12+	HCP training and ongoing support, infrastructure improvement, community education on post-rape care (13 sites, 2081 survivors)	Cross-sectional	4 years		Provision of post-rape kit
Samandari 2016[36 49]	Guinea	Family planning clinic	HCPs (nurse, midwife, counsellor, support/admin staff) Women	System level activities, 7-day HCP training and ongoing support (4), integrated family planning-IPV consultation (171), external referral, community education	Cross-sectional Qualitative study	4 months		IPV identification, safety planning, referrals Intervention acceptability
Sithole 2018[53]	Zimbabwe	Comprehensive post-rape care service, 8 policlinics, NGO	HCPs (doctors, nurses, managers) Survivors of sexual violence	HCP training (80), infrastructure improvement, post-rape care (1669), community education on post-rape care	Cross sectional service evaluation	4 years		HCPs knowledge about post-rape care Provision of post-rape care
Turan 2013[56]	Kenya	Antenatal clinic, primary health care clinic	HCPs (all clinic staff including admin, community volunteers, lay health workers) Pregnant women	40-hour HCP training, integrated ANC-GBV consultation (134), assisted external referral, community education	Cross sectional Qualitative study	5 months		GBV identification, referrals Intervention acceptability
**Response to VAW in addition to routine SRH consultation (n=7)**								
Cripe 2010[35]	Peru	Antenatal clinic, hospital	Pregnant women 18-45 with IPV experience	1* 30-minute psychosocial counselling session by social worker, resource card, external referral (110) vs minimal intervention (110)	RCT	Prenatal appointment to 1 week after delivery	Quality of life	Safety behaviours, use of community resources
Khalili 2019[40 41]	Iran	Antenatal clinic, University health centers	Pregnant women 20+ with IPV experience	4*90-minute psychoeducational sessions by counsellor (50) vs standard care (50)	RCT	2 months	Verbal and physical IPV, psychological distress	
Mutisya 2018[47]	Kenya	Antenatal clinic, 12 primary health care clinics	Pregnant women 18-45 with IPV experience	1-3*30-35-minute psychosocial counselling sessions by researcher, risk assessment, safety planning, resource card, external referral	RCT	6 months	Physical, emotional, severe combined IPV and harassment, depression	

For peer review only

Study, publication	Country	Setting	Sample characteristics	Intervention (n) vs comparison (n)	Design/	Follow up	Primary outcomes of interest for this review	Secondary outcomes of interest for this review
				(141) vs minimal intervention (142)				
Sapkota 2020[50 51]	Nepal	Antenatal clinic, hospital	Pregnant women 18+ with DV experience	1*35-45-minute psychosocial session by counsellor, resource card, contact details of the counsellor (70) vs minimal intervention (70)	RCT Nested qualitative study	Prenatal appointment to 6 weeks after delivery	Depression, anxiety, quality of life	Self-efficacy, safety behaviours, social support Intervention acceptability
Sikkema 2018[44 52]	South Africa	HIV testing and treatment, primary health care clinic	HIV positive women 18+ with experience of sexual violence	4 individual and 3 group*90-minute psychosocial training sessions by trained lay provider (32) vs standard care (32)	RCT Nested qualitative study	6 months	PTSD	Coping strategies, engagement with HIV treatment Intervention acceptability
Tanghizaden 2018[55]	Iran	Antenatal clinic, 16 health centres	Pregnant women with IPV experience	4*90-minute psychosocial training sessions on problem-solving skills by researcher (125) vs standard care (132)	RCT	3 months	Physical, psychological, sexual IPV	
Arora 2019[29]	India	Antenatal clinic, 2 hospitals	Pregnant women with DV experience	≥2*30-45-minute psychosocial sessions by counsellor (155)	Uncontrolled before-after	First prenatal appointment to 6 weeks after delivery	Physical, emotional, financial DV, physical health, emotional health	Knowledge and attitudes about DV, coping behaviours

Note. * grey literature. NGO non-governmental (third sector) organisation. RCT randomised controlled trial. HCP health care providers. IPV intimate partner violence. DV domestic violence. GBV gender-based violence. HIV human immunodeficiency viruses. PTSD posttraumatic stress disorder. SRH sexual and reproductive

For peer review only

Online supplementary file 3. Quality appraisal

Risk of bias in randomised controlled trials

Study ID	Bias arising from the randomization process	Bias due to deviations from the intended interventions (assignment)	Bias due to deviations from the intended interventions (adherence)	Bias due to missing outcome data	Bias in measurement of the outcome	Bias in selection of the reported result	Overall risk of bias
Brown 2018[32]	Low	Some concerns	High	Low	Low	Low	High
Cockcroft 2019[34]	Low	Low	Low	Low	Low	Low	Low
Cripe 2013[35]	Low	Low	Low	Low	Some concerns	Some concerns	Some concerns
Haberland 2016[38]	Some concerns	Low	High	Low	Some concerns	Some concerns	High
Khalili 2020[40]	Low	High	High	Low	High	Low	High
Mutisya 2018[47]	Low	High	High	Low	High	Some concerns	High
Sapkota 2020[50]	Low	Low	Low	Low	Low	Low	Low
Settergren 2018[60]	Low	Low	High	Low	High	Low	High
Sikkema 2018[52]	Low	Low	Low	High	Some concerns	Some concerns	High
Tanghizadeh 2018[55]	Low	Low	Low	Low	Low	Some concerns	Some concerns
Vakily 2017[58]	Some concerns	Some concerns	High	High	Low	Some concerns	High
Wagman 2015[59]	Some concerns	High	Some concerns	Low	High	Some concerns	High

Risk of bias (EPOC criteria) in controlled before-after studies

Study ID	Was the allocation sequence adequately generated?	Was the allocation adequately concealed?	Were baseline outcome measurements similar?	Were baseline characteristics similar?	Were incomplete outcome data adequately addressed?	Was knowledge of the allocated interventions adequately prevented during the study?	Was the study adequately protected against contamination ?	Was the study free from selective outcome reporting?	Was the study free from other sources of bias?	Overall risk of bias
Abeid 2016[28]	No	No	Yes	Yes	Yes	Unclear	Unclear	Yes	Unclear	High

Risk of bias (EPOC criteria) in studies without a control group

Study ID	Was the intervention independent of other changes?	Was the shape of the intervention effect pre-specified?	Was the intervention unlikely to affect data collection?	Was knowledge of the allocated interventions adequately prevented during the study?	Were incomplete outcome data adequately addressed?	Was the study free from selective outcome reporting?	Was the study free from other risk of bias?	Overall risk of bias
Arora 2019[29]	No	Yes	Yes	No	Yes	No	Unclear	High
Bott 2004[30]	Unclear	Unclear	Yes	No	No	Unclear	Unclear	High
Bress 2019[31]	No	No	No	No	Unclear	Yes	Unclear	High

Study ID	Was the intervention independent of other changes?	Was the shape of the intervention effect pre-specified?	Was the intervention unlikely to affect data collection?	Was knowledge of the allocated interventions adequately prevented during the study?	Were incomplete outcome data adequately addressed?	Was the study free from selective outcome reporting?	Was the study free from other risk of bias?	Overall risk of bias
Jayatilleke 2015[39]	Unclear	Yes	Yes	No	Unclear	Yes	Unclear	High
Kim 2007[42]	No	Yes	Yes	No	Unclear	No	No	High
Laisser 2011[45]	No	No	No	No	Unclear	Yes	Unclear	High
Matseke 2013[46]	Unclear	Yes	Yes	No	No	Yes	No	High
Samandari 2016[49]	No	Yes	Yes	No	Yes	Yes	Unclear	High
Sithole 2018[53]	Unclear	No	No	No	Unclear	No	Unclear	High
Smith 2013[54]	No	Yes	Yes	Unclear	Yes	Yes	No	High
Turan 2013[56]	Unclear	No	No	No	Unclear	Yes	Unclear	High
Undie 2016[57]	No	No	No	No	Unclear	Unclear	Unclear	High

Quality appraisal of qualitative studies

CASP signalling questions	Bott 2004[30]	Christofides 2010[33]	Haberland 2016[38]	Laisser 2011[45]	Samandari 2016[49]	Sapkota 2020[51]	Sikkema 2018[44]	Smith 2013[54]	Turan 2013[56]	Undie 2016[57]
1. Interprets subjective experiences?	Yes	Yes	Yes	Yes	Yes	Yes	Yes	Yes	Yes	Yes
2. Right methodology?	Yes	Yes	Yes	Yes	Yes	Yes	Yes	Yes	Yes	Yes
3. Appropriate design?	Yes	Yes	Yes	Yes	Yes	Yes	Yes	Yes	Yes	Yes
4. Design justified?	Yes	No	No	Yes	No	Yes	Yes	No	No	No
5. Ethical issues considered?	No	No	Yes	Yes	Yes	Yes	Yes	Yes	Yes	Yes
6. Credibility established?	Yes	No	No	Yes	Yes	Yes	Yes	Yes	Yes	Yes
7. Transferability established?	No	Yes	No	Yes	Yes	Yes	Yes	Yes	No	No
8. Purpose established?	Yes	Yes	Yes	Yes	Yes	Yes	Yes	Yes	No	Yes
9. Recruitment appropriate?	Yes	Yes	Yes	Yes	Yes	Yes	Yes	Yes	Yes	Yes
10. Selection of participants explained?	No	Yes	Yes	Yes	Yes	Yes	Yes	No	Yes	No
11. Participants appropriate?	No	No	No	Yes	Yes	No	Yes	Yes	Yes	No
12. Discussed recruitment?	No	Yes	No	No	No	No	Yes	No	No	No
13. Justified setting?	Yes	Yes	Yes	Yes	Yes	Yes	Yes	Yes	Yes	No
14. How data were collected?	Yes	Yes	Yes	Yes	Yes	Yes	Yes	Yes	Yes	Yes
15. Justified data collection method?	No	No	Unsure	Yes	No	Yes	Yes	No	No	No

CASP signalling questions	Bott 2004[30]	Christofides 2010[33]	Haberland 2016[38]	Laisser 2011[45]	Samandari 2016[49]	Sapkota 2020[51]	Sikkema 2018[44]	Smith 2013[54]	Turan 2013[56]	Undie 2016[57]
16. Described data collection method?	No	Yes	Yes	Yes	Yes	Yes	Yes	Yes	Yes	Yes
17. Form of data clear?	No	Yes	Yes	Yes	Yes	Yes	Yes	Yes	Yes	Yes
18. Described how data were reduced/transformed for analysis?	No	No	No	Yes	Yes	Yes	Yes	Yes	Yes	Yes
19. Discussed interpretation of findings?	Yes	No	No	Yes	Yes	Yes	Yes	No	Yes	Yes
20. Ensured neutrality?	No	No	No	No	Yes	Yes	Yes	No	Yes	No
**Total (Yes/No/Unsure)**	**10/10/0**	**12/8/0**	**11/8/1**	**18/2/0**	**17/3/0**	**18/2/0**	**20/0/0**	**14/6/0**	**15/5/0**	**12/8/0**

For peer review only

Supplementary file 4. Effects and outcomes of interventions on response to VAW during routine SRH consultation

**Outcomes**	**Results (95% CI, p)**	**Effect (95% CI)**	**N of participants (design/follow up)**	**Study**	**Risk of bias**	**Direction of effect/interpretation**
**Direct effect on health-related cognition and emotions**						
**HCP knowledge about VAW, relevant procedures (n=4)**						
Mean (SD) score of knowledge about domestic violence, post-intervention	Group training = 16.1 (1.9)<br>CD training = 17.7 (1.1)<br>p<0.001	-	35 HCPs (RCT/2 month)	Vakily 2017[58]	High	2-hour CD training improved HCP knowledge about domestic violence more than group training
Change in proportion with correct knowledge on sexual violence	Intervention = 31.4%<br>Control = -22.3%	Net effect = 53.7% (32.2; 75.1)	HCPs (CBA/12 months	Abeid 2016[28]	High	5-day training, guidelines, infrastructure improvement improved HCPs knowledge about sexual violence
Median (IQR) score of knowledge about IPV	Pre-intervention = 0.62 (0.43-0.81)<br>Post-intervention = 0.88 (0.82-0.94)<br>p<0.001	-	408 HCPs (UBA/6 months)	Jayatilleke 2015[39]	High	4-day training, handbook, external referral improved HCP knowledge about IPV
Mean (95% CI) score of knowledge in providing care to sexual assault survivors	Pre-intervention = 49.09 (45.57; 51.34)<br>Post-intervention = 61.59 (59.04; 64.42)	MD = 12.50 (10.29; 16.24)	106 HCPs (UBA/3 months)	Smith 2013[54]	High	4-day training, infrastructure improvement contributed towards improved HCP knowledge about providing clinical care to survivors of sexual violence
**HCP attitudes about VAW (n=3)**						
Mean (SD) score of attitudes about domestic violence, post-intervention	Group training = 46.9 (4.9)<br>CD training = 45.4 (6.4)<br>p = 0.3	-	35 HCPs (RCT/2 month)	Vakily 2017[58]	High	Neither group nor CD 2-hour training had effect on HCP attitudes about domestic violence
Mean (95% CI) score of attitudes about sexual violence	Pre-intervention = 71.76 (66.79; 73.14)<br>Post-intervention = 77.20 (72.53; 78.34)	MD = 5.44 (1.89; 8.98)	106 HCPs (UBA/3 months)	Smith 2013[54]	High	4-day training, infrastructure improvement contributed towards improved attitudes about sexual violence
Change in proportion with accepting attitude towards sexual violence	Intervention = -4.1%<br>Control = 6.8%	Net effect = -10.9% (-27.2; 5.5)	HCPs (CBA/12 months	Abeid 2016[28]	High	5-day training, guidelines, infrastructure improvement had no effect on HCP attitudes about sexual violence
**HCP readiness for identifying and responding to VAW (n=2)**						
Median (IQR) score of perceived barriers to IPV identification and response	Pre-intervention = 2.43 (2.14-3.14)<br>Post-intervention = 1.14 (1.14-1.28)<br>p<0.001		408 HCPs (UBA/6 months)	Jayatilleke 2015[39]	High	4-day training, handbook, external referral reduced HCP perceived barriers to identifying and responding to IPV
Median (IQR) score of perceived responsibilities to identify and respond to IPV	Pre-intervention = 3.20 (2.80-3.95)<br>Post-intervention = 4.60 (4.20-4.80)<br>p<0.001					4-day training, handbook increased HCP perceived responsibility and self-confidence to identify and respond to IPV

For peer review only - http://bmjopen.bmj.com/site/about/guidelines.xhtml

Outcomes	Results (95% CI, p)	Effect (95% CI)	N of participants (design/follow up)	Study	Risk of bias	Direction of effect/interpretation
Median (IQR) score of self-confidence to identify and respond to IPV	Pre-intervention = 1.81 (1.38-2.12)<br>Post-intervention = 2.75 (2.62-2.88)<br>p<0.001					
Mean (95% CI) score of HCPs' confidence in providing care to sexual assault survivors	Pre-intervention = 58.16 (53.86; 63.90)<br>Post-intervention = 72.66 (66.21; 74.30)	MD = 14.50 (8.22; 20.77)	106 HCPs (UBA/3 months)	Smith 2013[54]	High	4-day training, infrastructure improvement contributed towards improved confidence in providing clinical care to sexual assault survivors
**Women knowledge about VAW (n=1)**						
Mean difference (95% CI) in women's IPV knowledge score, post-intervention	-	MD = 0.16<br>Crude β=0.176 (0.02; 0.033)<br>Adjusted β=0.155 (0.00-0.31)	337 women (RCT/1 month)	Haberland 2016[38]	High	HCP training and ongoing support, 29-minute integrated HIV-IPV consultation, referral to IPV counsellor in ANC clinic improved knowledge about IPV and women's rights among pregnant women
Mean (SD) score of learning about women's rights in relationship	Intervention = 2.6 (1.1)<br>Control = 2.0 (1.0)<br>p<0.0001	-				
**Women attitudes about VAW (n=1)**						
Proportion (n) who justified wife beating, post-intervention	Intervention = 18.3% (49/268)<br>Control = 21.8% (58/267)<br>p=0.33	-	337 women (RCT/1 month)	Haberlan 2016[38]	High	HCP training and ongoing support, 29-minute integrated HIV-IPV consultation, assisted onsite referral had no effect on attitudes about IPV among pregnant women
**Women readiness for addressing VAW (n=1)**						
Proportion (n) who felt more confident in how deserve to be treated, post-intervention	Intervention = 82% (73/107)<br>Control = 71.6% (73/134)<br>p=0.12	-	337 women (RCT/1 month)	Haberlan 2016[38]	High	HCP training and ongoing support, 29-minute integrated HIV-IPV consultation, assisted onsite referral had no effect on self-confidence among pregnant women
**Intermediate effects on health-related behaviour and practices**						
**VAW enquiry rate (n=2)**						
Proportion (n) screened for IPV, post-intervention	Intervention = 76% (81/107)<br>Control = 22% (29/134)<br>p<0.0001	-	337 women (RCT/1 month)	Haberland 2016[38]	High	HCP training and ongoing support, 29-minute integrated HIV-IPV consultation, assisted onsite referral increased IPV enquiry rate
Proportion (n) who discussed IPV	Pre-intervention = 67.3% (201)<br>Post-intervention = 96.5% (387)<br>p<0.01	-	408 HCPs (UBA/6 months)	Jayatilleke 2015[39]	High	4-day HCP training, handbook, external referral increased IPV enquiry rate
**Provision of referrals to VAW services (n=2)**						
Proportion (n) referred to GBV centre of those disclosed, post-intervention	Intervention = 56% (19/34)<br>Control = 33% (3/9)<br>p=0.28	-	337 women (RCT/1 month)	Haberland 2016[38]	High	HCP training and ongoing support, 29-minute integrated HIV-IPV consultation, referral to IPV counsellor in ANC clinic had no effect on referral rate

Outcomes	Results (95% CI, p)	Effect (95% CI)	N of participants (design/follow up)	Study	Risk of bias	Direction of effect/interpretation
Proportion (n) referred to the medical officer or Heath/IPV services	Pre-intervention = 6.5% (13)<br>Post-intervention = 22.4% (87)<br>p not reported	-	408 HCPs (UBA/6 months)	Jayatilleke 2015[39]	High	4-day training, handbook, external referral had no effect on referral rates to external IPV services
Proportion (n) referred to GBV centre of those disclosed, post-intervention	75% (73/95)	-	1210 women (Cross sectional/7 months)	Undie 2016[57]	High	HCP training, integrated IPV-HIV consultation, assisted referral contributed towards 75% referral rate to on-site GBV centre
**Provision of post-rape care (n=2)**						
Change in proportion who used a rape kit	Intervention = 59.6%<br>Control = -4.5%	Net effect = 64.1% (46.7; 81.5)	100 HCPs (CBA/12 months)	Abeid 2016[28]	High	5-day training, guidelines, infrastructure improvement contributed towards improvement on 10 out of 18 indicators of post-rape care
Change in proportion who gave prophylactic treatment for STI	Intervention = 10.9%<br>Control = 3.4%	Net effect 7.5% = (-14.5; 29.5)				
Proportion of eligible patients who received emergency contraception	Pre-intervention = 50%<br>Post-intervention = 82%<br>p<0.01	-	60 patients (UBA/3 months)	Smith 2013[54]	High	4-day training, infrastructure improvement contributed towards improvement on 6 out of 10 indicators of post-rape care service
Proportion of eligible patients who received HIV post-exposure prophylaxis	Pre-intervention = 42%<br>Post-intervention = 92%<br>p<0.001	-				
Proportion of eligible patients who received STI prophylaxis and treatment	Pre-intervention = 45%<br>Post-intervention = 96%<br>p<0.01	-				
**VAW disclosure rate (n=4)**						
Proportion (n) who disclosed IPV of those screened, post-intervention	Intervention = 32% (34/107)<br>Control = 7% (9/134)<br>p<0.0001	-	337 women (RCT/1 month)	Haberland 2016[38]	High	HCP training and ongoing support, 29-minute integrated HIV-IPV consultation, referral to IPV counsellor in ANC clinic increased IPV identification rate
Proportion (n) who identified at least one IPV during past 3 months	Pre-intervention = 73.3% (299)<br>Post-intervention = 98.5% (402)<br>p<0.001	-	408 HCPs (UBA/6 months)	Jayatilleke 2015[39]	High	4-day training, handbook increased IPV identification rate
Proportion who disclosed IPV of those screened	62%	-	102 women (Cross-sectional/3 weeks)	Laisser 2011[45]	High	HCP training, infrastructure improvement, integrated ANC-IPV consultation, external referral contributed towards 62% IPV identification rate
Proportion (n) who disclosed IPV of those screened	8% (95/1210)	-	1210 women (Cross-sectional/7 months)	Undie 2016[57]	High	HCP training, integrated HIV-IPV consultation, assisted onsite referral contributed towards 8% IPV identification rate
**VAW referral uptake (n=2)**						
Proportion (n) who used GBV centre out of those referred, post-intervention	Intervention = 63% (12/19)<br>Control = 100% (3/3)<br>p=0.52	-	337 women (RCT/1 month)	Haberland 2016[38]	High	HCP training and ongoing support, 29-minute integrated HIV-IPV consultation, referral to IPV

Outcomes	Results (95% CI, p)	Effect (95% CI)	N of participants (design/follow up)	Study	Risk of bias	Direction of effect/interpretation
						counsellor in ANC clinic had no effect on uptake of referrals to on-site GBV centre
Proportion (n) who used GBV centre out of those referred, post-intervention	40% (29/73)	-	1210 women (Cross sectional/7 months)	Undie 2016[57]	High	HCP training, integrated IPV-HIV consultation, assisted onsite referral contributed towards 40% uptake of referrals to on-site GBV centre
**Use of SRH services (n=1)**						
Proportion (n) who were linked to medical care to receive lab reports on CD4 count and viral load, post-intervention	Intervention = 43.13% (69/160)<br>Control = 38.50% (30/78)<br>p = 0.493	-	166 women (RCT/1 month)	Brown 2018[32]	High	7-minute integrated HIV-IPV consultation over phone had no effect on uptake of HIV services among women with experience of IPV
**Safety behaviour (n=2)**						
Mean (SD) pre-post difference score of perceived risk and safety	Intervention = 0.33 (3.07)<br>Control = 0.13 (3.05)<br>p=0.278	-	166 women (RCT/1 month)	Brown 2018[32]	High	7-minute integrated HIV-IPV consultation over phone had no effect on perceived risk and safety among HIV-positive women with experience of IPV
Proportion (n) who used safety plan, post-intervention	Intervention = 61.88% (99/160)	-				Most HIV-positive women who received 7-minute integrated IPV-HIV consultation used safety plan and employed at least one safety strategy
Proportion (n) who employed at least one safety strategy	Intervention = 80% (128/160)	-				
Proportion (n) who took an action following the IPV-enhanced HIV counselling, post-intervention	Intervention = 45.5% (25/66)<br>Control = 30.5% (18/79)<br>p=0.073	-	337 women (RCT/1 month)	Haberland 2016[38]	High	HCP training and ongoing support, 29-minute integrated HIV-IPV consultation, referral to IPV counsellor in ANC clinic had no effect on coping behaviour and 7 behaviour indicators of HIV care among pregnant women
Proportion (n) who can ask partner to use a condom, post-intervention	Intervention = 58.3% (35/107)<br>Control = 51.2% (43/134)<br>p=0.31	-				
**Health outcomes**						
**Re-exposure to VAW (n=3)**						
Proportion (n) who did not experience IPV upon partner notification of serostatus, post-intervention	Intervention = 96.9% (155/160)<br>Control = 88% (71/79)	OR = 4.37 (1.46; 13.44)	166 HIV-positive women (RCT/1 month)	Brown 2018[32]	High	7-minute integrated HIV-IPV consultation over phone consultation reduced IPV upon partner notification about serostatus among HIV-positive women
Proportion (n) who experienced any IPV, post-intervention	Intervention = 16.0% (43/337)<br>Control = 18.7% (50/351), p=0.43	-	337 pregnant women (RCT/1 month)	Haberland 2016[38]	High	HCP training and ongoing support, 29-minute integrated HIV-IPV consultation, referral to IPV counsellor in ANC clinic had no effect on any IPV since baseline assessment
Mean (SD) danger assessment score	Pre-intervention = 6.02 (2.97)<br>Post-intervention = 2.82 (0.27)	MD = 3.20 (3.56) (2.43; 3.98)	84 women (UBA/3 months)	Matseke 2013	High	HPC training, 30-minute integrated ANC-IPV consultation, external referral contributed towards reduction in potential risk of becoming a victim of femicide among pregnant women

For peer review only

Outcomes	Results (95% CI, p)	Effect (95% CI)	N of participants (design/follow up)	Study	Risk of bias	Direction of effect/interpretation
**Harm (n=2)**						
Proportion (n) who reported that service had not placed them in greater danger, post-intervention	Intervention = 96.25% (154/160)<br>Control = 93.67% (74/79)<br>p = 0.512	-	166 women (RCT/1 month)	Brown 2018[32]	High	7-minute integrated HIV-IPV consultation over phone did not put HIV-positive women in greater danger
Proportion who reported harmful effects	Intervention = 0	-	337 pregnant women (RCT/1 month)	Haberland 2016[38]	High	HCP training and ongoing support, 29-minute integrated HIV-IPV consultation, referral to IPV counsellor in ANC clinic had no harmful effect on pregnant women

Note. VAW violence against women. GBV gender-based violence. DV domestic violence. IPV intimate partner violence. SRH sexual and reproductive health. STI Sexually transmitted infections. HIV human immunodeficiency virus. ANC antenatal care. HCP health care provider. RCT randomised controlled trial. CBA controlled before-after study. UBA uncontrolled before-after study. CI confidence interval. SD standard deviation. IQR interquartile range. MD mean difference. OR odds ratio.

For peer review only

Supplementary file 5. Effects and outcomes of interventions on response to VAW during routine SRH consultation plus community engagement

**Outcomes**	**Results**	**Effect (95% CI)**	**N of participants (design/follow up)**	**Study**	**Risk of bias**	**Direction of effect/interpretation**
**Direct effects on health-related cognition and emotions**						
**HCP knowledge about VAW and relevant procedures (n=2)**						
Proportion who knew whether there was law that deals with family violence	Pre-intervention = 71%<br>Post-intervention = 90%	19%	?HCPs (UBA/3 years)	Bott 2014[30]	High	Systems level activities, HCP training and ongoing support, infrastructure improvement, integrated family planning-GBV consultation, referral to onsite GBV specialist, community education contributed towards 19% increase in HCP knowledge about legal side of VAW
Proportion who could explain legal obligation of providers regarding family violence	Pre-intervention = 14%<br>Post-intervention = 69%	55%				
Proportion (n) who knew the main objectives of the programme	100% (35/35)		35 HCPs (Cross sectional/4-year service data)	Sithole 2018[53]	High	HCP training, infrastructure improvement, community education on post-rape care contributed towards 25% to 100% HCP awareness about post-rape care
Proportion of doctors who knew the tools to monitor the programme	25% (1/4)					
Proportion of doctors who knew the correct treatment guidelines	25% (1/4)					
Proportion of doctors who did not know the management process	75% (3/4)					
Proportion of nurses who knew the management process	100% (27/27)					
HCP attitudes about VAW (n=1)						
Reduction in proportion who blamed victims of physical and sexual violence (5 indicators), pre-post-intervention	-	By 29% for women provoke physical aggression<br>By 13% for men cannot control their sexual behaviour	?HCPs (UBA/3 years)	Bott 2014[30]	High	Systems level activities, HCP training and ongoing support, infrastructure improvement, integrated family planning-GBV consultation, referral to onsite GBV specialist, community education contributed towards 13-29% reduction in negative attitudes about GBV among HCPs
**HCP readiness for identifying and responding to VAW (n=1)**						
Reduction in proportion of cited 9 barriers to identifying IPV, pre-post intervention	-	By 29% for cultural divide between client and provider<br>By 3% for time constraints	?HCPs (UBA/3 years)	Bott 2014[30]	High	Systems level activities, HCP training and ongoing support, infrastructure improvement, integrated family planning-GBV consultation, referral to onsite GBV specialist, community education contributed 3% to 29% reduction in perceived barriers to identifying GBV among HCPs

For peer review only

Outcomes	Results	Effect (95% CI)	N of participants (design/follow up)	Study	Risk of bias	Direction of effect/interpretation
Increase in proportion who felt prepared to provide counselling about emergency contraception to GBV victims	-	By 96% for counselling about emergency contraception				Systems level activities, HCP training and ongoing support, infrastructure improvement, integrated family planning-GBV consultation, referral to onsite GBV specialist, community education contributed towards 95% increase in HCP preparedness to identify and respond to GBV patients
**Women attitudes about VAW (n=1)**						
Proportion (n) who justified husband physical abuse because of childcare, post-intervention	Intervention = 41.8% (261/625)<br>Control = 45.5% (284/624)	OR = 0.81 (0.60; 1.09)	656 women (cluster RCT/28 months)	Settergren 2018[60]	High	Systems level activities, HCP training, infrastructure improvement, integrated HIV-GBV consultation, onsite and external referral, community and couple education improved 1 out of 5 indicators of women's accepting attitudes towards VAW
Proportion (n) who justified husband physical abuse because she refuses to have sex with her partner, post-intervention	Intervention = 21.0% (131/625)<br>Control = 23.7% (148/624)	OR = 0.65 (0.46; 0.91)				
Mean (SD) score of the Violence domain of the Gender Equitable Men Scale, post-intervention	Intervention = 13.17 (3.98)<br>Control = 12.51 (3.93)	MD = 1.08 (0.52; 1.65)				Systems level activities, HCP training, infrastructure improvement, integrated HIV-GBV consultation, onsite and external referral, community and couple education improved women's attitudes towards more equitable gender roles
Mean (SD) score of the Domestic chores and daily life domain domain of the Gender Equitable Men Scale, post-intervention	Intervention = 8.74 (3.63)<br>Control = 7.62 (3.14)	MD = 1.26 (0.81; 1.71)				
**Intermediate effects on health-related behaviour and ptractices**						
**VAW enquiry rate (n=2)**						
Proportion (n) who received GBV screening and counselling, post-intervention	Intervention = 88.5% (1251/1413)<br>Control = 91/7% (442/482)<br>p=0.785	-	656 women (cluster RCT/28 months)	Settergren 2018[60]	High	Systems level activities, HCP training, infrastructure improvement, integrated HIV-GBV consultation, onsite and external referral, community and couple education had no effect on GBV enquiry rate
Proportion (n) who were screened for IPV of those attended clinic	94.5% (171/181)	-	171 women (Cross-sectional/4 months)	Samandari 2016[49]	High	System level activities, 7-day HCP training and ongoing support, integrated family planning-IPV consultation, external referral, community education contributed towards 95% IPV enquiry rate
**Provision of VAW referrals (n=2)**						
Proportion (n) who were referred to safe house and shelter of those screened, post-intervention	Intervention = 12.3% (173/1412)<br>Control = 2.3% (11/488)<br>p=0.216	-	656 women (cluster RCT/28 months) (cluster RCT/28 months)	Settergren 2018[60]	High	Systems level activities, HCP training, infrastructure improvement, integrated HIV-GBV consultation, onsite and external referral, community and couple education

For peer review only

Outcomes	Results	Effect (95% CI)	N of participants (design/follow up)	Study	Risk of bias	Direction of effect/interpretation
						had no effect on rates of referrals to safe house and shelter
Proportion (n) who were signposted to IPV services of those disclosed	100% (157/157)	-	171 women (Cross-sectional/4 months)	Samandari 2016[49]	High	System level activities, 7-day HCP training and ongoing support, integrated family planning-IPV consultation, external referral, community education contributed towards 100% signposting to IPV services
**Provision of safety planning (n=1)**						
Proportion (n) who received safety planning of those disclosed IPV	87.3% (137/157)	-	171 women (Cross-sectional/4 months)	Samandari 2016[49]	High	System level activities, 7-day HCP training and ongoing support, integrated family planning-IPV consultation, external referral, community education contributed towards 87% safety planning rate
**Provision of post-rape care (n=3)**						
Mean number of rape cases presenting to hospital per month.	Pre-intervention = 8<br>Post-intervention = 13	-	334 survivors of sexual assault (UBA and cross-sectional/not reported)	Kim 2007[42]	High	Systems level activities, 2-day HCP training, infrastructure improvement, community education on post-rape care contributed towards increased number of rape cases presenting to hospital
Proportion (n) of eligible patients who received post-rape medical kit	100% (2,081/2,081)	-	13 sites, 2081 patients (Cross-sectional/4-year service data)	Bress 2018[31]	High	HCP training and ongoing support, infrastructure improvement, community education on post-rape care contributed towards 100% provision of post-rape care medical kit
Change in proportion who attended within 72 hours, over 4 years	-	46%	80 HCPs, 1669 patients (Cross-sectional/4 years)	Sithole 2018[53]	High	HCP training, infrastructure improvement, community education on post-rape care contributed towards improvement on 6 indicators of post-rape care provision
Change in proportion who received HIV post-exposure prophylaxis, over 4 years	-	31%				
Change in proportion who received counselling, over 4 years	-	65%				
Change in proportion who received HIV testing, over 4 years	-	96.4%				
Change in proportion who received emergency contraception, over 4 years	-	8%				
Change in proportion who received STI prophylaxis	-	26%				
**VAW disclosure rates (n=2)**						
Proportion who disclosed GBV of those screened	14%	-	? women (UBA/3 years)	Bott 2004[30]	High	Systems level activities, HCP training and ongoing support, infrastructure improvement, integrated family planning-GBV consultation, referral to onsite GBV specialist, community education contributed towards 14% GBV identification rate

For peer review only

Outcomes	Results	Effect (95% CI)	N of participants (design/follow up)	Study	Risk of bias	Direction of effect/interpretation
Proportion (n) who disclosed IPV of those screened	91.8% (157/171)	-	171 women (Cross-sectional/4 months)	Samandari 2016[49]	High	System level activities, 7-day HCP training and ongoing support, integrated family planning-IPV consultation, external referral, community education contributed towards 92% IPV identification rate
Proportion (n) who disclosed GBV of those screened	37% (49/134)	-	134 women (Cross-sectional/5 months)	Turan 2013[56]	High	40-hour HCP training, integrated ANC-GBV consultation, assisted external referral, community education contributed towards 37% IPV identification rate
**VAW referrals uptake (n=2)**						
Proportion who took referral of those disclosed GBV	30%	-	? women (UBA/3 years)	Bott 2004[30]	High	Systems level activities, HCP training and ongoing support, infrastructure improvement, integrated family planning-GBV consultation, referral to onsite GBV specialist, community education contributed towards 30% uptake of referrals
Proportion (n) who took referral of those disclosed IPV	0.6% (1/157)	-	171 women (Cross-sectional/4 months)	Samandari 2016[49]	High	System level activities, 7-day HCP training and ongoing support, integrated family planning-IPV consultation, external referral, community education contributed towards 0.6% uptake of external referrals
Proportion (n) who took referral of those disclosed GBV	53% (26/49)	-	134 women (Cross-sectional/5 months)	Turan 2013[56]	High	40-hour HCP training, integrated ANC-GBV consultation, assisted external referral, community education contributed towards 53% uptake of referrals
**Use of SRH services (n=1)**						
Proportion (n) who attended any antenatal care, post-intervention	Intervention = 88.7% (1597/1800)<br>Control = 82.4% (1526/1851)	RD = 0.063 (-0.044; 0.170)	1837 women (cluster RCT/12 months)	Cockcroft 2019[34]	Low	HCP training, infrastructure improvement, integrated DV-universal home visits that discussed domestic violence, heavy work in pregnancy, ignorance of danger signs, and lack of spousal communication with pregnant women and their spouses had no effect on 8 indicators of use of antenatal care, institutional delivery, or skilled birth attendance
Proportion (n) who delivered in a health facility, post-intervention	Intervention = 30.1% (475/1579)<br>Control = 21.9 (391/1785)	RD = 0.082 (-0.071; 0.235)				
Proportion (n) who delivered by a skilled health worker post-intervention	Intervention = 29.3 (463/1579)<br>Control = 22.7 (404/1783)	RD = 0.067 (-0.081; 0.214)				
Mean (SD of GBV client visits per facility, post-intervention	Intervention = 237.8 (110.58)<br>Control = 81.5 (46.09)<br>p=0.010	-	656 women (cluster RCT/28 months)	Settergren 2018[60]	High	Systems level activities, HCP training, infrastructure improvement, integrated HIV-GBV consultation, onsite and external referral, community and couple education

Outcomes	Results	Effect (95% CI)	N of participants (design/follow up)	Study	Risk of bias	Direction of effect/interpretation
						increased use of SRH services by women with experience of GBV
Proportion (n) of GBV clients who received HIV testing and counselling, post-intervention	Intervention = 73.3% (1038/1416)<br>Control = 20.9% (102/488)<br>p=<0.001	-				Systems level activities, HCP training, infrastructure improvement, integrated HIV-GBV consultation, onsite and external referral, community and couple education increased use of 4 (HIV testing and counselling, family planning, forensic exam) out of 24 SRH services
Proportion (n) of GBV clients who received STI test, post-intervention	Intervention = 21.8% (308/1415)<br>Control = 11.5% (56/488)<br>p=0.128	-				
Safety behaviour (n=1)						
Proportion (n) of women who used condom, post-intervention	Intervention = 17.0% (157/931)<br>Control = 16.0% (192/1170)	PRR = 1.03 (0.85; 1.25)<br>aPRR = 1.01 (0.84; 1.21)	6 facilities, 1812 women (cluster RCT/35 months)	Wagman 2015[59]	High	HCP training, integrated HIV-IPV consultation, onsite referral, community education had no effect on 6 indicators of risk behaviours among women
**Health outcomes**						
**Re-exposure to VAW (n=3)**						
Proportion (n) who did not experience physical domestic violence during pregnancy, post-intervention	Intervention = 97.4 (1772/1820)<br>Control = 90.9 (1677/1844)	RD = 0.064 (0.045; 0.084)	1837 women (RCT/12 months)	Cockcroft 2019[34]	Low	HCP training, infrastructure improvement, integrated DV-universal home visits that discussed domestic violence, heavy work in pregnancy, ignorance of danger signs, and lack of spousal communication with pregnant women and their spouses reduced physical domestic violence
Proportion (n) who experienced any IPV in past 12 months, post-intervention	Intervention = 37.2% (207/556)<br>Control = 45.7% (268/587)	OR = 0.85 (0.62; 1.16)	6 facilities, 735 households (cluster RCT/28 months)	Settergren 2018[60]	High	Systems level activities, HCP training, infrastructure improvement, integrated HIV-GBV consultation, onsite and external referral, community and couple education had no effect on any IPV
Proportion (n) who experienced physical IPV in past 12 months, post-intervention	Intervention = 12% (217/1812)<br>Control = 16% (346/2127)	PRR = 0.74 (0.63; 0.86)<br>aPRR = 0.79 (0.67; 0.92)	6 facilities, 1812 women (cluster RCT/35 months)	Wagman 2015[59]	High	HCP training, integrated HIV-IPV consultation, onsite referral, community education reduced incidents of physical and sexual IPV and had no effect on emotional IPV.
Proportion (n) who experienced emotional IPV in past 12 months, post-intervention	Intervention = 18% (311/1737)<br>Control = 20% (409/2039)	PRR = 0.89 (0.78; 1.02)<br>aPRR = 0.91 (0.79; 1.04)				
Proportion (n) who experienced sexual IPV in past 12 months, post-intervention	Intervention = 10% (167/1737)<br>Control = 13% (261/2038)	PRR = 0.75 (0.62; 0.90)<br>aPRR = 0.80 (0.67; 0.97)				
**Sexual and reproductive health (n=1)**						

Outcomes	Results	Effect (95% CI)	N of participants (design/follow up)	Study	Risk of bias	Direction of effect/interpretation
Proportion (n) who did not have swelling of face or hands, post-intervention	Intervention = 97.4% (1790/1837)<br>Control = 71.1% (1317/1853)	RD = 0.264 (0.194; 0.333)	1837women (RCT/12 months)	Cockcroft 2019[34]	Low	HCP training, infrastructure improvement, integrated DV-universal home visits that discussed domestic violence, heavy work in pregnancy, ignorance of danger signs, and lack of spousal communication with pregnant women and their spouses improved 9 out of 13 indicators of pregnancy and postpartum complications
Proportion (n) who did not have raised blood pressure, post-intervention	Intervention = 96.6% (1409/1458)<br>Control = 85.1% (1269/1492)	RD = 0.116 (0.042; 0.190)				
Proportion (n) who did not have post-partum sepsis, post-intervention	Intervention = 81.1% (1478/1822)<br>Control = 48.8% (903/1852)	RD = 0.324 (95% CI 0.115; 0.493)				
Incidence of HIV per100 person-years	Intervention = 0.99<br>Control = 1.15	IRR = 0.86 (0.61; 1.22<br>aIRR = 0.72 (0.49; 1.07))	6 facilities, 1812 women (cluster RCT/35 months)	Wagman 2015[59]	High	HCP training, integrated HIV-IPV consultation, onsite referral, community education had no effect on incidence of HIV among women

Note. VAW violence against women. GBV gender-based violence. DV domestic violence. IPV intimate partner violence. SRH sexual and reproductive health. STI Sexually transmitted infections. HIV human immunodeficiency virus. ANC antenatal care. HCP health care provider. RCT randomised controlled trial. CBA controlled before-after study. UBA uncontrolled before-after study. CI confidence interval. SD standard deviation. IQR interquartile range. MD mean difference. OR odds ratio. RD risk difference. IRR incidence rate ratio.

For peer review only

Supplementary file 6. Effects and outcomes of interventions on response to VAW in addition to routine SRH consultation

Outcomes	Results	Effect (95% CI)	N of participants (design/follow up)	Study	Risk of bias	Direction of effect/interpretation
**Direct effects on health-related cognition and emotions**						
**Women knowledge about VAW (n=1)**						
Proportion who recognised violence as an issue of power, post-intervention	60.6%		155 women (UBA/ First prenatal appointment to 6 weeks after delivery)	Arora 2019[29]	High	After 2 or more 30-45-minute psychosocial counselling sessions, around 60% of pregnant women were aware about domestic violence and its impact on health
Proportion who recognised the impact of violence on health, post-intervention	65.5%					
**Women readiness for addressing VAW (n=1)**						
Proportion who recognised the need to take steps to address violence, post intervention	59.9%		155 women (UBA/ First prenatal appointment to 6 weeks after delivery)	Arora 2019[29]	High	After 2 or more 30-45-minute psychosocial counselling sessions, 60% of pregnant women were ready to address VAW
**Intermediate effects on health-related behaviour and practices**						
**VAW referral uptake (n=1)**						
Proportion (n) who used specialist IPV services, post-intervention	Intervention = 0.96% (1/104)<br>Control = 1.00% (1/100)	-	110 women (RCT/Prenatal appointment to 1 week after delivery)	Cripe 2010[35]	Some concerns	1 30-minute psychosocial counselling session, resource card, external referral had no effect on uptake of external referrals
**Use of SRH services (n=1)**						
Proportion (n) who missed antiretroviral medication, post-intervention	Intervention = 42.3% (19)<br>Control = 36.4% (25)	-	32 women (RCT/6 months)	Sikkema 2018[52]	High	7 90-minute psychosocial sessions on coping had no effect on engagement with HIV treatment among women with a history of sexual violence
Proportion (n) with high levels of non-retention in care, post-intervention	Intervention = 42.3% (26)<br>Control = 33.3% (27)	-				
**Use of non-health services (n=1)**						
Proportion (n) who used legal services, post-intervention	Intervention = 1.92% (2/104)<br>Control = 3.00% (3/100)	-	110 women (RCT/Prenatal appointment to 1 week after delivery)	Cripe 2010[35]	Some concerns	1 30-minute psychosocial counselling session, resource card, external referral had no effect on use of community resources among pregnant women
Proportion (n) who used police, post-intervention	Intervention = 0.96% (1/104)<br>Control = 4.00% (4/100)	-				
Proportion (n) who used social services, post-intervention	Intervention = 1.92% (2/104)<br>Control = 2.00% (2/100)	-				
**Safety behaviour (n=4)**						
Mean (SD) score of using safety behaviours, post-intervention	Intervention = 9.50 (2.63)<br>Control = 7.74 (2.42)	MD = 2.41 (1.43; 3.40)	70 women (RCT/Prenatal appointment to 6 weeks after delivery)	Sapkota 2020[50]	Low	1 35-45-minute psychosocial counselling session, resource card, contact details of the counsellor increased use of safety behaviours among pregnant women

Outcomes	Results	Effect (95% CI)	N of participants (design/follow up)	Study	Risk of bias	Direction of effect/interpretation
Proportion who adopted safety behaviours, post-intervention	Intervention = 30.3%<br>Control = 11.2%		110 women (RCT/Prenatal appointment to 1 week after delivery)	Cripe 2010[35]	Some concerns	1 30-minute psychosocial counselling session, resource card, external referral had no effect on safety behaviours among pregnant women
Mean (SD) score of avoidance, coping post-intervention	Intervention = 2.17 (0.13)<br>Control = 1.99 (0.09)	-	32 women (RCT/6 months)	Sikkema 2018[52]	High	7 90-minute psychosocial training sessions reduced avoidance coping, but had no effect on social coping among women with a history of sexual violence
Mean (SD) score of social coping, post-intervention	Intervention = 2.90 (0.10)<br>Control = 2.58 (0.10)	-				
Proportion (n) who used adaptive coping strategies at individual level pre- and post-intervention	Pre-intervention = 51.4% (73)<br>Post-intervention = 59.1% (84)<br>p=0.193		155 women (UBA/ First prenatal appointment to 6 weeks after delivery)	Arora 2019[29]	High	2 or more 30-45-minute psychosocial counselling sessions had no effect on coping behaviours among pregnant women
Proportion who used adaptive coping strategies at informal and formal levels pre- and post-intervention	Pre-intervention = 85.2% (121)<br>Post-intervention = 86.6% (123)<br>p=0.832					
**Health outcomes**						
**Re-exposure to VAW (n=4)**						
Mean (SD) score of verbal and physical IPV, post-intervention	Intervention = 11.62 (2.05)<br>Control = 13.28 (1.94)<br>p<0.001	-	50 pregnant women (RCT/2 months)	Khalili 2019[40]	High	4 90-minute psychoeducational counselling sessions reduced verbal and physical IPV among pregnant women
Mean (SD) score of total IPV, post-intervention	Intervention = 17.70 (11.12)<br>Control = 31.22 (21.17)	MD = 13.51 (9.99; 17.02)	141 pregnant women (RCT/6 months)	Mutisya 2018[47]	High	1-3 30-35-minute psychosocial counselling sessions, risk assessment, safety planning, resource card, external referral reduced IPV among pregnant women
Proportion who experienced physical IPV, post intervention	Intervention = 51.2%<br>Control = 65.9%	RR = 0.78 (0.63; 0.93)	125 women (RCT/3 months)	Tanghizaden 2018[55]	Some concerns	4 90-minute psychosocial training sessions on problem-solving skills reduced physical and psychological IPV but had no effect on sexual IPV among pregnant women
Proportion who experienced psychological IPV, post intervention	Intervention = 67.4%<br>Control = 92.4%	RR = 0.73 (0.64; 0.83)				
Proportion who experienced sexual IPV, post intervention	Intervention = 50.4%<br>Control = 57.6%	RR = 0.87 (0.69; 1.09)				
Change in proportion who experienced physical domestic violence, before-after	74.6% to 3.5%	-	155 women (UBA/ First prenatal appointment to 6 weeks after delivery)	Arora 2019[29]	High	2 or more 30-45-minute psychosocial counselling sessions reduced physical, emotional, and financial domestic violence among pregnant women

For peer review only

Outcomes	Results	Effect (95% CI)	N of participants (design/follow up)	Study	Risk of bias	Direction of effect/interpretation
Change in proportion who experienced emotional domestic violence, before-after	98.6% to 34.5%	-				
Change in proportion who experienced financial domestic violence, before-after	72.5% to 11.3%	-				
**Mental health (n=6)**						
Mean (SD) score of anxiety, post-intervention	Intervention = 4.33 (3.84)<br>Control = 6.93 (4.87)	MD = -3.73 (-5.42; -2.04)	70 women (RCT/prenatal appointment to 6 weeks after delivery)	Sapkota 2020[50]	Low	1 35-45-minute psychosocial counselling session, resource card, contact details of the counsellor reduced anxiety and depression among pregnant women
Mean (SD) score of depression, post-intervention	Intervention = 3.51 (3.46)<br>Control = 6.13 (3.68)	MD = -3.41 (-4.84; -1.99)				
Mean (SD) score of postnatal depression, post-intervention	Intervention = 5.34 (4.23)<br>Control = 12.46 (4.22)	MD = 7.12 (6.21; 8.03)	141 women (RCT/6 months)	Mutisya 2018[47]	High	1-3 30-35-minute psychosocial counselling sessions with risk assessment, safety planning, external referral, and resource card reduced depression among pregnant women
Mean (SD) score of PTSD, post-intervention	Intervention = 28.61 (5.04)<br>Control = 22.50 (3.47)	-	32 women (RCT/6 months)	Sikkema 2018[52]	High	7 90-minute psychosocial training sessions had no effect on PTSD symptoms among women with a history of sexual violence
Mean (SD) score of psychological distress, post-intervention	Intervention = 22.28 (3.81)<br>Control = 24.06 (4.16)<br>p<0.001	-	50 women (RCT/2 months)	Khalili 2019[40]	High	4 90-minute psychoeducational counselling sessions reduced psychological distress among pregnant women
Difference between baseline and post-intervention mean (SD) score for mental health	Intervention = 2.50 (20.95)<br>Control = 2.04 (19.61)	MD = 4.54 (-1.07; 10.15)	110 women (RCT/Prenatal appointment to 1 week after delivery)	Cripe 2010[35]	Some concerns	1 30-minute psychosocial counselling session, resource card, external referral had no effect on mental health of pregnant women
Change in proportion who experienced any emotional health problems	96.5% to 33.1%	-	155 women (UBA/ First prenatal appointment to 6 weeks after delivery)	Arora 2019[29]	High	2 or more 30-45-minute psychosocial counselling sessions reduced % of pregnant women with emotional health problems.
**Sexual and reproductive health (n=1)**						
Proportion (n) of those with unsuppressed HIV viral load, post-intervention	Intervention = 15.8% (19)<br>Control = 20.0% (25)<br>($\chi2$ (1) = 0.13, p = 0.72)	-	32 women (RCT/6 months)	Sikkema 2018[52]	High	7 90-minute psychosocial training sessions had no effect on adherence to therapy measured by HIV viral load among women with a history of sexual abuse
**Physical health (n=2)**						
Difference between baseline and post-intervention mean (SD) score for general health	Intervention = 5.30 (15.62)<br>Control = 4.74 (14.67)	MD = 0.05 (-6.80; 7.79)	110 women (RCT/Prenatal appointment to 1 week after delivery)	Cripe 2010[35]	Some concerns	1 30-minute psychosocial counselling session, resource card, external referral had no effect on general health

Outcomes	Results	Effect (95% CI)	N of participants (design/follow up)	Study	Risk of bias	Direction of effect/interpretation
Change in proportion who experienced any physical health problems	54.6% to 10.5%	-	155 women (UBA/ First prenatal appointment to 6 weeks after delivery)	Arora 2019[29]	High	2 or more individual psychosocial counselling sessions reduced % of pregnant women with physical health problems.
**Quality of life (n=2)**						
Mean (SD) score of overall quality of life, post-intervention	Intervention = 17.22 (3.00)<br>Control = 15.19 (2.77)	MD = 2.45 (1.51; 3.39)	70 women (RCT/prenatal appointment to 6 weeks after delivery)	Sapkota 2020[50]	Low	1 35-45-minute psychosocial counselling session, resource card, contact details of the counsellor improved quality of life among pregnant women
Difference between baseline and post-intervention mean (SD) score for physical functioning	Intervention = -15.67 (28.35)<br>Control = -15.70 (25.06)	MD = 0.03 (-7.37; 7.42)	110 women (RCT/Prenatal appointment to 1 week after delivery)	Cripe et 2010[35]	Some concerns	1 30-minute psychosocial counselling session, resource card, external referral had no effect on general health, bodily pain, vitality, social functioning among pregnant women.
Difference between baseline and post-intervention mean (SD) score for bodily pain	Intervention = -7.40 (28.33)<br>Control = -7.90 (24.28)	MD = 0.50 (-6.80; 7.79)				
Difference between baseline and post-intervention mean (SD) score for vitality	Intervention = -0.19 (22.34)<br>Control = -3.65 (22.06)	MD = 3.46 (-2.67; 9.59)				
Difference between baseline and post-intervention mean (SD) score for social functioning	Intervention = -0.36 (34.94)<br>Control = -3.50 (37.06)	MD = 3.14 (-6.80; 13.08)				

Note. VAW violence against women. GBV gender-based violence. DV domestic violence. IPV intimate partner violence. SRH sexual and reproductive health. STI Sexually transmitted infections. HIV human immunodeficiency virus. ANC antenatal care. HCP health care provider. RCT randomised controlled trial. CBA controlled before-after study. UBA uncontrolled before-after study. CI confidence interval. SD standard deviation. IQR interquartile range. MD mean difference. RR relative risk.

For peer review only

Supplementary file 7. Barriers to identification and response to VAW in sexual and reproductive health services

Theme	Description	Discussed by	Supporting text
Acceptability of VAW	Attitudes and social norms that regulate the acceptability of VAW at individual and community levels	Cristofides 2010[33]<br>Laisser 2011[45]<br>Smith 2013[54]<br>Turan 2013[56]	**Women:** "Women did not want a referral because they did not feel the violence was serious or they felt these were personal issues that they will solve on their own"[38]<br>**HCPs:** "Some of these patients are themselves to be blamed. You know some women don't want to be polite to their husbands and adhere to the norms in their marriages that is why they are beaten. It takes time, need to be more patient and expertize to screen, which we miss. It may be too costly for training. (FGD3 Male Nurse)"[45]<br>**Community:** "So when somebody is saying that women are not supposed to be beaten, that. . . they should go to somebody and take some action, in the community it is like that person is acting against the will of the community. To the men it is like he is an outcast in the community, an outlaw who is not supposed to be there. . . . In social places you will hear them saying that he is not a good person because if he is preaching to our ladies and women to take action against us, then it is like he wants to bring a revolution, women are going to overpower us and then we are going to be voiceless. (Focus Group #1, Respondent #8)"[56]
Fear of negative consequences	Real or potential negative consequences (psychological, legal, financial) of engaging in VAW work that could make the situation worse for individuals and health system	Bott 2004[30]<br>Christofides 2010[33]<br>Haberland 2016[38]<br>Knettel 2019[44]<br>Laisser 2011[45]<br>Samandari 2016[49]<br>Sapkota 2020[51]<br>Sithole 2018[53]	**Women:** "The sessions would irritate me when we talked about my rape; I hated to talk about it even though when I had talked about it, I would feel better. My heart would feel sore. Even talking about my HIV status irritated me because I still beat myself for infecting my child"[44]<br>"a few women stated that privacy concerns made it difficult for them to participate in the intervention, especially the group sessions. One participant explained that she was "afraid that I might be seen by a participant who knows me and who might go around discussing my problems."[44]<br>**HCPs:** "Providers were responsible for all IPV screening and counseling, as well as their regular FP duties. This led not only to an increased burden of duty for providers, but also the experience of secondary trauma, resulting from the exposure to clients' IPV stories."[49]<br>**Healthcare system**: "In one shift we normally attend up to 60 plus in a room for the two clinicians. Sometimes we reach up to 100 clients when it is a busy day, but if we are to attend one client at a time then it will be only 15 clients per day in a room. Where will others go?" Male clinician"[45]
Limited readiness for VAW work in SRH services	Structural unreadiness within health system: lack of support from leadership, time pressure, insufficient budget, lack of adequate resources, limited privacy	Abeid 2016[28]<br>Bott 2004[30]<br>Christofides 2010[33]<br>Haberland 2016[38]<br>Laisser 2011[45]<br>Sammandari 2016[49]<br>Undie 2016[57]	"System level factors may have influenced the implementation of IPV screening by lay counsellors …Other factors included inadequate management and supervision, burn-out, and small stipends which adversely affect counsellors' motivation to do something perceived as extra."[33]<br>"The HCWs felt they had not much to offer to the women who were experiencing IPV. This category thus represents an uncertainty as to whether the health care system is ready for routine screening for IPV and suggests a need for reinforced organizational change."[45]<br>"Perceived barriers to replication and scale-up included inadequate funding, insufficient clinic staff, and lack of political commitment for IPV services on the part of MOHPH"[49]<br>"Providers' main criticism was the longer time required to conduct the enhanced counseling. It created delays in the system, frustrating clients who were tired of being at the hospital for so long. Providers also felt the effects of extra time. One noted explicitly that they are supposed to see a certain number of clients each day and if they do not meet their targets they will have problems with management."[38]<br>"D3: It is also difficult to examine a patient in front of another one even if we use curtains. There is one examination bed for two of us and when you ask questions about STD patients feel embarrassed. Although we try to use low voice, people like to listen to others' conversations. (FGD1 Female Clinician"[45]
	Wider systems unreadiness: lack of services to refer to, poor referral system, untrained staff in non-health services	Abeid 2016[28]<br>Bott 2004[30]<br>Christofides 2010[33]<br>Laisser 2011[45]	"Providing referrals to women who disclosed current experiences of IPV may be of limited utility where services are hard to access"[33]
	Society unreadiness: poverty, no money for transport fare and	Haberland 2016[38]<br>Knettel 2019[44]<br>Laisser 2011[45]	"D1: You know I have nothing much to say but would like to do the screening -the resources are my dilemma. Many women are poor 'wanyonge' and are not strong enough to fight with their husbands but maybe this would be their good start. They will be happier later in future. (FGD3 Female Clinician)"[45]

For peer review only

	healthcare services, no transport, financial dependence on husband.	Sithole 2018[53]	"Her issue was that the partner used to beat her and to abuse her physically when she asked for bus fare to come to the clinic. So you see at the end of the day if she doesn't get help to deal with the violence she won't be able to come to the clinic because she is being abused when she asks for money to come to the clinic and it will affect her overall outcome…"[38]<br>"Getting to the clinic would be a challenge as I do not work and often had to borrow money."[44]
	Women's unreadiness for VAW services offered by HCPs: the demand-supply gap between women's preferences for adequate response to VAW and what HCPs offered to them	Christofides 2010[33]<br>Haberland 2016[38]<br>Undie 2016[57]	"However, one woman questioned whether there was any point in talking to the lay counselor unless the counselor would go home and make her husband stop. Other participants, who had not disclosed abuse, suggested that health care providers could talk to a woman's abusive partner and this would stop the violence. Others suggested that if abusive partners knew about IPV screening they would stop. This seems perhaps unrealistic."[33]
			"Although the initial intention of the intervention was for IPV-positive clients to receive same-day services at the GBV clinic, this was not always possible, because women did not have the time, GBV clinic staff were not always available, and some clients preferred to have their initial GBV clinic appointment on a later date. There were occasions when providers referred clients to the GBV clinic and the clients initially complied with the referral, only to find that their needs could not be attended to immediately due to the unavailability of staff atthe GBV clinic."[57]

For peer review only

PRISMA 2020 Checklist

Section and Topic	Item #	Checklist item	Location where item is reported
**TITLE interventions in sexual and reproductive health services addressing violence against women in low- and middle- income countries: a mixed-methods systematic review**			
Title	1	Identify the report as a systematic review.	Title page
**ABSTRACT**			
Abstract	2	See the PRISMA 2020 for Abstracts checklist.	Abstract
**INTRODUCTION**			
Rationale	3	Describe the rationale for the review in the context of existing knowledge.	Introduction, para 1-2
Objectives	4	Provide an explicit statement of the objective(s) or question(s) the review addresses.	Introduction, para 3
**METHODS**			
Eligibility criteria	5	Specify the inclusion and exclusion criteria for the review and how studies were grouped for the syntheses.	Tab. 1
Information sources	6	Specify all databases, registers, websites, organisations, reference lists and other sources searched or consulted to identify studies. Specify the date when each source was last searched or consulted.	Search strategy and selection criteria, para 2
Search strategy	7	Present the full search strategies for all databases, registers and websites, including any filters and limits used.	Suppl file 1
Selection process	8	Specify the methods used to decide whether a study met the inclusion criteria of the review, including how many reviewers screened each record and each report retrieved, whether they worked independently, and if applicable, details of automation tools used in the process.	Search strategy and selection criteria, para 2
Data collection process	9	Specify the methods used to collect data from reports, including how many reviewers collected data from each report, whether they worked independently, any processes for obtaining or confirming data from study investigators, and if applicable, details of automation tools used in the process.	Data analysis, para 1
Data items	10a	List and define all outcomes for which data were sought. Specify whether all results that were compatible with each outcome domain in each study were sought (e.g. for all measures, time points, analyses), and if not, the methods used to decide which results to collect.	Tab. 3, Suppl files 4-6
	10b	List and define all other variables for which data were sought (e.g. participant and intervention characteristics, funding sources). Describe any assumptions made about any missing or unclear information.	Tab. 2
Study risk of bias assessment	11	Specify the methods used to assess risk of bias in the included studies, including details of the tool(s) used, how many reviewers assessed each study and whether they worked independently, and if applicable, details of automation tools used in the process.	Data analysis, para 2, Suppl file 2
Effect measures	12	Specify for each outcome the effect measure(s) (e.g. risk ratio, mean difference) used in the synthesis or presentation of results.	Suppl files 4-6

For peer review only

PRISMA 2020 Checklist

Section and Topic	Item #	Checklist item	Location where item is reported
Synthesis methods	13a	Describe the processes used to decide which studies were eligible for each synthesis (e.g. tabulating the study intervention characteristics and comparing against the planned groups for each synthesis (item #5)).	Tab. 2
	13b	Describe any methods required to prepare the data for presentation or synthesis, such as handling of missing summary statistics, or data conversions.	Data analysis, para 1
	13c	Describe any methods used to tabulate or visually display results of individual studies and syntheses.	Tab.3, Suppl files 4-6
	13d	Describe any methods used to synthesize results and provide a rationale for the choice(s). If meta-analysis was performed, describe the model(s), method(s) to identify the presence and extent of statistical heterogeneity, and software package(s) used.	Data analysis, para 3, Fig. 2
	13e	Describe any methods used to explore possible causes of heterogeneity among study results (e.g. subgroup analysis, meta-regression).	NA
	13f	Describe any sensitivity analyses conducted to assess robustness of the synthesized results.	NA
Reporting bias assessment	14	Describe any methods used to assess risk of bias due to missing results in a synthesis (arising from reporting biases).	Quality appraisal, para 1
Certainty assessment	15	Describe any methods used to assess certainty (or confidence) in the body of evidence for an outcome.	NA
**RESULTS**			
Study selection	16a	Describe the results of the search and selection process, from the number of records identified in the search to the number of studies included in the review, ideally using a flow diagram.	Fig. 1
	16b	Cite studies that might appear to meet the inclusion criteria, but which were excluded, and explain why they were excluded.	Fig. 1
Study characteristics	17	Cite each included study and present its characteristics.	Tab. 2
Risk of bias in studies	18	Present assessments of risk of bias for each included study.	Suppl file 2
Results of individual studies	19	For all outcomes, present, for each study: (a) summary statistics for each group (where appropriate) and (b) an effect estimate and its precision (e.g. confidence/credible interval), ideally using structured tables or plots.	Suppl files 4-6
Results of syntheses	20a	For each synthesis, briefly summarise the characteristics and risk of bias among contributing studies.	Quality appraisal, para 1
	20b	Present results of all statistical syntheses conducted. If meta-analysis was done, present for each the summary estimate and its precision (e.g. confidence/credible interval) and measures of statistical heterogeneity. If comparing groups, describe the direction of the effect.	Tab. 3
	20c	Present results of all investigations of possible causes of heterogeneity among study results.	NA
	20d	Present results of all sensitivity analyses conducted to assess the robustness of the synthesized results.	NA

For peer review only

PRISMA 2020 Checklist

Section and Topic	Item #	Checklist item	Location where item is reported
Reporting biases	21	Present assessments of risk of bias due to missing results (arising from reporting biases) for each synthesis assessed.	NA
Certainty of evidence	22	Present assessments of certainty (or confidence) in the body of evidence for each outcome assessed.	NA
**DISCUSSION**			
Discussion	23a	Provide a general interpretation of the results in the context of other evidence.	Discussion, para 1
	23b	Discuss any limitations of the evidence included in the review.	Discussion, para 5-8
	23c	Discuss any limitations of the review processes used.	Discussion para 2-3
	23d	Discuss implications of the results for practice, policy, and future research.	Discussion, para 4, 9
**OTHER INFORMATION**			
Registration and protocol	24a	Provide registration information for the review, including register name and registration number, or state that the review was not registered.	Abstract
	24b	Indicate where the review protocol can be accessed, or state that a protocol was not prepared.	Abstract
	24c	Describe and explain any amendments to information provided at registration or in the protocol.	NA
Support	25	Describe sources of financial or non-financial support for the review, and the role of the funders or sponsors in the review.	Funder, Disclaimer
Competing interests	26	Declare any competing interests of review authors.	Competing interests
Availability of data, code and other materials	27	Report which of the following are publicly available and where they can be found: template data collection forms; data extracted from included studies; data used for all analyses; analytic code; any other materials used in the review.	Data sharing statement

From: Page MJ, McKenzie JE, Bossuyt PM, Boutron I, Hoffmann TC, Mulrow CD, et al. The PRISMA 2020 statement: an updated guideline for reporting systematic reviews. BMJ 2021;372:n71. doi: 10.1136/bmj.n71

For more information, visit: http://www.prisma-statement.org/

For peer review only
